# Prevention and management of unprofessional behaviour among adults in the workplace: A scoping review

**DOI:** 10.1371/journal.pone.0201187

**Published:** 2018-07-26

**Authors:** Andrea C. Tricco, Patricia Rios, Wasifa Zarin, Roberta Cardoso, Sanober Diaz, Vera Nincic, Alekhya Mascarenhas, Sabrina Jassemi, Sharon E. Straus

**Affiliations:** 1 Dalla Lana School of Public Health, University of Toronto, Toronto, Ontario, Canada; 2 Knowledge Translation Program, Li Ka Shing Knowledge Institute, St. Michael’s Hospital, Toronto, Ontario, Canada; 3 Department of Medicine, University of Toronto, Toronto, Ontario, Canada; Middlesex University, UNITED KINGDOM

## Abstract

**Background:**

Unprofessional behaviour is a challenge in academic medicine. Given that faculty are role models for trainees, it is critical to identify strategies to manage these behaviours. A scoping review was conducted to identify interventions to prevent and manage unprofessional behaviour in any workplace or professional setting.

**Methods:**

A search of 14 electronic databases was conducted in March 2016, reference lists of relevant systematic reviews were scanned, and grey literature was searched to identify relevant studies. Experimental and quasi-experimental studies that reported on interventions to prevent or manage unprofessional behaviours were included. Studies that reported impact on any outcome were eligible. Two reviewers independently screened articles and completed data abstraction. Qualitative analysis of the definitions of unprofessional behaviour was conducted. Data were charted to describe the study, participant, intervention and outcome characteristics.

**Results:**

12,482 citations were retrieved; 23 studies with 11,025 participants were included. The studies were 12 uncontrolled before and after studies, 6 controlled before and after studies, 2 cluster-randomised controlled trials (RCTs), 1 RCT, 1 non-randomised controlled trial and 1 quasi-RCT. Four constructs were identified in the definitions of unprofessional behaviour: verbal and/or non-verbal acts, repeated acts, power imbalance, and unwelcome behaviour. Interventions most commonly targeted individuals (22 studies, 95.7%) rather than organisations (4 studies, 17.4%). Most studies (21 studies, 91.3%) focused on increasing awareness. The most frequently targeted behaviour change was sexual harassment (4 of 7 studies).

**Discussion:**

Several interventions appear promising in addressing unprofessional behaviour. Most of the studies included single component, in-person education sessions targeting individuals and increasing awareness of unprofessional behaviour. Fewer studies targeted the institutional culture or addressed behaviour change.

## Introduction

Unprofessional behaviour, including bullying, has become a major issue in recent international news [1–3]. Academic medicine is not immune to unprofessional behaviour; it has been reported by medical students, residents and faculty [4–7]. A systematic review showed that almost 60% of medical students experienced at least one form of harassment or discrimination and the most common perpetrator was the consultant physician. Similarly, a review of resident mistreatment found that physicians of higher hierarchical power were the most common perpetrators [8]. Surveys of physicians in various countries [7, 9–12] have shown that up to 98% have experienced unprofessional behaviour in the workplace. While the commonest perpetrators are patients or their families, it is not uncommon for co-workers or supervisors to be the perpetrators [7, 9–12].

The impact of unprofessional behaviour on victims is widespread and concerning. Workplace abuse is associated with stress, depression, anxiety and absence from work in those who experience it [13–17]. Of particular concern in health care is the impact of unprofessional behaviour on role modeling for trainees and on patient care [14].

Unprofessional behaviour is recognised as an institutional challenge in academic organisations [14, 18]. Some authors have suggested that it is embedded within a medical culture that perpetuates the cycle of unprofessionalism [14, 18]. As faculty members are role models for their trainees, it is critical that we understand strategies to prevent and manage these behaviours. Previous attempts to mitigate unprofessional behaviour include feedback to perpetrators and educational interventions [19, 20]. However, the effectiveness of these strategies, in particular targeting faculty in academic medical centres and universities, is not clear. As such, the aim of this scoping review was to identify interventions to prevent and manage unprofessional behaviour among adults in any workplace or professional setting.

## Methods

We developed a protocol using the scoping review methods proposed by Arksey and ‘O’Malley [21] and further refined by the Joanna Briggs Institute [22]. The review was conducted to inform the efforts of the Department of Medicine (DOM), University of Toronto, whose members provided feedback on the protocol. We registered the review through the Open Science Framework [23]. Although the PRISMA statement has not been modified for scoping reviews, we used it to guide our reporting (S1 Table) [24].

### Eligibility criteria

Our eligibility criteria were defined using the ‘Population, Intervention, Comparison, Outcomes, Study designs, Timeframe’ (PICOST) components [25] and were verified by members of the Department of Medicine, University of Toronto.

Population: All individuals employed full-time or part-time in any workplace setting. Our preliminary literature searches indicated that few interventions were tested in academic medicine, which is why the scope was broadened to include any workplace setting.

Interventions: Interventions to prevent and manage unprofessional behaviours in the workplace were eligible. A preliminary search for systematic reviews revealed no standard definition or list of terms used to describe unprofessional behaviour (e.g. workplace bullying) but common themes identified in this preliminary review were used to inform the search, which are provided in S2 Table. Similarly, various unprofessional behaviours were considered ranging from ignoring phone calls from co-workers to verbal hostility, social exclusion, sexual harassment, and threats to professional status [26, 27].

Comparators: Usual care, other interventions or no intervention were eligible for inclusion.

Outcomes: All relevant outcomes were eligible for inclusion, such as institutional culture, prevalence of unprofessional behaviours, as well as retention and recruitment of staff, faculty or trainees.

Study designs: We included all experimental (randomised controlled trials (RCTs), quasi-RCTs), quasi-experimental (interrupted time series, controlled before and after) and observational (cohort, case control) studies. Systematic reviews and qualitative studies were not eligible for inclusion. No limitations were imposed with regard to year of publication, language or publication status.

### Information sources and search strategy

An experienced information specialist (JM) developed our comprehensive literature search in consultation with the research team, which was executed by a library technician (AE). ‘Seed’ papers (or seminal papers in the field) were identified by Department of Medicine members to validate the search strategy. The search was peer reviewed by a second librarian (EC) using the PRESS Checklist [28]. The search was revised and executed from inception until March 28, 2016 in the following databases: MEDLINE, EMBASE, CINAHL, The Cochrane Library, The Campbell Library, The Joanna Briggs Institute, Education Resources Information Centre (ERIC), PsycInfo, Social Work Abstracts, Sociological Abstracts, Dissertation Abstracts International, Dissertations & Theses Global, Criminal Justice Abstracts, National Criminal Justice Reference Service Abstracts, Business Source Complete, and ABI/IFNORM (S1 Text). We also conducted a search to identify difficult to locate and unpublished (or grey) literature using the Canadian Agency for Drugs and Technologies in Health checklist, [29] such as websites (e.g., Bullying Research Network, Workplace Bullying Institute) and thesis databases (S2 Text). To identify other potentially relevant articles, we asked experts in the field and searched the references of relevant systematic reviews identified during screening.

### Study selection

We removed duplicates and imported the literature search results into our proprietary software for screening titles and abstracts and full text articles [30]. The inclusion criteria were used for screening citations during the title and abstract screening (i.e., level 1) and full-text article (i.e., level 2) screening. We completed calibration exercises prior to each stage of screening to ensure reliability across reviewers. Inter-rater agreement was calculated using percent agreement and set at a threshold of ≥75%. For level 1 screening, one pilot-test of 50 citations was conducted and 80% agreement was achieved. Subsequently, pairs of independent reviewers reviewed all titles and abstracts for inclusion (RC, SD, VN, PR, WZ). For full-text screening, one pilot-test of 25 full-text articles was conducted with 76% agreement achieved. Reviewer pairs then independently screened the full-text of potentially relevant articles to determine inclusion (RC, SD, VN, PR, WZ). The results of the grey literature search were screened using the same process. All discrepancies between reviewers were resolved by a third reviewer (PR, WZ).

### Data abstraction

We drafted a data abstraction form with feedback from the Department of Medicine members. Abstracted data included study characteristics (e.g. country of conduct, setting, study design), population characteristics (e.g. type of participant, mean age, % female, expertise), intervention and control characteristics (e.g. description of program, target group, intensity), and types of outcomes (e.g. staff retention, prevalence of unprofessional behaviours, institutional culture). After pilot-testing the data abstraction form on 3 studies, pairs of independent reviewers abstracted the data (RC, SD, VN, PR, WZ); all data were then verified by a third reviewer (RC, PR, WZ).

We did not conduct risk of bias assessment of included articles as per the Joanna Briggs Institute Methods Manual for Scoping Reviews, [22] which is consistent with scoping reviews on health-related topics [31].

### Synthesis

The synthesis focused on describing approaches used to prevent or manage unprofessional behaviour. We charted the data quantitatively to identify the number of relevant publications according to types of participants, interventions, comparators and outcomes and summarised these findings using descriptive frequencies. We completed a thematic analysis of the definitions for unprofessional behaviour, as we identified many variations used across the studies. Two experienced qualitative analysts (AM, SJ) conducted a familiarization activity by independently listing and naming ideas from the list of definitions. These ideas were then grouped into 4 constructs. Those ideas related to the effects of incivility (e.g., hostile work environment, impact on work performance) and the causes of incivility (e.g., jealousy, taking out dissatisfaction on others) were excluded. The analysts then coded the definitions using the four headings in NVivo 11[32]. Kappa coefficients were calculated to measure agreement in the coding scheme.

### Consultation

We provided regular updates to the Department of Medicine and provided the review results to them for their interpretation.

## Results

**Fig 1:**
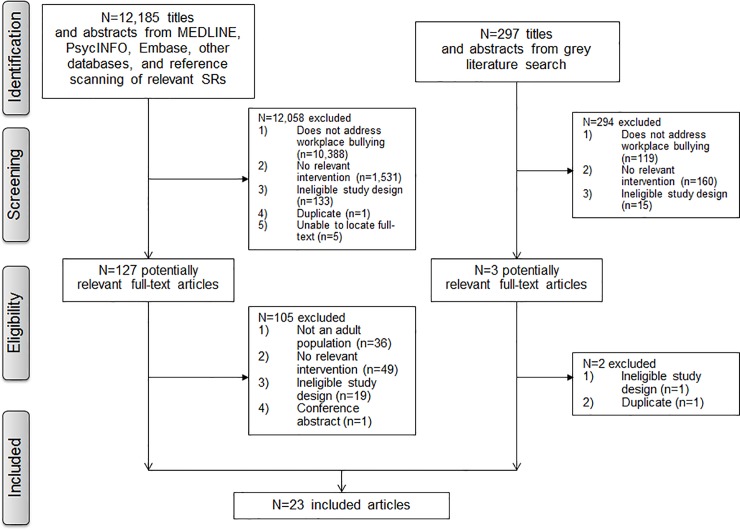
Study flow diagram.

We retrieved 12,482 citations from the electronic database search (11,689), grey literature search (299), and reference scanning of relevant systematic reviews (515) (Fig 1). Of these, 130 citations were potentially relevant and their full-texts were reviewed (102 citations from database search, 25 from reference scanning, and 3 from grey literature). Subsequently, 23 articles met our eligibility criteria (17 articles from database search, 5 from reference scanning and 1 from grey literature).

### Quantitative charting

#### Publication and participant characteristics

The 23 studies included 12 uncontrolled before and after studies, [33–44] 6 controlled before and after studies, [45–50] 2 cluster-RCTs, [51, 52] 1 RCT, [53] 1 NRCT [54] and 1 quasi-RCT [55] (Table 1, S3 Table). Fourteen studies were conducted in the US [34–39, 42, 43, 45, 47, 50, 51, 53, 55], 3 each in Canada [46, 49, 54] and the UK [41, 48, 52], and 1 each in Israel [33], Spain [44] and Australia [40]. Four studies reported use of an active comparator [48, 49, 52, 53] while the remainder either had no comparator or a standard practice comparator. Eleven studies (47.8%) were performed in health care organisations [34–37, 40, 42, 43, 45, 49, 50, 54], 5 in education settings (21.7%) [38, 39, 46, 51, 55], 4 in government settings (17.4%) [33, 41, 47, 52] and 3 in private industry (13.0%) [44, 48, 53]. A total of 11,025 participants were included with studies ranging from 16 to 4,032 participants. Of the 11 studies that reported sex of participants, 1% to 96.2% were female [33, 35, 38, 39, 43, 45, 46, 51, 53–55]. Of the 4 studies that reported ethnicity of participants, the majority were Caucasian [35, 45, 53, 55]. The majority of the studies (n = 18 [78%]) targeted staff or line workers, followed by middle managers (n = 10 [43.8%]) [S4 Table].

**Table 1:** Summary study characteristics.

Characteristic	Number of studies N = 23 (%)
**Study Design**	
Pre-/Post- Test Survey (single group design)	12 (52.17%)
Controlled Before and After Study	6 (26.08%)
Cluster-Randomised Controlled Trial	2 (8.70%)
Non-randomised Controlled Trial	1 (4.35%)
Quasi-Randomised Controlled Trial	1 (4.35%)
Randomised Controlled Trial	1 (4.35%)
**Type of Comparator**^**a**^	
No Comparator	12 (52.17%)
Standard Practice/No Intervention	11 (47.83%)
Active Comparator	4 (17.39%)
**Type of Analysis**	
Quantitative	20 (86.96%)
Mixed methods	3 (13.04%)
**Type of Organization**	
Healthcare Organization	11 (47.83%)
Educational Institution	5 (21.74%)
Public Administration/Government	4 (17.39%)
Private Company	3 (13.04%)
**Type of Employee**^**b**^	
Staff/Line Worker	18 (78.26%)
Supervisor/Middle Management	10 (43.48%)
Executive/Upper Management	1 (4.35%)
Other	6 (26.09%)
**Type of Intervention**^**b**^	
Awareness/Education	21 (91.30%)
Conflict Resolution Training	8 (34.78%)
Assertiveness Training	4 (17.39%)
Policy/Code of Conduct	3 (13.04%)
Reporting Systems	2 (8.70%)
Other	3 (13.04%)
**Level of Intervention**^**b**^	
Individual: knowledge change	22 (95.65%)
Individual: behaviour change	15 (65.22%)
Organizational Change	4 (17.39%)
**Intervention Delivery**^**b**^	
In-person training	19 (82.61%)
Passive Campaign	5 (21.74%)
Computer/online training	3 (13.04%)
NR	1 (4.35%)
**Outcome Category**^**b**^	
Knowledge of or Attitudes to Workplace Bullying/Incivility	17 (73.91%)
Results of Incivil Behaviour and Outcomes of Workplace Bullying	14 (60.87%)
Behaviours Related to Workplace Bullying/Incivility	6 (26.09%)
Skills to Cope With Workplace Bullying/Incivility	2 (8.70%)

^a^The 4 studies with active comparators also had control/no intervention arms so the totals exceed 100%

^b^Each study can occupy one or more category so totals exceed 100

### Intervention characteristics

Most of the studies targeted the individual rather than the organisation. Specifically, 95.7% (n = 22) of studies focused on increasing knowledge and 65.2% (n = 15) on changing behaviour at the individual level; 4 studies (17.4%) focused on individual and organisational changes. A majority of the studies (n = 21, 91.3%) focused the intervention on increasing awareness around unprofessional behaviour (Table 2). A smaller number of studies (n = 8, 34.8%) targeted conflict resolution and assertiveness (n = 4, 17.4%). Of the 7 studies (30.4%) that targeted behaviour change, 4 focused on reducing sexual harassment [33, 51, 53, 55], 1 on reducing verbal harassment [48], 1 on enhancing communication skills [34], and 1 on increasing assertiveness and communication skills [42]. The format of the interventions varied; 82.61% (n = 19) of the studies reported using in-person education sessions, and 3% (n = 13.0%) used online or computer-based education sessions. Of the studies that reported the number of educational sessions, a single session was most common (n = 16 studies, 69.6%), while 5 studies (21.7%) reported using multiple sessions.

**Table 2:** Intervention characteristics.

**Study:** Anderson, 2006[45]			
**Group:** Intervention			
**Description:**			
"The final result was a 3-hour online training program representing three informational areas [Module A: develop and use a risk assessment profile to identify triggers to violence; Module B: Review theoretical models of aggression/violence; Module E: Examine legal and ethical issues] …Training modules and WPV [workplace violence] assessments (WPV checklist) were available online at specific periods for 30 days only and then placed offline. Because of the unique coding system, only study participants designated to receive training could access the training program."			
**Goal**	Awareness or Education Campaign	**Level**	Individual: knowledge change; Individual: behaviour change
**Targeted behaviour**	No	**Type of Delivery**	Active delivery: computer/online
**Source**	External program, external delivery	**Frequency**	Single Session (3-hour online training program)
**Group:** Control			
**Description:**			
"All study groups received identical study packets and consent forms; however, participants in the control group did not attend the informational session and were not designated to receive training."			
**Goal**	NA	**Level**	NA
**Targeted behaviour**	NA	**Type of Delivery**	NA
**Source**	NA	**Frequency**	NA
**Study:** Barak, 1994[33]			
**Group:** Intervention (single group) **Description:**			
"This workshop was designed for approximately 10 to 15 female workers, to be administered during 1 day, and to include various cognitive-modification techniques such as live modelling, video modelling, simulation games, role playing, and structured small-group discussions …The cognitive-behavioural workshop consisted of two phases [Phase 1: Development of awareness and understanding of sexual harassment in the workplace; Phase 2: Development of coping skills with sexual harassment in the workplace], each including three learning exercises. Before the first phase began, there was a brief introduction and a general description of the program for the day"			
**Goal**	Awareness or Education Campaign; Assertiveness training	**Level**	Individual: knowledge change; Individual: behaviour change
**Targeted behaviour**	Yes—sexual harassment	**Type of Delivery**	Active delivery: in-person
**Source**	External program, external delivery	**Frequency**	Single Session (1-day session offered twice, to two different groups on two separate days)
**Study:** Bingham, 2001[55]			
**Group:** Intervention **Description:**			
"Program format and content. The committee developed a 30-minute program consisting of three components: a 3-minute videotaped speech by the chancellor; a handout and oral presentation by mixed-sex, two-person teams of university staff and faculty; and a 5-minute discussion. The chancellor’s speech emphasized the university’s lack of tolerance for sexual harassment …The second component mainly defined sexual harassment and discussed the consequences of policy violations			
**Goal**	Awareness or Education Campaign	**Level**	Individual: knowledge change;
**Targeted behaviour**	Yes—sexual harassment	**Type of Delivery**	Active delivery: in-person
**Source**	Internal program, internal delivery	**Frequency**	Single Session (NR)
**Group:** Control **Description:**			
Did not receive training intervention			
**Goal**	NA	**Level**	NA
**Targeted behaviour**	NA	**Type of Delivery**	NA
**Source**	NA	**Frequency**	NA
**Study:** Ceravolo, 2012[34]			
**Group:** Intervention (single group) **Description:**			
"The 60- to 90-minute workshops were designed to enhance assertive communication skills and raise awareness about the impact of lateral violence behaviour. Emphasis in all of the workshops was placed on healthy conflict resolution and eliminating a culture of silence for nurses. Helpful acronyms as memory aides were shared and practiced to strengthen effective communication and conflict resolution …The workshops expanded the emphasis to nurses who agreed to serve as mentors or preceptors to newly hired nurses. We created a "train the trainer" workshop, training 20 staff nurses and educators on the content and presentation skills."			
**Goal**	Awareness or education campaign; Assertiveness training; Conflict resolution/mediation training	**Level**	Individual: knowledge change; Individual: behaviour change
**Targeted behaviour**	Yes—to enhance assertive communication skills	**Type of Delivery**	Active delivery: in-person
**Source**	Internal program, internal delivery	**Frequency**	Single Session (203 sessions of 60–90 minute workshops)
**Study:** Chipps, 2012[35]			
**Group:** Intervention (single group) **Description:**			
"An interventional program was developed by a RN organizational development consultant. The overall objectives of the educational program were to identify negative behaviors associated with workplace bullying, to develop an awareness of the personal and organizational implications of workplace bullying, to formulate a common vision for the unit team and for the individuals to create an environment of collegiality and collaboration, and to develop organizational and individual capacity for implementing strategies to create a collaborative environment. Study interventions …included both education and a system for supporting participant behavior change."			
**Goal**	Awareness or Education Campaign; Workplace policy/Employee code of conduct	**Level**	Individual: knowledge change; Individual: behaviour change; Organizational
**Targeted behaviour**	No	**Type of Delivery**	Active delivery: in-person
**Source**	External program, internal delivery	**Frequency**	Multiple Sessions (3-month educational program)
**Study:** Dahlby, 2014[36]			
**Group:** Intervention (single group) **Description:**			
" …an educational intervention focused on increasing awareness and teaching cognitive rehearsal to combat lateral violence. The project objectives were to determine the frequency of lateral violence among the program participants, increase awareness and knowledge of lateral violence behaviors, and teach the use of cognitively rehearsed responses as a technique to manage common lateral violence behaviors"			
**Goal**	Awareness or Education Campaign; Conflict resolution/mediation training	**Level**	Individual: knowledge change; Individual: behaviour change
**Targeted behaviour**	No	**Type of Delivery**	NR
**Source**	NR	**Frequency**	Single Session (single 1.5 hr session)
**Study:** Dompierre, 2008[46]			
**Group:** Intervention **Description:**			
"A committee for the prevention of violence (called the "school team") was set up to follow training in violence awareness …During the awareness activity, which was mandatory for all of the workers, the "school team" chose to stage the case studies and to make the teaching and non-teaching personnel work in teams"			
**Goal**	Awareness or education campaign	**Level**	Individual: knowledge change
**Targeted behaviour**	No	**Type of Delivery**	Active delivery: in-person
**Source**	External program, internal delivery	**Frequency**	Single Session (workshop delivered over two half-days)
**Group:** Control **Description:**			
Not Reported			
**Goal**	NA	**Level**	NA
**Targeted behaviour**	NA	**Type of Delivery**	NA
**Source**	NA	**Frequency**	NA
**Study:** Embree, 2013[37]			
**Group:** Intervention (single group) **Description:**			
"The intervention included didactic content of theoretical underpinnings and historical significance of NNLV [nurse-to-nurse lateral violence] and cognitive rehearsal. Handouts for dealing with confrontation and conflict, cue cards of NNLV responses, and expected behaviors of professionals supplemented the didactic education. Teaching strategies aimed to increase knowledge, self-esteem, and comfort with information and techniques …During the cognitive rehearsal session, nurses identified personal experiences with NNLV and offered suggestions to other participants about mechanisms they had found successful in handling NNLV"			
**Goal**	Awareness or Education Campaign; Conflict resolution/mediation training	**Level**	Individual: knowledge change; Individual: behaviour change
**Targeted behaviour**	No	**Type of Delivery**	Active delivery: in-person
**Source**	Internal program, internal delivery	**Frequency**	Single Session (Two-hour focus sessions were provided to interested nurses)
**Study:** Frisbie, 2002[53]			
**Group:** Intervention—Online Training **Description:**			
"Those assigned to the online training group were instructed to meet in the organization’s training facility …Each participant completed the online training module in the training room and was directed to the assessment via a computer link at the end of the training. Total training and assessment time ranged from 50 to 90 minutes."			
**Goal**	Awareness or Education Campaign	**Level**	Individual: knowledge change;
**Targeted behaviour**	Yes—Sexual harassment	**Type of Delivery**	Active delivery: computer/online
**Source**	External program, internal delivery	**Frequency**	Single Session (50–90 minutes (including assessment after training completed))
**Group:** Intervention—Classroom Training **Description:**			
"The live training group was also scheduled for training in the organization’s training room. These participants were presented with the same material as the online training group, however the material was presented via a power point presentation by a male trainer who was blind to the hypotheses of the experiment …The presentation was videotaped to ensure that the material presented was congruent with the online material. Upon completion of the training, the participants were asked to sign an informed consent form and to select a personal password to utilize in the online assessment. They were then directed to the online assessment site."			
**Goal**	Awareness or Education Campaign	**Level**	Individual: knowledge change;
**Targeted behaviour**	Yes—Sexual harassment	**Type of Delivery**	Active delivery: in-person
**Source**	External program, internal delivery	**Frequency**	Single Session (30 minutes for training only, does not include assessment time)
**Group:** Control **Description:**			
"The participants who were assigned to the control group were also assessed in the organization’s training room. They were presented with the informed consent form upon arrival, asked to select a personal password, and directed to the online assessment site."			
**Goal**	NA	**Level**	NA
**Targeted behaviour**	NA	**Type of Delivery**	NA
**Source**	NA	**Frequency**	NA
**Study:** Goldberg, 2007[51]			
**Group:** Intervention **Description:**			
"The training was provided in a live lecture format with discussion …The first element consisted of an overview of the relevant legislation and key court decisions regarding sexual harassment. The second identified fundamental sexual harassment terminology (e.g., quid pro quo, hostile environment, and the reasonable victim). The third focused on organizational implications related to sexual harassment (e.g., employer liability, sexual harassment policies, and grievance mechanisms). The final element of the training focused on victim responses to sexual harassment and the ramifications associated with them (e.g., formally reporting an incident may stop the unwanted behavior, but may also lead to various forms of retaliation)."			
**Goal**	Awareness or Education Campaign	**Level**	Individual: knowledge change; Individual: behaviour change
**Targeted behaviour**	Yes—sexual harassment	**Type of Delivery**	Active delivery: in-person
**Source**	External program, external delivery	**Frequency**	Single Session (2-hour training on sexual harassement)
**Group:** Control **Description:**			
Did not receive training intervention			
**Goal**	NA	**Level**	NA
**Targeted behaviour**	NA	**Type of Delivery**	NA
**Source**	NA	**Frequency**	NA
**Study:** Hoel, 2006[52]			
**Group:** Intervention—Policy Communication **Description:**			
"[participants] were invited to a 30-minute policy communication session …the aims of the policy communication intervention include raising awareness of the organisation’s policy on bullying and the duty of organisational members in its implementation…the training includes: A statement of intent from senior managers highlighting the fact that such behaviour will not be tolerated; Outline of the managers / supervisors responsibility with regard to the implementation of the policy and responsibility for challenging bullying behaviour; A definition of bullying and examples of bullying behaviour; An overview of the complaints / grievance procedure and details of key contact persons"			
**Goal**	Awareness or Education Campaign	**Level**	Individual: knowledge change; Individual: behaviour change
**Targeted behaviour**	No	**Type of Delivery**	Active delivery: in-person
**Source**	Internal program, internal delivery	**Frequency**	Single Session (30-minute policy communication session)
**Group:** Intervention—Policy Communication + Stress Management Training **Description:**			
"[participants] were involved in a 3- hour stress management training programme in addition to the policy communication…The aims of the stress management training include raising awareness of stress and its impact on individuals and the organisation, and developing manager/supervisor understanding of how to manage their stress as well as the stress of people they are responsible for. The training includes: Defining stress; Causes and consequences of stress; Differing responses to stress; How to identify stress in self and others; Coping with stress (self)–problem focused coping, time management, task prioritisation and exercise; Managing stress in others."			
**Goal**	Awareness or Education Campaign; Conflict resolution/mediation training;	**Level**	Individual: knowledge change; Individual: behaviour change
**Targeted behaviour**	No	**Type of Delivery**	Active delivery: in-person
**Source**	Internal program, internal delivery	**Frequency**	Single Session (3- hour stress management training programme in addition to the policy communication)
**Group:** Intervention—Policy Communication + Negative Behaviour Awareness Training **Description:**			
"[participants] were invited to a 3-hour negative behaviour training session in addition to the policy communication session …Using feedback obtained from focus groups and risk assessment exercises, the aims of the negative behaviour awareness training include raising awareness of negative behaviour and its impact on individuals and the organisation, and developing a shared understanding of what acceptable / unacceptable behaviour is within the organisation. The training includes: Individual experiences of negative behaviour; Definition of bullying and categories of bullying behaviour; Evidence from previous research including effects on individual and organisation; Situations that cause bullying behaviour (organisation-specific evidence from focus groups); Transactional analysis: how to develop skills for positive interaction; A statement of intent from senior managers highlighting the fact that such behaviour will not be tolerated; Outline of the managers / supervisors responsibility to challenge bullying behaviour."			
**Goal**	Awareness or Education Campaign; Conflict resolution/mediation training;	**Level**	Individual: knowledge change; Individual: behaviour change
**Targeted behaviour**	No	**Type of Delivery**	Active delivery: in-person
**Source**	Internal program, internal delivery	**Frequency**	Single Session (3-hour negative behaviour training session in addition to the policy communication session)
**Group:** Intervention—Policy Communication + Stress Management Training + Negative Behaviour Awareness Training **Description:**			
"[participants] were involved in a full day training session covering policy communication, stress management and negative behaviour awareness"			
**Goal**	Awareness or Education Campaign; Conflict resolution/mediation training;	**Level**	Individual: knowledge change; Individual: behaviour change
**Targeted behaviour**	No	**Type of Delivery**	Active delivery: in-person
**Source**	Internal program, internal delivery	**Frequency**	Single Session (full day training session covering policy communication, stress management and negative behaviour awareness)
**Group:** Control **Description:**			
"…did not take part in any intervention…"			
**Goal**	NA	**Level**	NA
**Targeted behaviour**	NA	**Type of Delivery**	NA
**Source**	NA	**Frequency**	NA
**Study:** Hultman, 2012[38]			
**Group:** Intervention (single group) **Description:**			
"We divided the course, which was entitled "Achieving Professionalism in Surgery and Anesthesia: The Journey of Becoming a Physician," into the following four segments: (1) Thinking Like a Professional: Cognitive and Ethical Components of Professionalism; (2) Acting Like a Professional: Behavioral and Social Components of Professionalism; (3) Functioning Like a Professional: Leading, Teaching, Caring; and (4) Becoming a Professional: Managing Your Self, Your Team, and Your Patients. One of four different instructors led each segment, employing many different teaching styles: lecture, small group discussion, case studies, video vignettes, journal club, and book club."			
**Goal**	Awareness or Education Campaign	**Level**	Individual: knowledge change; Individual: behaviour change
**Targeted behaviour**	No	**Type of Delivery**	Active delivery: in-person
**Source**	Internal program, internal delivery	**Frequency**	Multiple Sessions (Four 1-hour sessions spread out over 1 month)
**Study:** Keashly, 2009[47]			
**Group:** Intervention **Description:**			
" …we also educated individuals about the nature of workplace aggression and bullying in an effort to sensitize them to these issues and increase awareness of their own assumptions and behaviors. We did this through formal training sessions with the Action Teams. Once trained, the Action Teams, in turn, passed this knowledge along in formal presentations to employees and managers in each of the pilot facilities …"			
**Goal**	Awareness or Education Campaign; Conflict resolution/mediation training	**Level**	Individual: knowledge change; Individual: behaviour change
**Targeted behaviour**	No	**Type of Delivery**	Active delivery: in-person
**Source**	External program, internal delivery	**Frequency**	Not reported (NR)
**Group:** Control **Description:**			
Not Reported			
**Goal**	NA	**Level**	NA
**Targeted behaviour**	NA	**Type of Delivery**	NA
**Source**	NA	**Frequency**	NA
**Study:** Kennedy, 2010[39]			
**Group:** Intervention (single group) **Description:**			
"Directed educational Powerpoint about workplace bullying"			
**Goal**	Awareness or Education Campaign	**Level**	Individual: knowledge change;
**Targeted behaviour**	No	**Type of Delivery**	Active delivery: in-person
**Source**	External program, external delivery	**Frequency**	Single Session (NR but appears to be single session)
**Study:** Lansbury, 2014[48]			
**Group:** Intervention—Poster Campaign **Description:**			
"The group would agree to display posters in prominent locations at the requested time …A decision was made to feed-back specific information from the survey to the employees in the form of posters displayed at their place of work. The information was obtained from the first survey and was only be fed back to the group who generated it …Initially four facts were to be displayed but ultimately the survey only had space for one item for this condition (posters; figure 6.1, p. 202). The statement was: Other people think verbal bullying is unacceptable."			
**Goal**	Awareness or Education Campaign	**Level**	Individual: knowledge change;
**Targeted behaviour**	Yes—verbal bullying	**Type of Delivery**	Passive Delivery^a^
**Source**	External program, internal delivery	**Frequency**	NR
**Group:** Intervention—Training Programme **Description:**			
"The group would schedule on-site training sessions with the researcher, enabling the entire group to be trained at a specific time …The training was to inform, explain or reinforce the 15 points contained in the statements of the Responsible Bystander Intervention in Verbal Bullying (RBI-VB) metric …A presentation and practice session was developed to enable bystanders to appropriately intervene in workplace verbal bullying …The steps necessary to intervene were introduced to strengthen each statement of the RBI-VB. Through indirect reinforcement (the RBI-VB statements were not listed or read out) the concept of bystander intervention was explained."			
**Goal**	Awareness or Education Campaign	**Level**	Individual: behaviour change
**Targeted behaviour**	Yes—verbal bullying	**Type of Delivery**	Active delivery: in-person
**Source**	External program, external delivery	**Frequency**	Single Session
**Group:** Intervention—In-house campaign **Description:**			
"The group must complete the in-house campaign… …The focus of this existing in-house campaign was to raise awareness that verbal bullying causes harm and should be stopped, " …it's everyone's responsibility to put a stop to unacceptable behaviour and language" (CiC, 2011). The campaign included a call for bystander intervention. Campaign packs were distributed to a number of locations but further details of dissemination or outcome were not shared with the researcher. Packs contained a short DVD dramatising the impact of verbal bullying, a booklet to guide managers; and posters with a call to action and a helpline telephone number. The campaign was presented during small team sessions"			
**Goal**	Awareness or Education Campaign	**Level**	Individual: knowledge change;
**Targeted behaviour**	Yes—verbal bullying	**Type of Delivery**	Passive Delivery^a^
**Source**	Internal program, internal delivery	**Frequency**	NR
**Group:** Intervention—Poster + In-house campaign **Description:**			
"The group would agree to display posters in prominent locations at the requested time [and] complete the in-house campaign."			
**Goal**	Awareness or Education Campaign	**Level**	Individual: knowledge change;
**Targeted behaviour**	Yes—verbal bullying	**Type of Delivery**	Passive Delivery^a^
**Source**	Other (external program, internal delivery plus internal program and internal delivery)	**Frequency**	NR
**Group:** Intervention—Training Programme + In-house campaign **Description:**			
"The group would schedule on-site training sessions with the researcher, enabling the entire group to be trained at a specific time [and] complete the in-house campaign…"			
**Goal**	Awareness or Education Campaign	**Level**	Individual: behaviour change
**Targeted behaviour**	Yes—verbal bullying	**Type of Delivery**	Active delivery: in-person; Passive Delivery^a^
**Source**	Other (external program, external delivery + internal program and internal delivery)	**Frequency**	Single Session
**Group:** Control **Description:**			
No Intervention			
**Goal**	NA	**Level**	NA
**Targeted behaviour**	NA	**Type of Delivery**	NA
**Source**	NA	**Frequency**	NA
**Study:** Leiter, 2011[54]			
**Group:** Intervention **Description:**			
"Osatuke et al.'s (2009) CREW [Civility, Engagement, Respect in the Workforce] process was used. CREW is a process designed to enhance civility among colleagues within the USA VHA. Employees met with coworkers on their units on a weekly or biweekly basis to work on effective interpersonal interaction at work. Trained facilitators assist these groups by providing guidance on the basis of their expertise in group facilitation and knowledge of effective work group communication."			
**Goal**	Awareness or Education Campaign; Other (community building)	**Level**	Individual: knowledge change; Individual: behaviour change
**Targeted behaviour**	No	**Type of Delivery**	Active delivery: in-person
**Source**	External program, internal delivery	**Frequency**	Multiple Sessions with follow-up: 6 months of weekly CREW meetings, refresher training at 3-month point, and sustainability training at the 6-month point
**Group:** Control **Description:**			
No Intervention			
**Goal**	NA	**Level**	NA
**Targeted behaviour**	NA	**Type of Delivery**	NA
**Source**	NA	**Frequency**	NA
**Study:** Leon-Perez, 2012[44]			
**Group:** Intervention (single group) **Description:**			
"The intervention involved conflict management training. The training looked at the different types of conflict and explored ways in which conflict could be handled, including the use of strategies to manage emotions in conflict situations and the use of effective communication. The trainer used role-playing situations and group dynamics as well as constructive discussion to encourage experiential learning."			
**Goal**	Conflict resolution/mediation training	**Level**	Individual: knowledge change; Individual: behaviour change
**Targeted behaviour**	No	**Type of Delivery**	Active delivery: in-person
**Source**	External program, external delivery	**Frequency**	Multiple Sessions with follow-up: 3 sessions, each lasting 4 hours and a follow-up session at 2 weeks
**Study:** Mallette, 2011[49]			
**Group:** Intervention–Workbook **Description:**			
"Group 1: completed a workbook on horizontal violence…The workbook and self-directed e-learning module included identical content, such as the hospital's code of conduct, definitions of horizontal violence, characteristic behaviours associated with horizontal violence, the impact of horizontal violence, strategies for resolving horizontal violence and five case scenarios. Each scenario involved nurses in a horizontal violence situation. The participants reviewed three possible responses for each scenario and chose the one they believed to be the most appropriate."			
**Goal**	Awareness or Education Campaign	**Level**	Individual: knowledge change;
**Targeted behaviour**	No	**Type of Delivery**	Passive delivery^a^
**Source**	Internal program, internal delivery	**Frequency**	Single Session (most participants completed in less than 20 minutes)
**Group:** Intervention—Online E-learning **Description:**			
"Group 2: completed a self-directed e-learning module on horizontal violence…The workbook and self-directed e-learning module included identical content, such as the hospital's code of conduct, definitions of horizontal violence, characteristic behaviours associated with horizontal violence, the impact of horizontal violence, strategies for resolving horizontal violence and five case scenarios. Each scenario involved nurses in a horizontal violence situation …The self-directed e-learning module was web-based and interactive using digital characters to play out the same scenarios as in the workbook."			
**Goal**	Awareness or Education Campaign	**Level**	Individual: knowledge change;
**Targeted behaviour**	No	**Type of Delivery**	Active delivery: computer/online
**Source**	Internal program, internal delivery	**Frequency**	Single Session
**Group:** Intervention -Virtual Role-play **Description:**			
"Group 3: participated in role-play, practice and feedback within the virtual world only…The 3-D interactive virtual patient unit built on the Second Life platform matched the appearance of inpatient units within the hospital…This interactive environment was used by participants and facilitators to role-play and practise scenarios related to horizontal violence …The four research-based scenarios use for role-play and practice in the virtual patient unit were developed by a subject matter expert; each situation was based on a horizontal violence situation reported in a tertiary care hospital in Toronto, Ontario (Ridley 2011) …From the list of four scenarios, participants were asked to select the two most personally relevant scenarios for their role-play and practice sessions in the virtual patient unit."			
**Goal**	Assertiveness training; Conflict resolution/mediation training	**Level**	Individual: behaviour change
**Targeted behaviour**	No	**Type of Delivery**	Active delivery: computer/online
**Source**	Internal program, internal delivery	**Frequency**	Single Session
**Group:** Intervention—E-learning + Virtual Role-play **Description:**			
"Group 4: completed a self-directed e-learning module on horizontal violence followed by role-play, practice and feedback within the virtual world"			
**Goal**	Awareness or Education Campaign; Assertiveness training; Conflict resolution/mediation training	**Level**	Individual: knowledge change; Individual: behaviour change
**Targeted behaviour**	No	**Type of Delivery**	Active delivery: computer/online
**Source**	Internal program, internal delivery	**Frequency**	Single Session
**Group:** Control **Description:**			
"participated in no educational intervention"			
**Goal**	NA	**Level**	NA
**Targeted behaviour**	NA	**Type of Delivery**	NA
**Source**	NA	**Frequency**	NA
**Study:** Meloni, 2011[40]			
**Group:** Intervention (single group) **Description:**			
"In 2006–07, a total of 52 employees attended comprehensive training and became part of the now extensive WEO [workplace equity officer] network. The WEOs’ role was widely advertised and employees were educated about contacting a WEO if they experienced any bullying or harassment …In early 2007 a series of posters were designed by the OD Unit in consultation with the Working Group and placed in every work area to enhance awareness of bullying and harassment issues …In late 2007 a section on bullying and harassment was included in the formal, compulsory Orientation Program and Manual outlining CHCA’s position, and the support mechanisms available."			
**Goal**	Awareness or education campaign; Reporting systems; Workplace policy/Employee code of conduct	**Level**	Individual: knowledge change; Organizational
**Targeted behaviour**	No	**Type of Delivery**	Passive Delivery^a^
**Source**	Internal program, internal delivery	**Frequency**	NA
**Study:** Osatuke, 2009[50]			
**Group:** Intervention Group—CREW Group 1 **Description:**			
"[The VHA National Center for Organization Development] trains local CREW [Civility, Respect, Engagement in the Workforce] leaders from each site by explaining the rationale for CREW and its operational background and sharing the data in support of the organizational relevance of civility. A preintervention survey is conducted using the civility scale. Based on pre-intervention survey scores and/or the tools within the educational kit that they see as fitting their needs, each site chooses specific areas of foci related to civility …CREW coordinators lead interventions at their facilities by facilitating regular on-site meetings. At the meetings, baseline data on civility are first shared and discussed within workgroups. The workgroups then decide which actions to take to improve their overall civility, thus developing their own methods for improving their work environment. Six-month follow-up assessment is conducted using the same civility scale."			
**Goal**	Awareness or Education Campaign	**Level**	Individual: knowledge change;
**Targeted behaviour**	No	**Type of Delivery**	Active delivery: in-person
**Source**	External program, external delivery	**Frequency**	Multiple Sessions (regular (weekly) workgroup-level conversations)
**Group:** Intervention—CREW Group 2 **Description:**			
"[The VHA National Center for Organization Development] trains local CREW [Civility, Respect, Engagement in the Workforce] leaders from each site by explaining the rationale for CREW and its operational background and sharing the data in support of the organizational relevance of civility. A preintervention survey is conducted using the civility scale. Based on pre-intervention survey scores and/or the tools within the educational kit that they see as fitting their needs, each site chooses specific areas of foci related to civility …CREW coordinators lead interventions at their facilities by facilitating regular on-site meetings. At the meetings, baseline data on civility are first shared and discussed within workgroups. The workgroups then decide which actions to take to improve their overall civility, thus developing their own methods for improving their work environment. Six-month follow-up assessment is conducted using the same civility scale."			
**Goal**	Awareness or Education Campaign	**Level**	Individual: knowledge change;
**Targeted behaviour**	No	**Type of Delivery**	Active delivery: in-person
**Source**	External program, external delivery	**Frequency**	Multiple Sessions (regular (weekly) workgroup-level conversations)
**Group:** Control (matched to CREW Group 1) **Description:**			
Control sites that did not participate in CREW			
**Goal**	NA	**Level**	NA
**Targeted behaviour**	NA	**Type of Delivery**	NA
**Source**	NA	**Frequency**	NA
**Group:** Control (matched to CREW Group 2) **Description:**			
Control sites that did not participate in CREW			
**Goal**	NA	**Level**	NA
**Targeted behaviour**	NA	**Type of Delivery**	NA
**Source**	NA	**Frequency**	NA
**Study:** Pate, 2010[41]			
**Group:** Intervention (single group) **Description:**			
"The policy was titled Dignity at Work. There was a two-prong approach to solving the problem. The first was to pursue most robustly reported incidents of bullying …The second element was to conduct compulsory training programmes for all employees that underlined the organisation’s code of behaviour."			
**Goal**	Awareness or Education Campaign; Reporting systems; Workplace policy/Employee code of conduct; Other (pursue most robustly reported incidents of bullying)	**Level**	Individual: knowledge change; Organizational
**Targeted behaviour**	No	**Type of Delivery**	Active delivery: in-person; Passive Delivery^a^
**Source**	Internal program, internal delivery	**Frequency**	Single Session (NR)
**Sanderson, 2014[42]**			
**Group:** Intervention (single group) **Description:**			
"During November and December, 2011 the nurse leader provided service-wide education about civility and communication in the workplace and the importance of assertiveness in effective communication. As part of this educational training, printed materials containing examples of bullying behaviors and effective responses was distributed, read aloud by the staff members, and used as a guide during facilitated discussions. Staff shared pre-selected, de-identified examples of stressful events that occurred in their workplaces. Various response options were offered and discussed among participants. Nurse leader-facilitated role-play strategies were used to work through and reinforce the most effective assertive responses …During January and February, 2012 the nurse leader prepared a brochure for staff as a reinforcement of the earlier learning."			
**Goal**	Awareness or Education Campaign; Assertiveness training; Conflict resolution/mediation training	**Level**	Individual: knowledge change; Organizational
**Targeted behaviour**	Yes—assertive and effective communication	**Type of Delivery**	Active delivery: in-person; Passive Delivery^a^
**Source**	Internal program, internal delivery	**Frequency**	NA
**Stagg, 2011[43]**			
**Group:** Intervention (single group) **Description**			
"The training provided information on workplace bullying, responses to common bullying behaviors, and the cognitive rehearsal technique, along with application of the technique to common bullying behaviors."			
**Goal**	Awareness or Education Campaign; Assertiveness training; Other (cognitive rehearsal technique)	**Level**	Individual: knowledge change; Individual: behaviour change
**Targeted behaviour**	No	**Type of Delivery**	Active delivery: in-person
**Source**	Internal program, internal delivery	**Frequency**	Single Session (NR)

^a^Passive delivery was defined as any method that did not require interaction between participants and the intervention where the information could be passively consumed (e.g., posters, brochures, watching a DVD)

### Outcomes

Seventeen studies (73.9%) reported changes in knowledge of or attitudes towards unprofessional behaviour (Table 1, S5 Table). Fourteen studies (60.9%) reported on the results of incivil behaviour and outcomes of workplace bullying, 2 studies (8.7%) reported on changes in skills to cope with workplace bullying, and 6 studies (26.1%) reported on changes in behaviours related to unprofessional behaviour. Two studies [34, 37] reported on staff retention and both showed increases post-intervention. One of 2 studies that reported absenteeism [52, 54] showed a decrease following the intervention. Of 7 studies that considered perceptions or reports of bullying or harassment, 3 showed increases and 4 showed decreases following the intervention. Four studies [43–45, 52] reported use of a validated outcome measure (S6 Table).

### Qualitative synthesis

Fifty-seven items were generated from the abstracted definitions of unprofessional behaviour and grouped into four constructs: verbal or non-verbal acts (e.g., vicious words, threats, sexual assault, beating), repeated acts (e.g., persistent negative acts over a period of time), power imbalance (e.g., there is power imbalance between the perpetrator(s) and the victim(s); the victim is unable to defend themselves) and unwelcome behaviour (e.g., unwanted sexual advances, social acts of disrespect or devaluation, gossip, putdowns, sabotaging, blaming, offensive jokes, or coercion). The coding and agreement was found to be good for the qualitative analysis (Kappa coefficients ≥ 0.6). None of the constructs were included in all definitions of unprofessionalism reported in the included manuscripts (Table 3). The most comprehensive definitions for unprofessionalism were those provided in the studies by Kennedy and colleagues [39] and by Lansbury and colleagues [48].

**Table 3:** Definitions abstracted to incivility constructs.

Reference	Definition	Verbal and/or Non-verbal acts	Repeated acts	Power Imbalance	Unwelcome Behaviour	Number of constructs
**Anderson et al. (2006)[45]**	Workplace Violence: In studies on health care workers, definitions of WPV range from subjective feelings of being threatened to physical assault that results in injury or death. For each death from WPV, there are countless other incidents of WPV in which the victim is harassed or threatened. McPhaul and Lipscomb (2004) defined violence as a range of behaviors from verbal abuse, threats, and unwanted sexual advances to physical assault and homicide. Sexual harassment is also considered within this definition. In most instances (62%), the perpetrator has no weapon (Lynch, 1987), with nearly half of all WPV events reported including hitting, kicking, pinching, scratching, stabbing, shooting, rape, threats, and beating.	X	X		X	3
**Barak et al. (1994) [33]**	sexual harassment				X	1
**Bingham et al. (2001)[55]**	Sexual harassment				X	1
**Ceravolo et al. (2012)[34]**	Inclusive of bullying, incivility and social acts of disrespect, lateral violence is a nurse-to-nurse social devaluation or control of a peer through overt and covert verbal, physical and emotional abuse (Embree & White 2010).	X		X	X	3
**Chipps et al. (2012)[35]**	1. Workplace bullying has been defined ‘‘as repeated and persistent negative acts toward one or more individuals, which involve a perceived power imbalance and create a hostile work environment” (Salin, 2003, p. 1214). It is important to note that workplace bullying reflects intentional and ongoing negative acts, which accumulate over time. Bullying has been described as having four distinct features: intensity, repetition, duration, and power disparity. Intensity describes the number of negative acts experienced by the target. Repetition suggests that the negative act is not a one time isolated event or interaction. Duration reflects that the negative act occurs over a designated time. Lastly, power disparity suggests that the target of bullying is unable to eradicate the abuse (Lutgen- Sandvik, Tracy, & Alberts, 2007). ‘‘We define bullying as a situation where one or several individuals perceive themselves to be on the receiving end of negative actions from one or more persons persistently over a period of time, in a situation where the targets have difficulty defending themselves against these actions. We do not refer to a one-time incident as bullying.” This has been described as self-identified bullying (Lutgen-Sandvik et al., 2007).; 2. workplace bullying is broader in conceptual scope than the more commonly used term ‘‘horizontal hostility,” which has been used in the nursing literature. Horizontal hostility implies negative behaviors directed at the peer or coworker level (Alspach, 2007; Bartholomew, 2006; Johnson & Rea, 2009). In contrast, the term ‘‘workplace bullying” does not define the hierarchical structure of the relationship. Thus, the concept of workplace bullying can be used to encompass the entire team of healthcare providers and crosses the hierarchical structure of the nursing work environment. Workplace bullying has been defined ‘‘as repeated and persistent negative acts toward one or more individuals, which involve a perceived power imbalance and create a hostile work environment” (Salin, 2003, p. 1214).		X	X		2
**Dahlby et al. (2014)[36]**	Lateral violence is described as disruptive, disparaging, or uncivil behavior inflicted by one peer on another (Dimarino, 2011). The result is an unpleasant work environment that has harmful effects on individual nurses, team members, and patients, and also has financial implications for the organization. Lateral violence is defined legally as occurring "when oppressed groups/individuals internalize feelings such as anger and rage, and manifest their feelings through behaviors such as gossip, jealousy, putdowns, and blaming" (Dimarino, 2011, p. 583). Acts of lateral violence most commonly include verbally or nonverbally insulting a coworker, gossiping, undermining, withholding information, sabotaging, infighting, scapegoating, backstabbing, failing to respect privacy, and breaking confidences.	X			X	2
**Dompierre et al. (2008)[46]**	In this study, the definition of violence in the workplace adopted by the European Commission is used: "Any event during which persons are victims of abusive behaviors, of threats or of attacks in circumstances linked to their job and implying an explicit or implicit risk for their security, their well-being and their health (Wynne et al., 1997)".				X	1
**Embree et al. (2013)[37]**	Nurse-to-nurse lateral violence occurs when oppressed groups or individuals internalize feelings such as anger and rage and display these emotions through behaviors such as gossiping, exhibiting jealousy, putting others down, and blaming others for their actions [13] […]Lateral violence (LV) is described as behavior demonstrated by nurses who overtly or covertly direct dissatisfaction toward those less powerful than themselves and each other [12]. Manifested verbally and nonverbally, the ten most common universal forms of LV in nursing are "nonverbal innuendo," "verbal affront," "undermining activities," "withholding information," "sabotage," "infighting," "scapegoating," "backstabbing," "failure to respect privacy," and "broken confidences" [12].	X		X	X	3
**Frisbie et al. (2002)[53]**	Sexual harassment: A form of sex discrimination which occurs when "unwelcome verbal or physical conduct of a sexual nature…affects an individual’s employment, unreasonably interferes with an individual’s work performance or creates an intimidating, hostile or offensive work environment" (U.S. Equal Employment Opportunity Commission, 1980, p. 74677).	X			X	2
**Goldberg et al. (2007)[51]**	Sexual harassment has been defined as "unwelcome sexual advances, requests for sexual favors, and other verbal or physical conduct of a sexual nature" (U.S. Equal Employment Opportunity Commission, 1990).	X			X	2
**Hoel et al. (2006)[52]**	We define bullying as a situation where one or several individuals persistently over a period of time perceive themselves to be on the receiving end of negative actions from one or several persons, in a situation where the target of bullying has difficulty in defending him or herself against these actions. We will not refer to a one-off incident as bullying.		X	X		2
**Hultman et al. (2012)[38]**	Medical professionalism					
**Keashly et al. (2009)[47]**	Workplace aggression and bullying					
**Kennedy et al. (2010)[39]**	Workplace bullying—the persistent demeaning and downgrading of humans through vicious words in negative acts that gradually undermines confidence and self esteem (Strandmark et al., p. 332). Oppression–usually to subject (a people) to burdens, to undue exercise of authority, and the like; its chief application, therefore, is to a social or political situation (Dictionary.com, 2009).	X	X	X	X	4
**Lansbury et al. (2014)[48]**	"Verbal bullying is repeated, negative verbal behaviour where the target feels they can't defend themselves. This includes inappropriate behaviour such as insulting comments, excessive teasing, threats, humiliating interaction, jokes, offensive remarks about someone's private life and persistent criticism."	X	X	X	X	4
**Leiter et al. (2011)[54]**	Incivility refers to rude or discourteous behaviour that conveys disrespect toward others (Andresson & Pearson 1999). It can be differentiated from aggression, which has greater intensity and clearer intention.					
**Leon-Perez et al. (2012)[44]**	Workplace bullying is always regarded as a destructive process that causes negative outcomes, including the possibility of post- traumatic stress and suicide (Leymann, 1996; Van de Vliert, 2010).				X	1
**Mallette et al. (2011)[49]**	Horizontal violence encompasses such disrespectful behaviours as intimidation, coercion, bullying, criticism, exclusion or belittling in public or private.				X	1
**Meloni et al. (2011)[40]**	Within Calvary Health Care ACT, harassment was defined as a behaviour towards an individual or a group which is offensive, humiliating, intimidating or threatening; is unwelcome, unsolicited, usually unreciprocated, and a reasonable person would consider to be offensive, humiliating, intimidating or threatening. Bullying was defined as repeated inappropriate behaviour, direct or indirect, whether verbal, physical or otherwise, conducted by one or more persons against other(s), which may be considered unreasonable and inappropriate workplace practice.	X	X		X	3
**Pate et al. (2010)[41]**	[…] repeated and persistent negative actions towards one or more individual(s), which involves a perceive power imbalance and create a hostile environment (Sandvik et al., 2007, p. 838).		X	X		2
**Stagg et al. (2011)[43]**	Workplace bullying is defined as repetitive inappropriate behavior, direct or indirect, whether verbal, physical, or otherwise, carried out by one or more persons against another or others, at the workplace and/ or in the course of employment, which undermines the individual’s right to dignity at work (Center for American Nurses, 2008).	X	X			2
**Number of definitions**		10 [48%]	8[38%]	7[33%]	14[67%]	

## Discussion

We identified 23 studies that described interventions for preventing or managing unprofessional behaviours in a variety of settings. Most of the studies included single component in-person education sessions that targeted individuals, while fewer studies targeted both individuals and institutional culture and most focused on increasing awareness of unprofessional behaviour rather than effecting behaviour change. In studies that assessed the impact of interventions on outcomes such as reports or perceptions of unprofessional behaviour, results were mixed with some showing increases and some decreases following the intervention. These mixed results may be due to increased awareness of the unprofessional behaviour, leading to more comfort in reporting it. Overall, educational interventions may work but they need to be tailored to individual and organisational needs.

The culture of unprofessionalism in academic medicine may be perpetuated through the modelling of abuse that starts in training, thereby normalising the behaviour [19, 56–59]. Our scoping review identified few studies that have targeted academic centres to mitigate this culture. While studies from other fields such as private industry and government may be useful, there are unique aspects of the academic medicine setting that need to be considered when contextualising these interventions. For example, professional hierarchies within medical specialists, the high stress medical environment that includes long work hours and on-call responsibilities, and the physician shortage in certain settings can all exacerbate the risk of unprofessional behaviour [14, 60–62]. Concerns about retaliation may also prohibit reporting of unprofessional behaviour, therefore allowing the behaviour to continue [14, 60–62].

Our scoping review identified a lack of consistency in definitions and terms used for unprofessional behaviour (S1 Fig). Unprofessionalism can include a wide range of behaviours that people perceive as hostile, abusive or humiliating [4]. The lack of agreement on the definitions, terms and behaviours make measuring the behaviour challenging. Thus measuring the impact of strategies to promote professionalism is problematic. Several studies have highlighted the challenge of assessing professionalism and the critical need for further work in this area [63–67].

There are limitations to our scoping review that should be considered. First, our literature search was challenging because of the lack of agreement on the definition and terms used for unprofessional behaviour. As such, our search was broad with >12,000 citations retrieved but we may have missed relevant articles. However, we contacted experts in the field and reviewed references of relevant systematic reviews to facilitate saturation. Second, because this was a scoping review, we did not conduct a risk of bias assessment of the included studies; this could be done in a future, full systematic review.

We believe that the results of this scoping review can be used to target a full systematic review. Given that several relevant studies were identified, this systematic review could focus on studies conducted in health care organisations or educational settings. We also suggest that a realist review could be undertaken that would include the qualitative literature; this would be particularly helpful because of the need to contextualise the effectiveness of interventions. Specifically, a realist review would inform which circumstances and settings a particular intervention would work to mitigate unprofessional behaviour [68].

## Conclusions

This is the first scoping review of strategies to mitigate professional behaviour in workplace settings. It identified where a future systematic review could inform practice in academic medicine and medical education. Most of the studies included single component, in-person education sessions targeting individuals and increasing awareness of unprofessional behaviour. There is limited evidence that printed education materials, and large group education sessions substantially change physician behaviour [69, 70]. Given the need to effect individual behaviour change, strategies to increase awareness are likely not sufficient to address unprofessional behaviour and future primary studies should use behaviour change theory and evidence around what interventions work to effect behaviour change [71]. Moreover, the interventions need to target barriers to professionalism. As fewer studies targeted the institutional culture, this is a critical element to consider in future research.

## Supporting information

S1 Table PRISMA checklist. (PDF)

S2 Table Workplace bullying definitions, behaviours, and key words. (PDF)

S3 Table Individual study characteristics. (PDF)

S4 Table Individual participant characteristics. (PDF)

S5 Table Individual study outcomes. (PDF)

S6 Table Measurement tools and scales. (PDF)

S1 Fig Word cloud of terms used in definitions of Workplace bullying/incivility. (PDF)

S1 Text MEDLINE search strategy. (PDF)

S2 Text List of sites searched for grey literature. (PDF)

## References

[1] Bazelon E. Bullying in the Age of Trump The New York Times 2016. Available from: https://www.nytimes.com/2016/11/16/opinion/bullying-in-the-age-of-trump.html?_r=1.

[2] Boyle T. Ontario doctors ‘distressed’ over wave of bullying, infighting: The Toronto Star; 2017. Available from: https://www.thestar.com/life/health_wellness/2017/02/27/ontario-doctors-distressed-over-wave-of-bullying-infighting.html.

[3] Farley SS, C. Culture of cruelty: why bullying thrives in higher education 2014 [cited 2017]. Available from: https://www.theguardian.com/higher-education-network/blog/2014/nov/03/why-bullying-thrives-higher-education.

[4] Fnais N, Soobiah C, Chen MH, Lillie E, Perrier L, Tashkhandi M, et al Harassment and discrimination in medical training: a systematic review and meta-analysis. Acad Med. 2014;89(5):817–27. 10.1097/ACM.0000000000000200 24667512

[5] Rouse LP, Gallagher-Garza S, Gebhard RE, Harrison SL, Wallace LS. Workplace bullying among family physicians: A gender focused study. J Womens Health. 2016;25(9):882–8. 10.1089/jwh.2015.5577 27268083

[6] Timm A. ‘It would not be tolerated in any other profession except medicine’: survey reporting on undergraduates’ exposure to bullying and harassment in their first placement year. BMJ open. 2014;4(7):e005140 10.1136/bmjopen-2014-005140 25009133 PMC4091461

[7] Miedema B, Hamilton R, Lambert-Lanning A, Tatemichi SR, Lemire F, Manca D, et al Prevalence of abusive encounters in the workplace of family physicians A minor, major, or severe problem? Can Fam Physician. 2010;56(3):e101–e8. 20228289 PMC2837705

[8] Leisy HB, Ahmad M. Altering workplace attitudes for resident education (A.W.A.R.E.): discovering solutions for medical resident bullying through literature review. BMC Med Educ. 2016;16(1):127 10.1186/s12909-016-0639-8 27117063 PMC4847214

[9] Magin PJ, Adams J, Sibbritt DW, Joy E, Ireland MC. Experiences of occupational violence in Australian urban general practice: a cross-sectional study of GPs. Med J Aust. 2005;183(7):352–6. 16201952 10.5694/j.1326-5377.2005.tb07082.x

[10] Phillips SP, Schneider MS. Sexual harassment of female doctors by patients. N Engl J Med. 1993;329(26):1936–9. 10.1056/NEJM199312233292607 8247058

[11] Gale C, Arroll B, Coverdale J. Aggressive acts by patients against general practitioners in New Zealand: one-year prevalence. N Z Med J. 2006;119(1237). 16862196

[12] Askew DA, Schluter PJ, Dick M-L, Régo PM, Turner C, Wilkinson D. Bullying in the Australian medical workforce: cross-sectional data from an Australian e-Cohort study. Aust Health Rev. 2012;36(2):197–204. 10.1071/AH11048 22624642

[13] Frank E, Carrera JS, Stratton T, Bickel J, Nora LM. Experiences of belittlement and harassment and their correlates among medical students in the United States: longitudinal survey. Bmj. 2006;333(7570):682 10.1136/bmj.38924.722037.7C 16956894 PMC1584373

[14] Miedema B, MacIntyre L, Tatemichi S, Lambert-Lanning A, Lemire F, Manca D, et al How the medical culture contributes to coworker-perpetrated harassment and abuse of family physicians. Ann Fam Med. 2012;10(2):111–7. 10.1370/afm.1341 22412002 PMC3315133

[15] Zahid M, Al-Sahlawi K, Shahid A, Awadh J, Abu-Shammah H. Violence against doctors: 2. Effects of violence on doctors working in accident and emergency departments. Eur J Emerg Med. 1999;6(4):305–9. 10646918 10.1097/00063110-199912000-00006

[16] Ahmed I, Banu H, Al-Fageer R, Al-Suwaidi R. Cognitive emotions: depression and anxiety in medical students and staff. Journal of critical care. 2009;24(3):e1–7. Epub 2009/08/12. 10.1016/j.jcrc.2009.06.003 19664516

[17] Heponiemi T, Kouvonen A, Virtanen M, Vanska J, Elovainio M. The prospective effects of workplace violence on physicians' job satisfaction and turnover intentions: the buffering effect of job control. BMC Health Serv Res. 2014;14:19 Epub 2014/01/21. 10.1186/1472-6963-14-19 24438449 PMC3898009

[18] Goldstein EA, Maestas RR, Fryer-Edwards K, Wenrich MD, Oelschlager AM, Baernstein A, et al Professionalism in medical education: an institutional challenge. Acad Med. 2006;81(10):871–6. Epub 2006/09/21. 10.1097/01.ACM.0000238199.37217.68 16985343

[19] Dorsey JK, Roberts NK, Wold B. Feedback matters: the impact of an intervention by the dean on unprofessional faculty at one medical school. Acad Med. 2014;89(7):1032–7. 10.1097/ACM.0000000000000275 24979173

[20] Farnan JM, O'Leary KJ, Didwania A, Icayan L, Saathoff M, Bellam S, et al Promoting professionalism via a video-based educational workshop for academic hospitalists and housestaff. J Hosp Med. 2013;8(7):386–9. Epub 2013/06/20. 10.1002/jhm.2056 23780912

[21] Arksey H, O'Malley L. Scoping studies: towards a methodological framework. Int J Soc Res Methodol. 2005;8(1):19–32.

[22] Peters M, Godfrey C, McInerney P, Soares C, Hanan K, Parker D. The Joanna Briggs Institute Reviewers' Manual 2015: Methodology for JBI Scoping Reviews. 2015.

[23] Rios P, Tricco AC. Academic Bullying Open Science Framework2016. Available from: https://osf.io/cajy4/.

[24] Moher D, Liberati A, Tetzlaff J, Altman DG. Preferred reporting items for systematic reviews and meta-analyses: the PRISMA statement. Bmj. 2009;339:b2535 Epub 2009/07/23. 10.1136/bmj.b2535 19622551 PMC2714657

[25] Stone PW. Popping the (PICO) question in research and evidence-based practice. Appl Nurs Res. 2002;15(3):197–8. Epub 2002/08/13. 12173172 10.1053/apnr.2002.34181

[26] Johnson SL. International perspectives on workplace bullying among nurses: a review. Int Nurs Rev. 2009;56(1):34–40. Epub 2009/02/26. 10.1111/j.1466-7657.2008.00679.x 19239514

[27] Nielsen MB, Einarsen S. Outcomes of exposure to workplace bullying: A meta-analytic review. Work & Stress. 2012;26(4):309–32.

[28] McGowan J, Sampson M, Salzwedel DM, Cogo E, Foerster V, Lefebvre C. PRESS Peer Review of Electronic Search Strategies: 2015 Guideline Statement. J Clin Epidemiol. 2016;75:40–6. Epub 2016/03/24. 10.1016/j.jclinepi.2016.01.021 27005575

[29] CADTH. Grey Matters: a practical tool for searching health-related grey literature 2015. Available from: https://www.cadth.ca/resources/finding-evidence/grey-matters.

[30] Knowledge Translation Program. Synthesi SR Toronto, Ontario: Li Ka Shing Knowledge Institute, St. Michael's Hospital; 2014 [September 2015]. Available from: http://www.breakthroughkt.ca/login.php.

[31] Tricco AC, Lillie E, Zarin W, O'Brien K, Colquhoun H, Kastner M, et al A scoping review on the conduct and reporting of scoping reviews. BMC Med Res Methodol. 2016;16:15 Epub 2016/02/10. 10.1186/s12874-016-0116-4 26857112 PMC4746911

[32] International Q. NVivo qualitative data analysis Software 10 ed Australia: QSR International Pty Ltd; 2012.

[33] Barak A. A cognitive-behavioral educational workshop to combat sexual harassment in the workplace. J Couns Dev. 1994;72(6):595–602. 10.1002/j.1556-6676.1994.tb01688.x.

[34] Ceravolo DJ, Schwartz DG, Foltz-Ramos KM, Castner J. Strengthening communication to overcome lateral violence. J Nurs Manag. 2012;20(5):599–606. 10.1111/j.1365-2834.2012.01402.x 22823215

[35] Chipps EM, McRury M. The development of an educational intervention to address workplace bullying: a pilot study. J Nurses Staff Dev. 2012;28(3):94–8. 10.1097/NND.0b013e31825514bb 22617778

[36] Dahlby MA, Herrick LM. Evaluating an educational intervention on lateral violence. J Contin Educ Nurs. 2014;45(8):344–50; quiz 51–2. 10.3928/00220124-20140724-15 25081124

[37] Embree JL, Bruner DA, White A. Raising the Level of Awareness of Nurse-to-Nurse Lateral Violence in a Critical Access Hospital. Nurs Res Pract. 2013;2013:207306 10.1155/2013/207306 23991337 PMC3748730

[38] Hultman CS, Connolly A, Halvorson EG, Rowland P, Meyers MO, Mayer DC, et al Get on your boots: preparing fourth-year medical students for a career in surgery, using a focused curriculum to teach the competency of professionalism. J Surg Res. 2012;177(2):217–23. 10.1016/j.jss.2012.06.019 22878148

[39] Kennedy M. Workplace bullying: The enculturated group behavior of nurses Southern Nazarene University.

[40] Meloni M, Austin M. Implementation and outcomes of a zero tolerance of bullying and harassment program. Aust Health Rev. 2011;35(1):92–4. 10.1071/AH10896 21367338

[41] Pate J, Beaumont P. Bullying and harassment: A case of success? Employee Relations. 2010;32(2):171–83. 10.1108/01425451011010113.

[42] Sanderson L. Improving civility in the mental health nursing workplace through assertiveness training with role-play. Dissertation Abstracts International: Section B: The Sciences and Engineering. 2014;74(11-B(E)):No Pagination Specified. PubMed PMID: Dissertation Abstract: 2014-99100-313.

[43] Stagg SJ, Sheridan D, Jones RA, Speroni KG. Evaluation of a workplace bullying cognitive rehearsal program in a hospital setting. J Contin Educ Nurs. 2011;42(9):395–401; quiz 2–3. 10.3928/00220124-20110823-45. 21877661

[44] Leon-Perez JM, Arenas A, Griggs TB. Effectiveness of conflict management training to prevent workplace bullying Workplace bullying: Symptoms and solutions. New York, NY: Routledge/Taylor & Francis Group; US; 2012 p. 230–43.

[45] Anderson C. Training efforts to reduce reports of workplace violence in a community health care facility. J Prof Nurs. 2006;22(5):289–95. 10.1016/j.profnurs.2006.07.007 16990120

[46] Dompierre J, Laliberte D, Girard S, Gignac S. A qualitative and quantitative evaluation of an experiment for preventing violence in the workplace. Eur Rev Appl Psychol. 2008;58(4):275–83. 10.1016/j.erap.2008.09.010.

[47] Keashly L, Neuman JH. Building a constructive communication climate: The Workplace Stress and Aggression Project Destructive organizational communication: Processes, consequences, and constructive ways of organizing. New York, NY: Routledge/Taylor & Francis Group; US; 2009 p. 339–62.

[48] Lansbury L. The development, measurement and implementation of a bystander intervention strategy: A field study on workplace verbal bullying in a large UK organisation: University of Portsmouth; 2014.

[49] Mallette C, Duff M, McPhee C, Pollex H, Wood A. Workbooks to virtual worlds: a pilot study comparing educational tools to foster a culture of safety and respect in Ontario. Nurs Leadersh (Tor Ont). 2011;24(4):44–64. 22273558 10.12927/cjnl.2012.22714

[50] Osatuke K, Moore SC, Ward C, Dyrenforth SR, Belton L. Civility, respect, engagement in the workforce (CREW): Nationwide organization development intervention at veterans health administration. J Appl Behav Sci. 2009;45(3):384–410. 10.1177/0021886309335067

[51] Goldberg CB. The impact of training and conflict avoidance on responses to sexual harassment. Psychol Women Q. 2007;31(1):62–72. 10.1111/j.1471-6402.2007.00331.x

[52] Hoel H, Giga SI. Destructive interpersonal conflict in the workplace: The effectiveness of management interventions. Destructive Interpersonal Conflict in the Workplace: The Effectiveness of Management Interventions. 2006.

[53] Frisbie SH. Sexual harassment: A comparison of online versus traditional training methods. Dissertation Abstracts International: Section B: The Sciences and Engineering. 2002;62(10-B):4837. PubMed PMID: Dissertation Abstract: 2002-95008-310.

[54] Leiter MP, Laschinger HKS, Day A, Oore DG. The impact of civility interventions on employee social behavior, distress, and attitudes. J Appl Psychol. 2011;96(6):1258–74. 10.1037/a0024442 21744942

[55] Bingham SG, Scherer LL. The unexpected effects of a sexual harassment educational program. J Appl Behav Sci. 2001;37(2):125–53.

[56] Cote L, Laughrea PA. Preceptors' understanding and use of role modeling to develop the CanMEDS competencies in residents. Acad Med. 2014;89(6):934–9. Epub 2014/05/30. 10.1097/ACM.0000000000000246 24871246

[57] Bryden P, Ginsburg S, Kurabi B, Ahmed N. Professing professionalism: are we our own worst enemy? Faculty members' experiences of teaching and evaluating professionalism in medical education at one school. Acad Med. 2010;85(6):1025–34. Epub 2010/01/14. 10.1097/ACM.0b013e3181ce64ae 20068427

[58] Park J, Woodrow SI, Reznick RK, Beales J, MacRae HM. Observation, reflection, and reinforcement: surgery faculty members' and residents' perceptions of how they learned professionalism. Acad Med. 2010;85(1):134–9. Epub 2010/01/01. 10.1097/ACM.0b013e3181c47b25 20042839

[59] Quaintance JL, Arnold L, Thompson GS. What students learn about professionalism from faculty stories: an "appreciative inquiry" approach. Acad Med. 2010;85(1):118–23. Epub 2010/01/01. 10.1097/ACM.0b013e3181c42acd 20042837

[60] Manca D, Varnhagen S, Brett-MacLean P, Allan GM, Szafran O. RESPECT from specialists Concerns of family physicians. Can Fam Physician. 2008;54(10):1434–5. e5. 18854474 PMC2567269

[61] Coverdill JE, Alseidi A, Borgstrom DC, Dent DL, Dumire RD, Fryer J, et al Professionalism in the Twilight Zone: A Multicenter, Mixed-Methods Study of Shift Transition Dynamics in Surgical Residencies. Acad Med. 2016;91(11 Association of American Medical Colleges Learn Serve Lead: Proceedings of the 55th Annual Research in Medical Education Sessions):S31–s6. Epub 2016/10/26. 10.1097/ACM.0000000000001358 27779507

[62] Sun NZ, Gan R, Snell L, Dolmans D. Use of a Night Float System to Comply With Resident Duty Hours Restrictions: Perceptions of Workplace Changes and Their Effects on Professionalism. Acad Med. 2016;91(3):401–8. Epub 2015/10/22. 10.1097/ACM.0000000000000949 . 26488569

[63] Young ME, Cruess SR, Cruess RL, Steinert Y. The Professionalism Assessment of Clinical Teachers (PACT): the reliability and validity of a novel tool to evaluate professional and clinical teaching behaviors. Advances in Health Sciences Education. 2014;19(1):99–113. 10.1007/s10459-013-9466-4 23754583

[64] Hodges BD, Ginsburg S, Cruess R, Cruess S, Delport R, Hafferty F, et al Assessment of professionalism: Recommendations from the Ottawa 2010 Conference. Medical teacher. 2011;33(5):354–63. 10.3109/0142159X.2011.577300 21517683

[65] Ginsburg S, Bernabeo E, Ross KM, Holmboe ES. "It depends": results of a qualitative study investigating how practicing internists approach professional dilemmas. Acad Med. 2012;87(12):1685–93. Epub 2012/10/26. 10.1097/ACM.0b013e3182736dfc . 23095932

[66] Ginsburg S, Lingard L. 'Is that normal?' Pre-clerkship students' approaches to professional dilemmas. Medical education. 2011;45(4):362–71. Epub 2011/03/16. 10.1111/j.1365-2923.2010.03903.x . 21401684

[67] Bernabeo EC, Holmboe ES, Ross K, Chesluk B, Ginsburg S. The utility of vignettes to stimulate reflection on professionalism: theory and practice. Advances in health sciences education: theory and practice. 2013;18(3):463–84. Epub 2012/06/22. 10.1007/s10459-012-9384-x . 22717991

[68] Kastner M, Antony J, Soobiah C, Straus SE, Tricco AC. Conceptual recommendations for selecting the most appropriate knowledge synthesis method to answer research questions related to complex evidence. J Clin Epidemiol. 2016;73:43–9. Epub 2016/02/26. 10.1016/j.jclinepi.2015.11.022 . 26912124

[69] Grudniewicz A, Kealy R, Rodseth RN, Hamid J, Rudoler D, Straus SE. What is the effectiveness of printed educational materials on primary care physician knowledge, behaviour, and patient outcomes: a systematic review and meta-analyses. Implementation science: IS. 2015;10:164 Epub 2015/12/03. 10.1186/s13012-015-0347-5 ; PubMed Central PMCID: PMCPMC4666153. 26626547 PMC4666153

[70] Forsetlund L, Bjorndal A, Rashidian A, Jamtvedt G, O'Brien MA, Wolf F, et al Continuing education meetings and workshops: effects on professional practice and health care outcomes. The Cochrane database of systematic reviews. 2009;(2):Cd003030 Epub 2009/04/17. 10.1002/14651858.CD003030.pub2 . 19370580 PMC7138253

[71] Straus S, Tetroe J, Graham ID. Knowledge translation in health care: moving from evidence to practice 2nd ed Oxford, UK: John Wiley & Sons; 2013.

