# Supplementary material for: Prevention and management of unprofessional behaviour among adults in the workplace: A scoping review
Source: PLoS One. 2018 Jul 26;13(7):e0201187. doi: 10.1371/journal.pone.0201187 (PMC6062077; doi:10.1371/journal.pone.0201187)
Supplement: S2 Table — (PDF) [file pone.0201187.s002.pdf]

S2 Table. Workplace bullying definitions, behaviours, and key words

**Review**	Johnson, 2009[1]

Key Words

workplace bullying ; horizontal violence; lateral violence; mobbing

Definition

There is no one agreed upon definition of workplace bullying. However, there is agreement that bullying is different from simple conflict in that it occurs more frequently, and for a longer period of time (Leymann 1996; Lutgen Sandvik et al. 2007). Also, in workplace bullying, unlike simple conflicts, the victim is unable to defend themselves and bring about an end to the conflict because they have less power than the bully (Leymann 1996; Lutgen-Sandvik et al. 2007). In general, workplace bullying occurs when the victim 'experiences at least two negative acts, weekly or more often, for six or more months in situations where targets find it difficult to defend against and stop abuse.' (Lutgen- Sandvik et al. 2007, p. 841).

Workplace bullying was initially called 'mobbing' when it was identified in Sweden in the 1980s by the psychologist Heinz Leymann (Einarsen et al. 2003). In the 1990s, researchers in the UK began to study the same phenomena, which they labeled 'bullying' (Rayner & Keashly 2005). In the USA, researchers have focused on similar workplace issues, such as emotional abuse and generalized harassment, but have only recently begun to study workplace bullying in a systematic manner consistent with research that has been done in other countries (Lutgen-Sandvik et al. 2007; Rayner & Keashly 2005). In the nursing literature, bullying is often defined as 'lateral or horizontal violence' (Curtis et al. 2007; Griffin 2004), 'verbal abuse' (Rowe & Sherlock 2005) or 'workplace aggression' (Farrell et al. 2006). This lack of a clear term and clear definition, makes it hard to compare the results of studies to each other, and to research on other occupational groups

Identified Behaviours

Behaviours that constitute bullying include:

- threats to professional status, such as belittling remarks, persistent criticism, humiliation, intimidation and inaccurate accusations (Moayed et al. 2006; Quine 2001; Zapf & Einarsen 2005)
- Bullies can threaten an individual's social status through verbal and physical threats and aggression, and by spreading rumours (Moayed et al. 2006; Quine 2001; Yildirim & Yildirim 2007).
- Social isolation, manifested through acts such as withholding information, not returning phone calls and emails, and ignoring a person (Moayed et al. 2006; Quine 2001; Zapf & Einarsen 2005).
- The bullied individual might be subjected to an unreasonable workload, unrealistic deadlines, and excessive monitoring of their work (Quine 2001; Yildirim & Yildirim 2007).
- Additionally, their professional status might be destabilized by giving them meaningless tasks, tasks that are beneath their level of competence or by removing key areas of responsibility from them (Moayed et al. 2006; Quine 2001; Yildirim & Yildirim 2007; Zapf & Einarsen 2005).

**Review**	Nielsen, 2012[2]

*Key Words**

Bullying, harassment, mobbing, emotional abuse, and victimization/victimization

*authors seem to cite (Einarsen et al., 2011b; Zapf & Einarsen, 2005)as the source for these terms

Definition

Workplace bullying is defined as a situation in which one or several individuals persistently, and over a period of time, perceive themselves as being on the

receiving end of negative actions from superiors or coworkers, and where the target of the bullying finds it difficult to defend him or herself against these actions (Einarsen & Skogstad, 1996; Olweus, 1993). That is, while many instances of interpersonal aggression take the form of individual episodes, workplace bullying is by definition characterized by systematic and prolonged exposure to repeated negative and aggressive behaviour of a primarily psychological nature, including non-behaviour and acts of social exclusion (Einarsen, Hoel, Zapf, & Cooper, 2011b; Leymann, 1996).

Identified Behaviours

Although there is no definitive list of bullying behaviour, bullying mainly involves exposure to:

- verbal hostility,
- being made the laughing stock of the department,
- having one's work situation obstructed, or
- being socially excluded from the peer group.

Empirically, such behaviour has been differentiated into seven categories:

- work related bullying,
- social isolation,
- attacking the private sphere,
- verbal aggression,
- the spreading of rumours,
- physical intimidation, and
- attacking personal attitudes and values

(Zapf, Knorz, & Kulla, 1996)

**Review**	Quinlan, 2014[3]

Key Words

''health care and bullying,''; ''health professions and bullying,'' and ''workplace bullying and health care.''

Definition

Searches for the scoping review indicate many different definitions of workplace bullying. While they all fall under the general rubric of repeated, sustained aggressive behavior within an interpersonal relationship, [26, 31–34] bullying does not have a single, universal meaning; it is assembled in multiple ways, mediated by social and material conditions. The definition congruent with the research on bullying and health care providers is as follows: workplace bullying is the systematic mistreatment of a subordinate or colleague by one or more individuals from the same group, over a frequent (at least once a week) and long period (at least six months) of time that can cause severe social, psychological, and psychosomatic problems in the victim.

Identified Behaviours

NR

**Review**	Stagg, 2010[4]

Key Words

bullying, evaluation, harassment, implementation, violence, and workplace bullying

Definition

Workplace bullying has several definitions. For the purposes of this article, workplace bullying is defined as the persistent demeaning and downgrading of individuals through vicious words and cruel acts that progressively undermine confidence and self-esteem (Adams, 1997).

Numerous interchangeable terms have been used in the literature to describe workplace bullying. Workplace bullying is commonly known as *horizontal violence* and *aggression* (Sheridan-Leos, 2008); *mobbing, horizontal hostility*, and *lateral violence* (Craig & Kupperschmidt, 2008); and *verbal abuse* (Alspach, 2007). In addition, almost every nurse has heard the phrase "eating our young," which is also used to refer to workplace bullying (Bartholomew, 2006).

*Identified Behaviours**

nonverbal innuendos, verbal affront, undermining activities, withholding information, sabotage, infighting, scapegoating, backbiting, failure to respect privacy, and broken confidences

(Griffin, 2004)

*these were behaviours identified specifically by nurses

References

1. Johnson SL. International perspectives on workplace bullying among nurses: a review. Int Nurs Rev. 2009;56(1):34-40. Epub 2009/02/26. doi: 10.1111/j.1466-7657.2008.00679.x.
2. Nielsen MB, Einarsen S. Outcomes of exposure to workplace bullying: A meta-analytic review. Work & Stress. 2012;26(4):309-32.
3. Quinlan E, Robertson S, Miller N, Robertson-Boersma D. Interventions to reduce bullying in health care organizations: a scoping review. Health Serv Manage Res. 2014;27(1-2):33-44. Epub 2015/01/17. doi: 10.1177/0951484814547236. PubMed PMID: 25595015.
4. Stagg SJ, Sheridan D. Effectiveness of bullying and violence prevention programs. AAOHN J. 2010;58(10):419-24. Epub 2010/09/30. doi: 10.3928/08910162-20100916-02. PubMed PMID: 20873686.
