# Supplementary material for: Prevention and management of unprofessional behaviour among adults in the workplace: A scoping review
Source: PLoS One. 2018 Jul 26;13(7):e0201187. doi: 10.1371/journal.pone.0201187 (PMC6062077; doi:10.1371/journal.pone.0201187)
Supplement: S3 Table — (PDF) [file pone.0201187.s003.pdf]

S3 Table. Individual Study Characteristics

Study	Country	Study Design	Setting	Single or multiple site	Total Sample Size
Anderson, 2006[1]	USA	Controlled before and after (CBA)	Healthcare Organization	Single	43
Barak, 1994[2]	Israel	Pre/Post Test (single group)	Public administration/Government	Multiple	25
Bingham, 2001[3]	USA	Quasi-randomized controlled trial (Quasi-RCT)	Educational Institution	Single	516
Ceravolo, 2012[4]	USA	Pre/Post Test (single group)	Healthcare Organization	Multiple	4032
Chipps, 2012[5]	USA	Pre/Post Test (single group)	Healthcare Organization	Multiple	16
Dahlby, 2014[6]	USA	Pre/Post Test (single group)	Healthcare Organization	Single	46
Dompierre, 2008[7]	Canada	Controlled before and after (CBA)	Educational Institution	Single	142
Embree, 2013[8]	USA	Pre/Post Test (single group)	Healthcare Organization	Single	135
Frisbie, 2002[9]	USA	Randomized controlled trial (RCT)	Private Company	Single	133
Goldberg, 2007[10]	USA	Cluster-randomized controlled trial (Cluster-RCT)	Educational Institution	Single	234
Hoel, 2006[11]	UK	Cluster-randomized controlled trial (Cluster-RCT)	Public administration/Government	Multiple	NR
Hultman, 2012[12]	USA	Pre/Post Test (single group)	Educational Institution	Single	104
Keashly, 2009[13]	USA	Controlled before and after (CBA)	Public administration/Government	Multiple	NR
Kennedy, 2010[14]	USA	Pre/Post Test (single group)	Educational Institution	Single	26
Lansbury, 2014[15]	UK	Controlled before and after (CBA)	Private Company	Multiple	1672
Leiter, 2011[16]	Canada	Non-randomized controlled trial (NRCT)	Healthcare Organization	Multiple	1173
Leon-Perez, 2012[17]	Spain	Pre/Post Test (single group)	Private Company	Single	42
Mallette, 2011[18]	Canada	Controlled before and after (CBA)	Healthcare Organization	Single	164
Meloni, 2011[19]	Australia	Pre/Post Test (single group)	Healthcare Organization	Single	NR

Osatuke, 2009[20]	USA	Controlled before and after (CBA)	Healthcare Organization	Multiple	2194
Pate, 2010[21]	UK	Pre/Post Test (single group)	Public administration/Government	Single	200
Sanderson, 2014[22]	USA	Pre/Post Test (single group)	Healthcare Organization	Single	108
Stagg, 2011[23]	USA	Pre/Post Test (single group)	Healthcare Organization	Multiple	20

References

1. Anderson C. Training efforts to reduce reports of workplace violence in a community health care facility. J Prof Nurs. 2006;22(5):289-95.
2. Barak A. A cognitive-behavioral educational workshop to combat sexual harassment in the workplace. J Couns Dev. 1994;72(6):595-602. doi: http://dx.doi.org/10.1002/j.1556-6676.1994.tb01688.x.
3. Bingham SG, Scherer LL. The unexpected effects of a sexual harassment educational program. J Appl Behav Sci. 2001;37(2):125-53.
4. Ceravolo DJ, Schwartz DG, Foltz-Ramos KM, Castner J. Strengthening communication to overcome lateral violence. J Nurs Manag. 2012;20(5):599-606. doi: http://dx.doi.org/10.1111/j.1365-2834.2012.01402.x.
5. Chipps EM, McRury M. The development of an educational intervention to address workplace bullying: a pilot study. J Nurses Staff Dev. 2012;28(3):94-8. doi: http://dx.doi.org/10.1097/NND.0b013e31825514bb.
6. Dahlby MA, Herrick LM. Evaluating an educational intervention on lateral violence. J Contin Educ Nurs. 2014;45(8):344-50; quiz 51-2. doi: http://dx.doi.org/10.3928/00220124-20140724-15.
7. Dompierre J, Laliberte D, Girard S, Gignac S. A qualitative and quantitative evaluation of an experiment for preventing violence in the workplace. Eur Rev Appl Psychol. 2008;58(4):275-83. doi: http://dx.doi.org/10.1016/j.erap.2008.09.010.
8. Embree JL, Bruner DA, White A. Raising the Level of Awareness of Nurse-to-Nurse Lateral Violence in a Critical Access Hospital. Nurs Res Pract. 2013;2013:207306. doi: http://dx.doi.org/10.1155/2013/207306.
9. Frisbie SH. Sexual harassment: A comparison of online versus traditional training methods. Dissertation Abstracts International: Section B: The Sciences and Engineering. 2002;62(10-B):4837. PubMed PMID: Dissertation Abstract: 2002-95008-310.
10. Goldberg CB. The impact of training and conflict avoidance on responses to sexual harassment. Psychol Women Q. 2007;31(1):62-72. doi: 10.1111/j.1471-6402.2007.00331.x.
11. Hoel H, Giga SI. Destructive interpersonal conflict in the workplace: The effectiveness of management interventions. Destructive Interpersonal Conflict in the Workplace: The Effectiveness of Management Interventions. 2006.
12. Hultman CS, Connolly A, Halvorson EG, Rowland P, Meyers MO, Mayer DC, et al. Get on your boots: preparing fourth-year medical students for a career in surgery, using a focused curriculum to teach the competency of professionalism. J Surg Res. 2012;177(2):217-23. doi: http://dx.doi.org/10.1016/j.jss.2012.06.019.
13. Keashly L, Neuman JH. Building a constructive communication climate: The Workplace Stress and Aggression Project. Destructive organizational communication: Processes, consequences, and constructive ways of organizing. New York, NY: Routledge/Taylor & Francis Group; US; 2009. p. 339-62.
14. Kennedy M. Workplace bullying: The enculturated group behavior of nurses Southern Nazarene University.
15. Lansbury L. The development, measurement and implementation of a bystander intervention strategy: A field study on workplace verbal bullying in a large UK organisation: University of Portsmouth; 2014.

16. Leiter MP, Laschinger HKS, Day A, Oore DG. The impact of civility interventions on employee social behavior, distress, and attitudes. J Appl Psychol. 2011;96(6):1258-74. doi: 10.1037/a0024442.

17. Leon-Perez JM, Arenas A, Griggs TB. Effectiveness of conflict management training to prevent workplace bullying. Workplace bullying: Symptoms and solutions. New York, NY: Routledge/Taylor & Francis Group; US; 2012. p. 230-43.

18. Mallette C, Duff M, McPhee C, Pollex H, Wood A. Workbooks to virtual worlds: a pilot study comparing educational tools to foster a culture of safety and respect in Ontario. Nurs Leadersh (Tor Ont). 2011;24(4):44-64.

19. Meloni M, Austin M. Implementation and outcomes of a zero tolerance of bullying and harassment program. Aust Health Rev. 2011;35(1):92-4. doi: http://dx.doi.org/10.1071/AH10896.

20. Osatuke K, Moore SC, Ward C, Dyrenforth SR, Belton L. Civility, respect, engagement in the workforce (CREW): Nationwide organization development intervention at veterans health administration. J Appl Behav Sci. 2009;45(3):384-410. doi: 10.1177/0021886309335067.

21. Pate J, Beaumont P. Bullying and harassment: A case of success? Employee Relations. 2010;32(2):171-83. doi: http://dx.doi.org/10.1108/01425451011010113.

22. Sanderson L. Improving civility in the mental health nursing workplace through assertiveness training with role-play. Dissertation Abstracts International: Section B: The Sciences and Engineering. 2014;74(11-B(E)):No Pagination Specified. PubMed PMID: Dissertation Abstract: 2014-99100-313.

23. Stagg SJ, Sheridan D, Jones RA, Speroni KG. Evaluation of a workplace bullying cognitive rehearsal program in a hospital setting. J Contin Educ Nurs. 2011;42(9):395-401; quiz 2-3. doi: http://dx.doi.org/10.3928/00220124-20110823-45.
