# Supplementary material for: Prevention and management of unprofessional behaviour among adults in the workplace: A scoping review
Source: PLoS One. 2018 Jul 26;13(7):e0201187. doi: 10.1371/journal.pone.0201187 (PMC6062077; doi:10.1371/journal.pone.0201187)
Supplement: S4 Table — (PDF) [file pone.0201187.s004.pdf]

S4 Table. Individual Participant Characteristics

Study	Arm Name	Sample size	Mean (SD/Range)	% female	Ethnicity (%)	Type of Employee
Anderson, 2006[1]	Intervention	22	NR (NR)	72.7	Black (0), Hispanic (13.6), Caucasian (86.4)	Line Worker/Staff
	Control	21	NR (NR)	95	Black (4.7), Hispanic (9.6), Caucasian (85.7)	Line Worker/Staff
Barak, 1994[2]	Intervention (single group)	25	NR (22 - 55)	1	NR	Line Worker/Staff
Bingham, 2001[3]	Intervention	197	NR (NR)	49.2	Total sample: Caucasian (89), Other (8), Unspecified (3)	Line Worker/Staff; Supervisor/Middle management
	Control	319	NR (NR)	55.8	Total sample: Caucasian (89), Other (8), Unspecified (3)	Line Worker/Staff; Supervisor/Middle management
Ceravolo, 2012[4]	Intervention (single group)	4032	NR (NR)	NR	NR	Line worker/staff; Supervisor/middle management
Chipps, 2012[5]	Intervention (single group)	16	NR (NR)	94	Caucasian (81)	Line Worker/Staff
Dahlby, 2014[6]	Intervention (single group)	46	NR (NR)	NR	NR	Line Worker/Staff
Dompierre, 2008[7]	Intervention	103	NR (NR)	53	NR	Line worker/staff; Other (unspecified school staff)
	Control	39	NR (NR)	56	NR	Line worker/staff; Other (unspecified school staff)
Embree, 2013[8]	Intervention (single group)	135	NR (NR)	NR	NR	Line Worker/Staff
Frisbie, 2002[9]	Intervention (Group 1)	45	NR (NR)	46.7	Caucasian (30), Hispanic (9), African American (3), Asian (0), Other (1), Missing (0)	Line Worker/Staff; Supervisor/Middle management
	Intervention (Group 2)	43	NR (NR)	48.8	Caucasian (28), Hispanic (7), African American (4), Asian (1), Other (2), Missing (1)	Line Worker/Staff; Supervisor/Middle management
	Control	45	NR (NR)	55.6	Caucasian (19), Hispanic (20), African American (5), Asian (0), Other (1), Missing (0)	Line Worker/Staff; Supervisor/Middle management
Goldberg, 2007[10]	Intervention	129	27 (4)	45	NR	Line Worker/Staff; Supervisor/Middle management
	Control	105	27 (4)	45	NR	Line Worker/Staff; Supervisor/Middle management
Hoel,	Control	NR	NR (NR)	NR	NR	Line Worker/Staff; Supervisor/Middle

Study	Arm Name	Sample size	Mean (SD/Range)	% female	Ethnicity (%)	Type of Employee
2006[11]						management; Other (NR)
	Intervention (Group 1)	NR	NR (NR)	NR	NR	Line Worker/Staff; Supervisor/Middle management; Other (NR)
	Intervention (Group 2)	NR	NR (NR)	NR	NR	Line Worker/Staff; Supervisor/Middle management; Other (NR)
	Intervention (Group 3)	NR	NR (NR)	NR	NR	Line Worker/Staff; Supervisor/Middle management; Other (NR)
	Intervention (Group 4)	NR	NR (NR)	NR	NR	Line Worker/Staff; Supervisor/Middle management; Other (NR)
Hultman, 2012[12]	Intervention (single group)	104	26.5 (24 to 33)	38.4	NR	Other (4th year medical students from general surgery, surgical subspecialties, obstetrics/ gynaecology, and anaesthesia)
Keashly, 2009[13]	Intervention	NR	NR (NR)	NR	NR	Line Worker/Staff; Supervisor/Middle management
	Control	NR	NR (NR)	NR	NR	Line Worker/Staff; Supervisor/Middle management
Kennedy, 2010[14]	Intervention (single group)	26	NR (NR)	96.2	NR	Other (RNs who were students in a graduate nursing program)
Lansbury, 2014[15]	Intervention (Group 1)	272	NR (NR)	NR	NR	Other (NR)
	Intervention (Group 2)	270	NR (NR)	NR	NR	Other (NR)
	Intervention (Group 3)	234	NR (NR)	NR	NR	Other (NR)
	Intervention (Group 4)	300	NR (NR)	NR	NR	Other (NR)
	Intervention (Group 5)	296	NR (NR)	NR	NR	Other (NR)
	Intervention (Group 6)	300	NR (NR)	NR	NR	Other (NR)
Leiter, 2011[16]	Intervention	262	NR (NR)	Total sample: 86%	NR	Line Worker/Staff; Supervisor/Middle management
	Control	911	NR (NR)	Total sample: 86%	NR	Line Worker/Staff; Supervisor/Middle management
Leon-Perez, 2012[17]	Intervention (single group)	42	NR (NR)	NR	NR	Supervisor/Middle management

Study	Arm Name	Sample size	Mean (SD/Range)	% female	Ethnicity (%)	Type of Employee
Mallette, 2011[18]	Intervention (Group 1)	33	NR (NR)	NR	NR	Line Worker/Staff
	Intervention (Group 2)	35	NR (NR)	NR	NR	Line Worker/Staff
	Intervention (Group 3)	31	NR (NR)	NR	NR	Line Worker/Staff
	Intervention (Group 4)	33	NR (NR)	NR	NR	Line Worker/Staff
	Control	32	NR (NR)	NR	NR	Line Worker/Staff
Meloni, 2011[19]	Intervention (single group)	NR	NR (NR)	NR	NR	Other (all hospital employees - unspecified)
Osatuke, 2009[20]	Intervention (Group 1)	899	NR (NR)	NR	NR	Line Worker/Staff
	Control	NR	NR (NR)	NR	NR	Line Worker/Staff
	Intervention (Group 2)	1295	NR (NR)	NR	NR	Line Worker/Staff
	Control	NR	NR (NR)	NR	NR	Line Worker/Staff
Pate, 2010[21]	Intervention (single group)	200	NR (NR)	NR	NR	Line Worker/Staff; Supervisor/Middle management; Executive/Upper management
Sanderson, 2014[22]	Intervention (single group)	108	NR (NR)	NR	NR	Line Worker/Staff; Supervisor/Middle management
Stagg, 2011[23]	Intervention (single group)	20	NR (NR)	1	NR	Line Worker/Staff

References

1. Anderson C. Training efforts to reduce reports of workplace violence in a community health care facility. J Prof Nurs. 2006;22(5):289-95.

2. Barak A. A cognitive-behavioral educational workshop to combat sexual harassment in the workplace. J Couns Dev. 1994;72(6):595-602. doi: http://dx.doi.org/10.1002/j.1556-6676.1994.tb01688.x.

3. Bingham SG, Scherer LL. The unexpected effects of a sexual harassment educational program. J Appl Behav Sci. 2001;37(2):125-53.

4. Ceravolo DJ, Schwartz DG, Foltz-Ramos KM, Castner J. Strengthening communication to overcome lateral violence. J Nurs Manag. 2012;20(5):599-606. doi: http://dx.doi.org/10.1111/j.1365-2834.2012.01402.x.

5. Chipps EM, McRury M. The development of an educational intervention to address workplace bullying: a pilot study. J Nurses Staff Dev. 2012;28(3):94-8. doi: http://dx.doi.org/10.1097/NND.0b013e31825514bb.

6. Dahlby MA, Herrick LM. Evaluating an educational intervention on lateral violence. J Contin Educ Nurs. 2014;45(8):344-50; quiz 51-2. doi: http://dx.doi.org/10.3928/00220124-20140724-15.

7. Dompierre J, Laliberte D, Girard S, Gignac S. A qualitative and quantitative evaluation of an experiment for preventing violence in the workplace. Eur Rev Appl Psychol. 2008;58(4):275-83. doi: http://dx.doi.org/10.1016/j.erap.2008.09.010.

8. Embree JL, Bruner DA, White A. Raising the Level of Awareness of Nurse-to-Nurse Lateral Violence in a Critical Access Hospital. Nurs Res Pract. 2013;2013:207306. doi: http://dx.doi.org/10.1155/2013/207306.

9. Frisbie SH. Sexual harassment: A comparison of online versus traditional training methods. Dissertation Abstracts International: Section B: The Sciences and Engineering. 2002;62(10-B):4837. PubMed PMID: Dissertation Abstract: 2002-95008-310.

10. Goldberg CB. The impact of training and conflict avoidance on responses to sexual harassment. Psychol Women Q. 2007;31(1):62-72. doi: 10.1111/j.1471-6402.2007.00331.x.

11. Hoel H, Giga SI. Destructive interpersonal conflict in the workplace: The effectiveness of management interventions. Destructive Interpersonal Conflict in the Workplace: The Effectiveness of Management Interventions. 2006.

12. Hultman CS, Connolly A, Halvorson EG, Rowland P, Meyers MO, Mayer DC, et al. Get on your boots: preparing fourth-year medical students for a career in surgery, using a focused curriculum to teach the competency of professionalism. J Surg Res. 2012;177(2):217-23. doi: http://dx.doi.org/10.1016/j.jss.2012.06.019.

13. Keashly L, Neuman JH. Building a constructive communication climate: The Workplace Stress and Aggression Project. Destructive organizational communication: Processes, consequences, and constructive ways of organizing. New York, NY: Routledge/Taylor & Francis Group; US; 2009. p. 339-62.

14. Kennedy M. Workplace bullying: The enculturated group behavior of nurses Southern Nazarene University.

15. Lansbury L. The development, measurement and implementation of a bystander intervention strategy: A field study on workplace verbal bullying in a large UK organisation: University of Portsmouth; 2014.

16. Leiter MP, Laschinger HKS, Day A, Oore DG. The impact of civility interventions on employee social behavior, distress, and attitudes. J Appl Psychol. 2011;96(6):1258-74. doi: 10.1037/a0024442.

17. Leon-Perez JM, Arenas A, Griggs TB. Effectiveness of conflict management training to prevent workplace bullying. Workplace bullying: Symptoms and solutions. New York, NY: Routledge/Taylor & Francis Group; US; 2012. p. 230-43.

18. Mallette C, Duff M, McPhee C, Pollex H, Wood A. Workbooks to virtual worlds: a pilot study comparing educational tools to foster a culture of safety and respect in Ontario. Nurs Leadersh (Tor Ont). 2011;24(4):44-64.

19. Meloni M, Austin M. Implementation and outcomes of a zero tolerance of bullying and harassment program. Aust Health Rev. 2011;35(1):92-4. doi: http://dx.doi.org/10.1071/AH10896.

20. Osatuke K, Moore SC, Ward C, Dyrenforth SR, Belton L. Civility, respect, engagement in the workforce (CREW): Nationwide organization development intervention at veterans health administration. J Appl Behav Sci. 2009;45(3):384-410. doi: 10.1177/0021886309335067.

21. Pate J, Beaumont P. Bullying and harassment: A case of success? Employee Relations. 2010;32(2):171-83. doi: http://dx.doi.org/10.1108/01425451011010113.

22. Sanderson L. Improving civility in the mental health nursing workplace through assertiveness training with role-play. Dissertation Abstracts International: Section B: The Sciences and Engineering. 2014;74(11-B(E)):No Pagination Specified. PubMed PMID: Dissertation Abstract: 2014-99100-313.

23. Stagg SJ, Sheridan D, Jones RA, Speroni KG. Evaluation of a workplace bullying cognitive rehearsal program in a hospital setting. J Contin Educ Nurs. 2011;42(9):395-401; quiz 2-3. doi: http://dx.doi.org/10.3928/00220124-20110823-45.
