# Supplementary material for: Prevention and management of unprofessional behaviour among adults in the workplace: A scoping review
Source: PLoS One. 2018 Jul 26;13(7):e0201187. doi: 10.1371/journal.pone.0201187 (PMC6062077; doi:10.1371/journal.pone.0201187)
Supplement: S5 Table — (PDF) [file pone.0201187.s005.pdf]

S5 Table. Individual Study Outcomes

Knowledge of or Attitudes to Workplace Bullying/Incivility

**Study**	**Bingham, 2001[1]**			
**Overall Results**	The intervention significantly improved attitudes to sexual harassment, though there were significant interactions with gender			
**Tool or Scale**	Questionnaire on knowledge and attitudes to sexual harassment			
**Effect measure**	Mean (SD): Higher score indicates stronger agreement with statement			
Outcome	**Attitude That Sexual Behavior Is Inappropriate**			
	Intervention		***Control***	***Between Groups Comparison***
Sample size	*Results [Mean (SD)]*	*Sample size*	*Results [Mean (SD)]*	
Total: 195	3.77 (0.81)	Total: 319	3.64 (0.82)	ANOVA (Intervention status): F(1, 510) = 4.96, p<0.05
Outcome	**Attributions of blame for sexual harassment**			
	Intervention		***Control***	***Between Groups Comparison***
Sample size	*Results [Mean (SD)]*	*Sample size*	*Results [Mean (SD)]*	
Male: 99<br>Female: 97	Male: 2.71 (SD)<br>Female: 2.32 (0.5)	Male: 141<br>Female: 178	Male: 2.52 (0.52)<br>Female: 2.44 (0.55)	ANOVA (Intervention status x gender): F(1, 511) = 9.17, p<0.01
Outcome	**Knowledge That Sexual Behavior is Inappropriate**			
	Intervention		***Control***	***Between Groups Comparison***
Sample size	*Results [Mean (SD)]*	*Sample size*	*Results [Mean (SD)]*	
Total: 197	4.33 (0.81)	Total: 319	3.94 (0.99)	ANOVA (Intervention status): F(1, 512) = 21.53, p<0.0001
Outcome	**Perceptions of potential sexual harassment**			
	Intervention		***Control***	***Between Groups Comparison***
Sample size	*Results [Mean (SD)]*	*Sample size*	*Results [Mean (SD)]*	
Male: 100<br>Female: 97	Male: 2.78 (0.75)<br>Female: 2.98 (0.14)	Male: 141<br>Female: 178	Male: 2.90 (0.42)<br>Female: 2.89 (0.49)	ANOVA (Intervention status x gender): F(1, 512) = 6.49, P<0.01
Outcome	**Willingness to Report Sexual Harassment**			
	Intervention		***Control***	***Between Groups Comparison***
Sample size	*Results [Mean (SD)]*	*Sample size*	*Results [Mean (SD)]*	
Male: 100<br>Female: 97	Male: 1.25 (0.76)<br>Female: 1.45 (0.68)	Male: 141<br>Female: 178	Male: 1.52 (0.66)<br>Female: 1.48 (0.66)	ANOVA (Intervention status x gender): F(1, 512) = 3.87, P<0.05

**Study**	**Ceravolo, 2012[2]**			
**Overall Results**	Positive trends in awareness of workplace bullying and culture in the workplace (no formal statistical comparison was conducted)			
**Tool or Scale**	Verbal Abuse Survey			
**Effect measure**	n (%): proportion of respondents who agree or strongly agree with statement			
Outcome	**Believed that verbal abuse would influence their overall delivery of nursing care**			
Intervention		***Control***		***Between Groups Comparison***
Sample size	*Results [n (%)]*	*Sample size*	*Results [n (%)]*	
Pre: 703<br>Post: 485	Pre: 276 (42%)<br>Post: 204 (63%)	-	-	NA
Outcome	**Work is a safe environment to express my opinions**			
Intervention		***Control***		***Between Groups Comparison***
Sample size	*Results [n (%)]*	*Sample size*	*Results [n (%)]*	
Pre: 703<br>Post: 485	Pre: 317 (65%)<br>Post: 408 (52%)	-	-	NA
Outcome	**Peers respect my opinion**			
Intervention		***Control***		***Between Groups Comparison***
Sample size	*Results [n (%)]*	*Sample size*	*Results [n (%)]*	
Pre: 703<br>Post: 485	Pre: 512 (65%)<br>Post: 356 (74%)	-	-	NA
Outcome	**Feel respected by peers**			
Intervention		***Control***		***Between Groups Comparison***
Sample size	*Results [n (%)]*	*Sample size*	*Results [n (%)]*	
Pre: 703<br>Post: 485	Pre: 430 (88%)<br>Post: 616 (78%)	-	-	NA
Outcome	**Feel supported by peers**			
Intervention		***Control***		***Between Groups Comparison***
Sample size	*Results [n (%)]*	*Sample size*	*Results [n (%)]*	
Pre: 703<br>Post: 485	Pre: 590 (75)<br>Post: 423 (87)	-	-	NA

Outcome	**Have good working relationships**				
	Intervention		***Control***		***Between Groups Comparison***
Sample size	*Results [n (%)]*	*Sample size*	*Results [n (%)]*		
Pre: 703<br>Post: 485	Pre: 510 (65)<br>Post: 378 (78)	-	-		NA
Outcome	**Feel high levels of control over practice**				
	Intervention		***Control***		***Between Groups Comparison***
Sample size	*Results [n (%)]*	*Sample size*	*Results [n (%)]*		
Pre: 703<br>Post: 485	Pre: 276 (40)<br>Post: 204 (42)	-	-		NA
Outcome	**Have high levels of self-esteem**				
	Intervention		***Control***		***Between Groups Comparison***
Sample size	*Results [n (%)]*	*Sample size*	*Results [n (%)]*		
Pre: 703<br>Post: 485	Pre: 348 (49)<br>Post: 208 (43)	-	-		NA
**Study**	**Chipps, 2012[3]**				
**Overall Results**	Positive trends in the awareness of causes/contributors to workplace bullying (not tested)				
**Tool or Scale**	Participant Survey				
**Effect measure**	n (%): proportion of respondents who agree or strongly agree with statement				
Outcome	**Believed near errors occurred because of negative behaviours**				
	Intervention		***Control***		***Between Groups Comparison***
Sample size	*Results [n (%)]*	*Sample size*	*Results [n (%)]*		
Pre: 16<br>Post: 16	Pre: NR (13%)<br>Post: NR (19%)	-	-		NA
Outcome	**Believed patient safety was compromised secondary to negative acts**				
	Intervention		***Control***		***Between Groups Comparison***

Sample size	*Results [n (%)]*	*Sample size*	*Results [n (%)]*	
Pre: 16<br>Post: 16	Pre: NR (6%)<br>Post: NR (13%)	-	-	NA
Outcome	**Job Satisfaction**			
	Intervention		***Control***	***Between Groups Comparison***
Sample size	*Results [n (%)]*	*Sample size*	*Results [n (%)]*	
Pre: 16<br>Post: 16	Pre: NR (81%)<br>Post: NR (81%)	-	-	NA

**Study**	**Dahlby, 2014[4]**			
**Overall Results**	Positive trends in understanding of causes/contributors to workplace bullying (non-significant)			
**Tool or Scale**	Lateral and Vertical Violence in Nursing Survey			
**Effect measure**	N (%): proportion of respondents who agree or strongly agree with statement			
Outcome	**Fear of retaliation: I feel safe from retaliation when reporting an episode of lateral violence**			
	Intervention		***Control***	***Between Groups Comparison***
Sample size	*Results [n (%)]*	*Sample size*	*Results [n (%)]*	
Pre: 29<br>Post: 25	Pre: 11 (37.93%)<br>Post: 12 (50%)<br>Pre v Post: p=0.38	-	-	NA
Outcome	**Inadequate staff or resources to handle the workload contributes to lateral violence in my work unit**			
	Intervention		***Control***	***Between Groups Comparison***
Sample size	*Results [n (%)]*	*Sample size*	*Results [n (%)]*	
Pre: 29<br>Post: 25	Pre: 24 (82.76%)<br>Post: 22 (88%)<br>Pre v Post: p=0.59	-	-	NA
Outcome	**Job stress leading to loss of control over behaviour contributes to lateral violence in my work unit**			
	Intervention		***Control***	***Between Groups Comparison***
Sample size	*Results [n (%)]*	*Sample size*	*Results [n (%)]*	

Sample size	Results [n (%)]	Sample size	Results [n (%)]	Between Groups Comparison
Pre: 29<br>Post: 25	Pre: 19 (65.52%)<br>Post: 16 (64%)<br>Pre v Post: p=0.91	-	-	NA

Outcome	**Leaders not willing to intervene contributes to lateral violence in my work unit**			
Intervention		***Control***		***Between Groups Comparison***
Sample size	*Results [n (%)]*	*Sample size*	*Results [n (%)]*	
Pre: 29<br>Post: 25	Pre: 24 (82.76%)<br>Post: 19 (76%)<br>Pre v Post: P=0.54	-	-	NA

Outcome	**Major personality clashes contribute to lateral violence in my work unit**			
Intervention		***Control***		***Between Groups Comparison***
Sample size	*Results [n (%)]*	*Sample size*	*Results [n (%)]*	
Pre: 29<br>Post: 25	Pre: 17 (58.62%)<br>Post: 20 (80%)<br>Pre v Post: p=0.09	-	-	NA

Outcome	**Misunderstandings related to cultural differences contribute to lateral violence in my work unit**			
Intervention		***Control***		***Between Groups Comparison***
Sample size	*Results [n (%)]*	*Sample size*	*Results [n (%)]*	
Pre: 29<br>Post: 25	Pre: 5 (17.24%)<br>Post: 5 (20%)<br>Pre v Post: p=0.79	-	-	NA

Outcome	**Misunderstandings related to gender contribute to lateral violence in my work unit**			
Intervention		***Control***		***Between Groups Comparison***
Sample size	*Results [n (%)]*	*Sample size*	*Results [n (%)]*	
Pre: 29<br>Post: 25	Pre: 2 (6.9%)<br>Post: 5 (20%)<br>Pre v Post: p=0.15	-	-	NA

Outcome	**Peers not willing to intervene contributes to lateral violence in my work unit**			
Intervention		***Control***		***Between Groups Comparison***
Sample size	*Results [n (%)]*	*Sample size*	*Results [n (%)]*	
Pre: 29<br>Post: 25	Pre: 20 (68.97%)<br>Post: 14 (56%)<br>Pre v Post: p=0.33	-	-	NA

Outcome	**Power and control issues contribute to lateral violence in my work unit**

Intervention		*Control*		*Between Groups Comparison*
Sample size	*Results [n (%)]*	*Sample size*	*Results [n (%)]*	
Pre: 29<br>Post: 25	Pre: 20 (68.97%)<br>Post: 18 (72%)<br>Pre v Post: p=0.81	-	-	NA

Outcome	**Rude behaviour contributes to lateral violence in my work unit**

Intervention		*Control*		*Between Groups Comparison*
Sample size	*Results [n (%)]*	*Sample size*	*Results [n (%)]*	
Pre: 29<br>Post: 25	Pre: 16 (55.17%)<br>Post: 13 (52%)<br>Pre v Post: p=0.82	-	-	NA

Outcome	**Targeted nurse not willing to stand up to perpetrator contributes to lateral violence in my work unit**

Intervention		*Control*		*Between Groups Comparison*
Sample size	*Results [n (%)]*	*Sample size*	*Results [n (%)]*	
Pre: 29<br>Post: 25	Pre: 19 (65.52%)<br>Post: 12 (48%)<br>Pre v Post: p=0.19	-	-	NA

**Study**	**Dompierre, 2008[5]**
**Overall Results**	Intervention participants demonstrated less positive perceptions of the workplace (significant), indicating greater awareness of workplace violence
**Tool or Scale**	Participant Survey
**Effect measure**	Mean (SD): higher score indicates a more positive perception/stronger agreement with the statement
Outcome	**Global index of the perception of the collectivity**

Intervention		*Control*		*Between Groups Comparison*
Sample size	*Results [Mean (SD)]*	*Sample size*	*Results [Mean (SD)]*	
Pre: 103<br>Post: 51	Pre: 62.51, (NR)<br>Post: 62.43, (NR)<br>Pre v. Post [t(df)]: 0.06 (152), p=NS	Pre: NA<br>Post: 39	Pre: NA, (NA)<br>Post: 65, (NR)	Intervention v. Control [t-test (df)]: -2.01 (88), p<0.01

Outcome	**The perception of putting in place measures of security: “it takes too long to resolve problems”**

	Intervention		Control		Between Groups Comparison
	Sample size	*Results [Mean (SD)]*	*Sample size*	*Results [Mean (SD)]*	
	Pre: 103<br>Post: 51	NR	Pre: NA<br>Post: 39	NR	Intervention v. Control [t-test (df)]:<br>2.17 (NR), p=0.033
Outcome	**Response to statement: “violence is considered to be part of the job”**				
	Intervention		***Control***		***Between Groups Comparison***
	Sample size	*Results [Mean (SD)]*	*Sample size*	*Results [Mean (SD)]*	
	Pre: 103<br>Post: 51	NR	Pre: NA<br>Post: 39	NR	Intervention v. Control [t-test (df)]:<br>3.44 (NR), p=0.0009

**Study**	**Embree 2013[6]**				
**Overall Results**	Post-survey responses showed improved individual awareness of and responses to lateral violence and less feelings of oppression, these changes were not reflected in overall group scores				
**Tool or Scale**	Nurse Workplace Behaviour Scale (NWS)				
**Effect measure**	Mean (SD): Higher scores indicate greater perceptions of self-empowerment				
Outcome	**Total Nurse Workplace Behaviour Scale (NWS)**				
	Intervention		***Control***		***Between Groups Comparison***
	Sample size	*Results [Mean (SD)]*	*Sample size*	*Results [Mean (SD)]*	
	Pre: 44<br>Post: 33	Pre : 27.98, (NR)<br>Post : 28.73, (NR)	-	-	NA
Outcome	**NWS - 'Internalized Sexism' subscale**				
	Intervention		***Control***		***Between Groups Comparison***
	Sample size	*Results [Mean (SD)]*	*Sample size*	*Results [Mean (SD)]*	
	Pre: 44<br>Post: 33	Pre : 8.95, (NR)<br>Post : 8.67, (NR)	-	-	NA
Outcome	**NWS - 'Minimization of Self' subscale**				
	Intervention		***Control***		***Between Groups Comparison***
	Sample size	*Results [Mean (SD)]*	*Sample size*	*Results [Mean (SD)]*	

Pre: 44<br>Post: 33	Pre : 19.02, (NR)<br>Post : 20.06, (NR)	-	-	NA

**Study**	**Frisbie, 2002[7]**
**Overall Results**	The intervention significantly increased knowledge of sexual harassment, ability to perceive sexual harassment behaviours, and significantly decreased tolerance for sexual harassment behaviours.
**Tool or Scale**	Harassment Sensitivity Inventory (HSI)
**Effect measure**	Mean (SD): Higher scores indicate increased awareness of sexual harassment
Outcome	**Harassment Sensitivity Inventory (HSI) – Total**

Intervention		***Control***		***Between Groups Comparison***
Sample size	*Results [Mean (SD)]*	*Sample size*	*Results [Mean (SD)]*	
Group 1: total- 44;<br>female- 21; male- 23<br>Group 2: total- 43;<br>female- 21; male- 22	Group 1: total- 5.22 (0.62); female- 5.27 (0.54);<br>male- 5.18 (0.69)<br>Group 2: total- 5.34 (0.53); female- 5.21 (0.59)<br>male- 5.46 (0.44)	Total: 45<br>Female: 25<br>Male: 20	Total : 4.93 (0.71)<br>Female : 5.13 (0.61)<br>Male : 4.69 (0.77)	Group 1 v Group 2 v Control [F-test (df)]: 5.77, p=0.004<br>Group 1 total v Group 2 total [Mean Difference]: 0.12, p=1.00<br>Group 1 total v Control total [Mean Difference]: 0.29, p=0.084<br>Group 2 total v Control total [Mean Difference]: 0.41, p=0.007

Outcome	**Harassment Sensitivity Inventory (HSI) – Hostile Subscale**

Intervention		***Control***		***Between Groups Comparison***
Sample size	*Results [Mean (SD)]*	*Sample size*	*Results [Mean (SD)]*	
Group 1: total- 44;<br>female- 21; male- 23<br>Group 2: total- 43;<br>female- 21; male- 22	Group 1 : total- 5.14 (0.71);<br>female- 5.2 (0.61);<br>male- 5.08 (0.8)<br>Group 2 : total- 5.25 (0.61);<br>female- 5.13 (0.63)<br>male- 5.36 (0.57)	Total: 45<br>Female: 25<br>Male: 20	Total : 4.71 (0.79)<br>Female : 4.94 (0.65)<br>Male : 4.42 (0.88)	Group 1 v Group 2 v Control [F-test (df)]: 8.807, p<0.001<br>Group 1 total v Group 2 total [Mean Difference]:NR<br>Group 1 total v Control total [Mean Difference]: 0.43, p=0.014<br>Group 2 total v Control total [Mean Difference]: 0.54, p=0.001

Outcome	**Harassment Sensitivity Inventory (HSI) – Interfering Subscale**

Intervention		***Control***		***Between Groups Comparison***
Sample size	*Results [Mean (SD)]*	*Sample size*	*Results [Mean (SD)]*	

Group 1: total- 44;<br>female- 21; male- 23<br>Group 2: total- 43;<br>female- 21; male- 22	Group 1 : total- 5.16 (0.66);<br>female- 5.24 (0.59);<br>male- 5.08 (0.73)<br>Group 2 : total- 5.34 (0.56);<br>female- 5.15 (0.67)<br>male- 5.52 (0.37)	Total: 45<br>Female: 25<br>Male: 20	Total : 5.02 (0.78)<br>Female : 5.17 (0.7)<br>Male : 4.84 (0.84)	Group 1 v Group 2 v Control [F-test (df)]:<br>2.78, p=0.066<br>Group 1 total v Group 2 total<br>[Mean Difference]: NR<br>Group 1 total v Control total<br>[Mean Difference]: NR<br>Group 2 total v Control total<br>[Mean Difference]: NR
Outcome	**Harassment Sensitivity Inventory (HSI) – Intimidating Subscale**			
Intervention		***Control***		***Between Groups Comparison***
Sample size	*Results [Mean (SD)]*	*Sample size*	*Results [Mean (SD)]*	
Group 1: total- 44;<br>female- 21; male- 23<br>Group 2: total- 43;<br>female- 21; male- 22	Group 1 : total- 5.29 (0.61);<br>female- 5.32 (0.53);<br>male- 5.27 (0.68)<br>Group 2 : total- 5.4 (0.51);<br>female- 5.31 (0.59)<br>male- 5.49 (0.42)	Total: 45<br>Female: 25<br>Male: 20	Total : 5.1 (0.69)<br>Female : 5.31 (0.58)<br>Male : 4.84 (0.75)	Group 1 v Group 2 v Control [F-test (df)]:<br>3.37, p<0.038<br>Group 1 total v Group 2 total<br>[Mean Difference]: NR<br>Group 1 total v Control total<br>[Mean Difference]: NR<br>Group 2 total v Control total<br>[Mean Difference]: NR
Outcome	**Harassment Sensitivity Inventory (HSI) – Offensive Subscale**			
Intervention		***Control***		***Between Groups Comparison***
Sample size	*Results [Mean (SD)]*	*Sample size*	*Results [Mean (SD)]*	
Group 1: total- 44;<br>female- 21; male- 23<br>Group 2: total- 43;<br>female-21; male- 22	Group 1 : total- 5.3 (0.63); female-<br>5.31 (0.58);<br>male- 5.29 (0.68)<br>Group 2 : total- 5.37 (0.52);<br>female- 5.25 (0.58)<br>male- 5.47 (0.44)	Total: 45<br>Female: 25<br>Male: 20	Total : 4.9 (0.78)<br>Female : 5.1 (0.61)<br>Male : 4.65 (0.91)	Group 1 v Group 2 v Control [F-test (df)]:<br>7.54, p<0.001<br>Group 1 total v Group 2 total<br>[Mean Difference]: NR<br>Group 1 total v Control total<br>[Mean Difference]: 0.4, p=0.012<br>Group 2 total v Control total<br>[Mean Difference]: 0.47, p=0.003
**Tool or Scale**	Sexual Harassment Attitude Scale			
**Effect measure**	Mean (SD): Higher scores indicate more tolerant attitudes to sexual harassment			
Outcome	**Sexual Harassment Attitude Scale**			
Intervention		***Control***		***Between Groups Comparison***
Sample size	*Results [Mean (SD)]*	*Sample size*	*Results [Mean (SD)]*	

Group 1: total- 44;<br>female- 21; male- 23<br>Group 2: total- 43;<br>female- 21; male- 22	Group 1 : total- 2.92 (0.79); female- 2.81 (0.6);<br>male- 3.01 (0.93)<br>Group 2 : total- 2.72 (0.72); female- 2.49 (0.62);<br>male- 2.95 (0.76)	Total: 45<br>Female: 25<br>Male: 20	Total : 3.25 (0.94)<br>Female : 3.1 (0.85)<br>Male : 3.45 (1.04)	Group 1 v Group 2 v Control [F-test (df)]: 5.24 (2), p=0.007<br>Group 1 total v Group 2 total<br>[Mean Difference]: 0.19, p=0.821<br>Group 1 total v Control total<br>[Mean Difference]: 0.34, p=0.158<br>Group 2 total v Control total<br>[Mean Difference]: 0.53, p=0.008
**Tool or Scale**	Sexual Harassment Knowledge			
**Effect measure**	Mean (SD): Higher scores indicate more knowledge of sexual harassment behaviours			
Outcome	**Sexual Harassment Knowledge**			
Intervention		***Control***		***Between Groups Comparison***
Sample size	*Results [Mean (SD)]*	*Sample size*	*Results [Mean (SD)]*	
Group 1: total- 44;<br>female- 21; male- 23<br>Group 2: total- 43;<br>female- 21; male- 22	Group 1 : total- 14.45 (2.43); female- 14.05 (2.44);<br>male- 14.83 (2.41)<br>Group 2 : total- 15.6 (1.97); female- 15.19 (1.69)<br>male- 16 (2.16)	Total: 45<br>Female: 25<br>Male: 20	Total : 12.62 (2.72)<br>Female : 11.92 (3.01)<br>Male : 13.5 (2.06)	Group 1 v Group 2 v Control [F-test (df)]: 16.61 (2), p<0.0001<br>Group 1 total v Group 2 total<br>[Mean Difference]: 1.15, p=0.074<br>Group 1 total v Control total<br>[Mean Difference]: 1.83, p<0.001<br>Group 2 total v Control total<br>[Mean Difference]: 2.98, p<0.0001
**Study**	**Goldberg, 2007[8]**			
**Overall Results**	NR (no results were reported for this specific outcome)			
**Tool or Scale**	Sexual Experiences Questionnaire			
**Study**	**Hoel, 2006[9]**			
**Overall Results**	The interventions did not appear to have any significant impact on psychological fulfillment or satisfaction at work			
**Tool or Scale**	Measure of psychological contract fulfillment			
**Effect measure**	Mean (SD): Higher scores indicate higher levels of fulfillment			
Outcome	**Psychological contract – (Minimum score on scale = 7)**			
Intervention		***Control***		***Between Groups Comparison***
Sample size	*Results [Mean (SD)]*	*Sample size*	*Results [Mean (SD)]*	

Sample size	Results [Mean (SD)]	Sample size	Results [Mean (SD)]	Between Groups Comparison
Group 1: Baseline- NR; Post- NR<br>Group 2: Baseline- NR; Post- NR<br>Group 3: Baseline- NR; Post- NR<br>Group 4: Baseline- NR; Post- NR	Group 1: Baseline- 21.96 (NR); Post- 21.16 (NR)<br>Group 2: Baseline- 22.02 (NR); Post- 21.78 (NR)<br>Group 3: Baseline- 23.15 (NR); Post- 22.86 (NR)<br>Group 4: Baseline- 20.86 (NR); Post- 21.17 (NR)	Baseline: NR<br>Post-intervention: NR	Baseline: 21.17 (NR)<br>Post-intervention: 20.95 (NR)	NR

Tool or Scale Bullying Risk Assessment Tool (BRAT)

Effect measure Mean change (SD): Higher scores indicate more satisfaction with workplace

Outcome **Satisfaction with atmosphere at work compared with six months ago**

Intervention		***Control***		***Between Groups Comparison***
Sample size	*Results [Mean (SD)]*	*Sample size*	*Results [Mean (SD)]*	
Group 1: Baseline- NR; Post- NR<br>Group 2: Baseline- NR; Post- NR<br>Group 3: Baseline- NR; Post- NR<br>Group 4: Baseline- NR; Post- NR	Group 1: Baseline -0.13 (NR); Post -0.20 (NR)<br>Group 2: Baseline -0.10 (NR); Post -0.29 (NR)<br>Group 3: Baseline -0.01 (NR); Post -0.17 (NR)<br>Group 4: Baseline -0.18 (NR); Post -0.22 (NR)	Baseline: NR<br>Post-intervention: NR	Baseline: -0.12 (NR)<br>Post-intervention: -0.14 (NR)	NR

Study **Hultman, 2012[10]**

Overall Results The intervention significantly increased knowledge of unprofessional behaviour, identify attributes of a professional, integrate concepts of professionalism into practice, and understand the importance of professionalism

Tool or Scale Participant Survey

Effect measure Mean (SD): Higher scores indicate stronger agreement with the statement

Outcome **The course significantly improved their ability to define professionalism**

Intervention		***Control***		***Between Groups Comparison***
Sample size	*Results [Mean (SD)]*	*Sample size*	*Results [Mean (SD)]*	
Pre: NR<br>Post: 24	Pre : 3.46 (NR)<br>Post : 4.29 (NR)<br>Pre v Post: P<0.01	-	-	NA

Outcome **The course significantly improved their ability to identify attributes of the professional**

	Intervention		Control		Between Groups Comparison
	Sample size	*Results [Mean (SD)]*	*Sample size*	*Results [Mean (SD)]*	
	Pre: NR<br>Post: 24	Pre : 3.75 (NR)<br>Post : 4.5 (NR)<br>Pre v Post: P<0.01	-	-	NA
Outcome	**The course significantly improved their ability to integrate these concepts into practice**				
	Intervention		*Control*		***Between Groups Comparison***
	Sample size	*Results [Mean (SD)]*	*Sample size*	*Results [Mean (SD)]*	
	Pre: NR<br>Post: 24	Pre : 3.71 (NR)<br>Post : 4.49 (NR)<br>Pre v Post: P<0.01	-	-	NA
Outcome	**The course significantly improved their ability to understand the importance of professionalism**				
	Intervention		*Control*		***Between Groups Comparison***
	Sample size	*Results [Mean (SD)]*	*Sample size*	*Results [Mean (SD)]*	
	Pre: NR<br>Post: 24	Pre : 4.17 (NR)<br>Post : 4.67 (NR)<br>Pre v Post: P<0.01	-	-	NA
**Study**	**Kennedy, 2010[11]**				
**Overall Results**	Significant increase in knowledge of workplace bullying				
**Tool or Scale**	Demographic inventory and workplace bullying survey				
**Effect measure**	Mean (SD): Higher score indicates increase in knowledge				
Outcome	**Total Knowledge Score**				
	Intervention		*Control*		***Between Groups Comparison***
	Sample size	*Results [Mean (SD)]*	*Sample size*	*Results [Mean (SD)]*	
	Pre: 26<br>Post: 26	Pre: 106.85 (10.06)<br>Post: 121.24 (8.02)<br>Pre v Post [Mean difference (SD)]: -14.47 (8.78)<br>Pre v Post [t]: -8.41, p<0.05	-	-	NA
**Study**	**Lansbury, 2014[12]**				
**Overall Results**	Results were largely non-significant with only one intervention resulting in significant increase in participants' willingness to intervene in verbal bullying				

**Tool or Scale**	Responsible Bystander Intervention in Verbal Bullying (RBI-VB)
**Effect measure**	Mean (SD): Higher score indicates stronger agreement with statement

Outcome **Audience Variable - 'Other people think verbal bullying is unacceptable'**

Intervention		***Control***		***Between Groups Comparison***
Sample size	*Results [Mean (SD)]*	*Sample size*	*Results [Mean (SD)]*	
Group 1: Pre- 72; Post- 76<br>Group 2: Pre- 69; Post- 191<br>Group 3: Pre- 74; Post- 54<br>Group 4: Pre- 68; Post- 56<br>Group 5: Pre- 57; Post- 40<br>Group 6: Pre- 145; Post- 115	Group 1: Pre- 4.07 (1.30); Post- 3.82 (1.40)<br>Group 2: Pre- 4.01 (1.37); Post- 3.90 (1.19)<br>Group 3: Pre- 3.82 (1.13); Post- 3.93 (1.36)<br>Group 4: Pre- 3.78 (1.33); Post- 3.97 (1.25)<br>Group 5: Pre- 4.32 (1.00); Post- 4.22 (1.07)<br>Group 6: Pre- 4.14 (1.33); Post- 4.07 (1.32)	-	-	NR

Outcome **Responsible Bystander Intervention in Verbal Bullying (RBI-VB) – Power sub-scale**

Intervention		***Control***		***Between Groups Comparison***
Sample size	*Results [Mean (SD)]*	*Sample size*	*Results [Mean (SD)]*	
Group 1: Pre- 85; Post- 90<br>Group 2: Pre- 80; Post- 211<br>Group 3: Pre- 79; Post- 57<br>Group 4: Pre- 74; Post- 64<br>Group 5: Pre- 63; Post- 42<br>Group 6: Pre- 165; Post- 131	Group 1: Pre- 13.70 (4.06); Post- 13.66 (3.74)<br>Group 2: Pre- 13.66 (4.51); Post- 14.33 (4.13)<br>Group 3: Pre- 13.74 (4.25); Post- 13.30 (4.54)<br>Group 4: Pre- 13.41 (4.43); Post- 14.09 (3.79)<br>Group 5: Pre- 15.11 (4.21); Post- 14.95 (3.92)<br>Group 6: Pre- 14.40 (4.27); Post- 14.67 (3.96)	-	-	NR

Outcome **Responsible Bystander Intervention in Verbal Bullying (RBI-VB) – Process sub-scale**

Intervention		***Control***		***Between Groups Comparison***
Sample size	*Results [Mean (SD)]*	*Sample size*	*Results [Mean (SD)]*	
Group 1: Pre- 85; Post- 90<br>Group 2: Pre- 80; Post- 211<br>Group 3: Pre- 79; Post- 57<br>Group 4: Pre- 74; Post- 64<br>Group 5: Pre- 63; Post- 42<br>Group 6: Pre- 165; Post- 131	Group 1: Pre- 16.24 (5.07); Post- 16.80 (4.11)<br>Group 2: Pre- 16.70 (4.85); Post- 17.42 (4.66)<br>Group 3: Pre- 15.73 (4.64); Post- 15.37 (4.91)<br>Group 4: Pre- 16.86 (4.77); Post- 17.87 (4.99)<br>Group 5: Pre- 18.35 (4.91); Post- 17.41 (5.04)<br>Group 6: Pre- 18.33 (4.92); Post- 18.71 (4.96)	-	-	NR

Outcome **Willingness to intervene - 'If I see verbal bullying I will intervene'**

Intervention		***Control***		***Between Groups***

Sample size	Results [Mean (SD)]	Sample size	Results [Mean (SD)]	Comparison
Group 1: Pre- 74; Post- 85<br>Group 2: Pre- 79; Post- 202<br>Group 3: Pre- 77; Post- 57<br>Group 4: Pre- 71; Post- 59<br>Group 5: Pre- 63; Post- 41<br>Group 6: Pre- 153; Post- 125	Group 1: Pre- 3.86 (1.24); Post- 3.37 (1.43);<br>Group 1 Pre v Post: t= 2.17, p=0.03<br>Group 2: Pre- 3.97 (1.29); Post- 3.79 (1.17);<br>Group 2 Pre v Post: t= 1.12, p= 0.27<br>Group 3: Pre- 3.81 (1.38); Post- 4.00 (1.34);<br>Group 3 Pre v Post: t= -0.75, p= 0.48<br>Group 4: Pre- 3.79 (1.21); Post- 3.94 (1.27);<br>Group 4 Pre v Post: t= -0.66, p= 0.51<br>Group 5: Pre- 4.21 (1.02); Post- 4.37 (0.81);<br>Group 5 Pre v Post: t= -0.84, p= 0.40<br>Group 6: Pre- 3.87 (1.38); Post- 3.91 (1.43);<br>Group 6 Pre v Post: t= -0.23, p= -0.82	-	-	NR

**Study**	**Leiter, 2011[13]**
**Overall Results**	The intervention significantly improved civility scores, commitment, employee satisfaction, trust in management, and significanlty decreased measures of cynicism
**Tool or Scale**	CREW Civility Scale
**Effect measure**	Mean (SD): higher scores indicate perceptions of greater civility in the workplace
Outcome	**Civility**

Intervention		***Control***		***Between Groups Comparison***
Sample size	*Results [Mean (SD)]*	*Sample size*	*Results [Mean (SD)]*	
Time 1: 262<br>Time 2: 181	Time 1: 3.58 (0.73)<br>Time 2: 3.82 (0.52	Time 1: 911<br>Time 2: 726	Time 1: 3.72 (0.70)<br>Time 2: 3.76 (0.58)	Time x CREW Intervention [t-test (df)]:<br>1.95 (1634), p<0.05

**Tool or Scale**	Affective Commitment Scale
**Effect measure**	Mean (SD): Higher scores indicate greater commitment to the workplace
Outcome	**Commitment**

Intervention		***Control***		***Between Groups Comparison***
Sample size	*Results [Mean (SD)]*	*Sample size*	*Results [Mean (SD)]*	

Sample size	Results [Mean (SD)]	Sample size	Results [Mean (SD)]	Between Groups Comparison
Time 1: 262<br>Time 2: 181	Time 1: 3.16 (0.92)<br>Time 2: 3.49 (0.88)	Time 1: 911<br>Time 2: 726	Time 1: 3.30 (0.90)<br>Time 2: 3.43 (0.82)	Time x CREW Intervention [t-test (df)]:<br>2.41 (1634), p<0.05

Tool or Scale Maslach Burnout Inventory-General Survey (MBI-GS)

Effect measure Mean (SD): higher scores indicate more experiences of cynicism at work

Outcome **Maslach Burnout Inventory-General Survey (MBI-GS) – Cynicism subscale**

Intervention		***Control***		***Between Groups Comparison***
Sample size	*Results [Mean (SD)]*	*Sample size*	*Results [Mean (SD)]*	
Time 1: 262<br>Time 2: 181	Time 1: 1.89 (1.41)<br>Time 2: 1.36 (1.27)	Time 1: 911<br>Time 2: 726	Time 1: 1.65 (1.26)<br>Time 2: 1.55 (1.28)	Time x CREW Intervention [t-test (df)]:<br>-2.47 (1634), p<0.05

Outcome **Maslach Burnout Inventory-General Survey (MBI-GS) – Efficacy subscale**

Intervention		***Control***		***Between Groups Comparison***
Sample size	*Results [Mean (SD)]*	*Sample size*	*Results [Mean (SD)]*	
Time 1: 262<br>Time 2: 181	Time 1: 4.57 (0.98)<br>Time 2: 4.71 (0.95)	Time 1: 911<br>Time 2: 726	Time 1: 4.74 (0.89)<br>Time 2: 4.73 (0.94)	Time x CREW Intervention [t-test (df)]:<br>0.52 (1634), p=NS

Outcome **Maslach Burnout Inventory-General Survey (MBI-GS) – Exhaustion subscale**

Intervention		***Control***		***Between Groups Comparison***
Sample size	*Results [Mean (SD)]*	*Sample size*	*Results [Mean (SD)]*	
Time 1: 262<br>Time 2: 181	Time 1: 3.21 (1.57)<br>Time 2: 2.76 (1.49)	Time 1: 911<br>Time 2: 726	Time 1: 2.73 (1.42)<br>Time 2: 2.65 (1.42)	Time x CREW Intervention [t-test (df)]:<br>-1.53 (1634), p=NS

Tool or Scale Effort-Reward Imbalance Questionnaire (Esteem Reward section)

Effect measure Mean (SD): Higher scores indicate greater satisfaction with the workplace

Outcome **Satisfaction**

Intervention		***Control***		***Between Groups Comparison***
Sample size	*Results [Mean (SD)]*	*Sample size*	*Results [Mean (SD)]*	
Time 1: 262<br>Time 2: 181	Time 1: 5.06 (1.07)<br>Time 2: 5.62 (0.89)	Time 1: 911<br>Time 2: 726	Time 1: 5.32 (0.97)<br>Time 2: 5.47 (0.93)	Time x CREW Intervention [t-test (df)]:<br>4.12 (1634), p<0.05

**Tool or Scale**	Interpersonal Trust at Work Scale
**Effect measure**	Mean (SD): Higher scores indicate greater trust in managers and greater faith in manager competence
Outcome	**Trust in management subscale**

Intervention		***Control***		***Between Groups Comparison***
Sample size	*Results [Mean (SD)]*	*Sample size*	*Results [Mean (SD)]*	
Time 1: 262<br>Time 2: 181	Time 1: 2.98 (0.94)<br>Time 2: 3.34 (0.78)	Time 1: 911<br>Time 2: 726	Time 1: 3.19 (0.79)<br>Time 2: 3.33 (0.79)	Time x CREW Intervention [t-test (df)]:<br>2.12 (1634), p<0.05

**Tool or Scale**	Turnover Intentions Measure
**Effect measure**	Mean (SD): higher scores indicate a stronger intention to quit
Outcome	**Turnover intention**

Intervention		***Control***		***Between Groups Comparison***
Sample size	*Results [Mean (SD)]*	*Sample size*	*Results [Mean (SD)]*	
Time 1: 262<br>Time 2: 181	Time 1: 2.44 (1.00)<br>Time 2: 2.18 (0.94)	Time 1: 911<br>Time 2: 726	Time 1: 2.15 (0.93)<br>Time 2: 2.08 (0.90)	Time x CREW Intervention [t-test (df)]:<br>-0.67 (1634), p=NS

**Study**	**Mallette, 2011[14]**
**Overall Results**	Participation in the workbook, e-learning, and e-learning + virtual world interventions significantly increased knowledge related to horizontal violence. Participation in all interventions significantly increased self-confidence in responding to horizontal violence
**Tool or Scale**	Confidence questionnaire based on Bandura 2006
**Effect measure**	Mean change (SD): higher scores indicate increased self-confidence
Outcome	**Change in confidence (pre-post) in responding to a situation involving horizontal violence**

Intervention		***Control***		***Between Groups Comparison***
Sample size	*Results [Mean change (SD)]*	*Sample size*	*Results [Mean change (SD)]*	
Group 1: 30<br>Group 2: 34<br>Group 3: 31<br>Group 4: 31	Group 1: 18.3 (15.77)<br>Group 2: 17.9 (15.96)<br>Group 3: 17.0 (17.20)<br>Group 4: 20.8 (15.39)	NR	NR (NR)	Group 1 v Group 2 v Group 3 v Group 4:<br>F=0.31, p=0.82

**Tool or Scale**	Questionnaire on knowledge of horizontal violence

**Effect measure**	Mean change (SD): higher scores indicate increased knowledge of horizontal violence
Outcome	**Change in pre-to-post knowledge test scores**

Intervention		***Control***		***Between Groups Comparison***
Sample size	*Results [Mean change (SD)]*	*Sample size*	*Results [Mean change (SD)]*	
Group 1: 33<br>Group 2: 35<br>Group 3: 31<br>Group 4: 33	Group 1: 1.5 (2.21)<br>Group 2: 1.6 (1.65)<br>Group 3: 0.0 (1.51)<br>Group 4: 1.3 (2.41)	NR	NR (NR)	Group 1 v Group 2 v Group 3 v Group 4: F=4.41, p=.006

**Study**	**Meloni, 2011[15]**
**Overall Results**	The intervention increased employee engagement and raised awareness of workplace bullying (significance was not tested).
**Tool or Scale**	Employee satisfaction survey
**Effect measure**	Percentage of respondents who strongly agreed or agreed with the statement
Outcome	**Employee engagement**

Intervention		***Control***		***Between Groups Comparison***
Sample size	*Results [%]*	*Sample size*	*Results [%]*	
Pre: 421<br>Follow-up Time 1: 660<br>Follow-up Time 2: 710	Pre: Engaged- 28; Swinging voters- 45; Disengaged- 27<br>Follow-up Time 1: Engaged- 32; Swinging voters- 42; Disengaged- 26<br>Follow-up Time 2: Engaged- 37; Swinging voters- 43; Disengaged- 20	-	-	NA

Outcome	**There has been significant improvement in the last 12 months in management's preparedness to eliminate bullying and harassment**

Intervention		***Control***		***Between Groups Comparison***
Sample size	*Results [%]*	*Sample size*	*Results [%]*	
Pre: 421<br>Follow-up Time 1: 660<br>Follow-up Time 2: 710	Pre: NA<br>Follow-up Time 1: 58<br>Follow-up Time 2: 69	-	-	NA

Outcome	**If I observed or experienced bullying or harassment I would trust that, if such behaviour was reported, then it would be appropriately managed**

Intervention		***Control***		***Between Groups Comparison***
Sample size	*Results [%]*	*Sample size*	*Results [%]*	

Sample size	Results [%]	Sample size	Results [%]	Between Groups Comparison
Pre: 421<br>Follow-up Time 1: 660<br>Follow-up Time 2: 710	Pre: 44<br>Follow-up Time 1: 53<br>Follow-up Time 2: 64	-	-	NA

Outcome **If I observed or experienced bullying or harassment I would know how to go about reporting such behaviour**

Intervention		*Control*		*Between Groups Comparison*
Sample size	*Results [%]*	*Sample size*	*Results [%]*	
Pre: 421<br>Follow-up Time 1: 660<br>Follow-up Time 2: 710	Pre: 67<br>Follow-up Time 1: 79<br>Follow-up Time 2: 84	-	-	NA

Outcome **If I observed or experienced bullying or harassment I would report this behaviour**

Intervention		*Control*		*Between Groups Comparison*
Sample size	*Results [%]*	*Sample size*	*Results [%]*	
Pre: 421<br>Follow-up Time 1: 660<br>Follow-up Time 2: 710	Pre: 73<br>Follow-up Time 1: 77<br>Follow-up Time 2: 86	-	-	NA

Study **Osatuke, 2009[16]**

Overall Results The intervention significantly increased civility scores

Tool or Scale 8-item civility scale (Meterko et al., 2008)

Effect measure Mean (SD): Higher scores indicate greater perceptions of civility in the workplace

Outcome **Civility index scores**

Intervention		*Control*		*Between Groups Comparison*
Sample size	*Results [Mean (SD)]*	*Sample size*	*Results [SD]*	
Baseline: 1113<br>Post: 975	Baseline: 3.46 (0.99)<br>Post: 3.70 (0.93)	Baseline: 843<br>Post: 1087	Time 1: 3.46 (1.00)<br>Time 2: 3.46 (1.01)	ANOVA (Intervention status x time) [F-test (df)]: 13.35 (1), P<0.001

Study **Stagg, 2011[17]**

Overall Results The training program significantly increased knowledge of workplace bullying

Tool or Scale Testing of the training program

Effect measure Mean (SD): Higher scores indicate increased knowledge of workplace bullying

Outcome	**Knowledge Test Score**				
	Intervention		***Control***		***Between Groups Comparison***
Sample size	*Results [Mean (SD)]*		*Sample size*	*Results [Mean (SD)]*	
Pre: 15<br>Post: 15	Pre: 15.47 (1.06)<br>Post-test: 19.73 (1.10)<br>Pre v Post [t (df)]: -12.91 (14), p<0.01		-	-	NA

Behaviours Related to Workplace Bullying/Incivility

**Study**	**Ceravolo, 2012[2]**				
**Overall Results**	Positive trends in behavioural responses to verbal abuse, e.g., fewer nurses reported gossiping after the intervention				
**Tool or Scale**	Verbal Abuse Survey				
**Effect measure**	N (%): Proportion of respondents who agree or strongly agree with statement				
Outcome	**Were determined to solve the problem (in response to a verbally abusive incident)**				
	Intervention		***Control***		***Between Groups Comparison***
Sample size	*Results [N (%)]*		*Sample size*	*Results [N (%)]*	
Pre: 703<br>Post: 485	Pre: 194 (29)<br>Post: 170 (37.9)		-	-	NA
Outcome	**I have not gossiped in the past month**				
	Intervention		***Control***		***Between Groups Comparison***
Sample size	*Results [N (%)]*		*Sample size*	*Results [N (%)]*	
Pre: 703<br>Post: 485	Pre: 312 (40)<br>Post: 227 (47)		-	-	NA

**Study**	**Embree, 2013[6]**
**Overall Results**	Reduction in both self-silencing behaviours at work
**Tool or Scale**	Silencing the Self-Work Scale (STSS-W)
**Effect measure**	Mean(SD): Higher scores indicate behaviour congruent with self-silencing and conflict avoidance

Outcome	**Silencing the Self-Work Scale (STSS-W)**			
	Intervention		***Control***	***Between Groups Comparison***
Sample size	*Results [N (%)]*	*Sample size*	*Results [N (%)]*	
Pre: 44<br>Post: 33	Pre: 67.03(NR)<br>Post: 65.19(NR)	-	-	NA

**Study**	**Goldberg, 2007[8]**
**Overall Results**	NR (no results reported for this outcome)

**Study**	**Leiter, 2011[13]**			
**Overall Results**	Non-significant decrease in self-reports of instigating incivility.			
**Tool or Scale**	Researcher defined scale			
**Effect measure**	Mean (SD): "Using a 7-point Likert scale ranging from 1 (never) to 7 (daily), participants rated their own behavior"			
Outcome	**Instigated incivility**			
	Intervention		***Control***	***Between Groups Comparison***
Sample size	*Results [Mean (SD)]*	*Sample size*	*Results [Mean (SD)]*	
Time 1: 262<br>Time 2: 181	Time 1: 0.51 (0.49)<br>Time 2: 0.42 (0.43)	Time 1: 911<br>Time 2: 726	Time 1: 0.54 (0.54)<br>Time 2: 0.50 (0.55)	Time x CREW Intervention [t-test (df)]:<br>-1.12 (1634), p=NS

**Study**	**Sanderson, 2014[18]**			
**Overall Results**	Positive trends in the overall civility score and each item on the scale; only two items reached significance (Anti-discrimination and Co-operation)			
**Tool or Scale**	All Employee Survey - Civility Scale			
**Effect measure**	Mean (SD): higher scores indicate greater workplace civility [respondents rated agreement on 5-point Likert scale (1=strongly disagree)]			
Outcome	**Civility Scale - total score**			
	Intervention		***Control***	***Between Groups Comparison***
Sample size	*Results [Mean (SD)]*	*Sample size*	*Results [Mean (SD)]*	
Pre: NR<br>Post: NR	Pre: 3.30 (NR)<br>Post: 3.57 (NR)<br>Pre v Post [t-test]: F=3.74, t=1.496 (167), p=0.055	-	-	NA
Outcome	**Anti-Discrimination: "This organization does not tolerate discrimination."**			

	Intervention		Control		Between Groups Comparison
Sample size	*Results [Mean (SD)]*		*Sample size*	*Results [Mean (SD)]*	
Pre: 62<br>Post: 104	Pre: 3.45 (1.45)<br>Post: 3.62 (1.20)<br>Pre v Post [t-test]: F=4.95, t=-0.79 (164), p=0.027		-	-	NA
Outcome	**Conflict Resolution: "Disputes or conflicts are resolved fairly in my work group."**				
	Intervention		***Control***		***Between Groups Comparison***
Sample size	*Results [Mean (SD)]*		*Sample size*	*Results [Mean (SD)]*	
Pre: 60<br>Post: 101	Pre: 3.12 (1.43)<br>Post: 3.36 (1.21)<br>Pre v Post [t-test]: F=2.39, t=-1.13 (159), p=0.124		-	-	NA
Outcome	**Cooperation: "A spirit of cooperation and teamwork exists in my work group."**				
	Intervention		***Control***		***Between Groups Comparison***
Sample size	*Results [Mean (SD)]*		*Sample size*	*Results [Mean (SD)]*	
Pre: 62<br>Post: 105	Pre: 3.26 (1.41)<br>Post: 3.66 (1.18)<br>Pre v Post [t-test]: F=5.27, t=-1.96 (165), p=0.023		-	-	NA
Outcome	**Diversity Acceptance: "Managers/supervisors/team leaders work well with employees of different backgrounds in my work group."**				
	Intervention		***Control***		***Between Groups Comparison***
Sample size	*Results [Mean (SD)]*		*Sample size*	*Results [Mean (SD)]*	
Pre: 59<br>Post: 106	Pre: 3.53 (1.28)<br>Post: 3.70 (1.11)<br>Pre v Post [t-test]: F=1.67, t=-0.94 (163), p=0.198		-	-	NA
Outcome	**Personal Interest: "The people I work with take a personal interest in me."**				
	Intervention		***Control***		***Between Groups Comparison***
Sample size	*Results [Mean (SD)]*		*Sample size*	*Results [Mean (SD)]*	
Pre: 57<br>Post: 104	Pre: 3.04 (1.21)<br>Post: 3.39 (1.07)<br>Pre v Post [t-test]: F=0.45, t=-1.95 (159), p=0.504		-	-	NA
Outcome	**Reliability: "The people I work with can be relied on when I need help."**				
	Intervention		***Control***		***Between Groups Comparison***
Sample size	*Results [Mean (SD)]*		*Sample size*	*Results [Mean (SD)]*	

Pre: 61<br>Post: 106	Pre: 3.44 (1.13)<br>Post: 3.70 (1.16)<br>Pre v Post [t-test]: F=0.37, t=-1.39 (165), p=0.543	-	-	NA
Outcome	**Respect: "People treat each other with respect in my work group."**			
	Intervention	***Control***		***Between Groups Comparison***
Sample size	*Results [Mean (SD)]*	*Sample size*	*Results [Mean (SD)]*	
Pre: 63<br>Post: 104	Pre: 3.16 (1.46)<br>Post: 3.54 (1.30)<br>Pre v Post: F=3.43, t=-1.75 (165), p=0.066	-	-	NA
Outcome	**Value Differences: "Differences among individuals are respected and valued in my work group."**			
	Intervention	***Control***		***Between Groups Comparison***
Sample size	*Results [Mean (SD)]*	*Sample size*	*Results [Mean (SD)]*	
Pre: 58<br>Post: 104	Pre: 3.47 (1.30)<br>Post: 3.61 (1.20)<br>Pre v Post [t-test]: F=0.77, t=-0.69 (160), p=0.382	-	-	NA

**Study**	**Stagg, 2011[17]**			
**Overall Results**	Significant increase in the number of respondents that reported observing or engaging in bullying behaviour, possibly due to increased awareness of bullying, and that felt adequately trained to manage workplace bullying.			
**Tool or Scale**	Testing of the training program			
**Effect measure**	Mean (SD): respondents rated agreement on 5-point Likert scale (1=strongly disagree)			
Outcome	**"I have observed other nurses being bullied"**			
	Intervention	***Control***		***Between Groups Comparison***
Sample size	*Results [Mean (SD)]*	*Sample size*	*Results [Mean (SD)]*	
Pre: 15<br>Post: 15	Pre: NR<br>Post: NR<br>Pre v Post[Z-score]: -2.64, P<0.01	-	-	NA
Outcome	"I have bullied others"			
	Intervention	***Control***		***Between Groups Comparison***
Sample size	*Results [Mean (SD)]*	*Sample size*	*Results [Mean (SD)]*	
Pre: 15<br>Post: 15	Pre: NR<br>Post: NR<br>Pre v Post[Z-score]: -2.45, P<0.05	-	-	NA

Skills to Cope With Workplace Bullying/Incivility

**Study**	**Ceravolo, 2012[2]**			
**Overall Results**	Increased use of skills to resolve workplace issues			
**Tool or Scale**	Verbal Abuse Survey			
**Effect measure**	N (%): Proportion of respondents who agree or strongly agree with statement			
Outcome	**"I resolve conflict through direct conversation"**			
Intervention		***Control***		***Between Groups Comparison***
Sample size	*Results [N (%)]*	*Sample size*	*Results [N (%)]*	
Pre: 703<br>Post: 485	Pre: 381 (49)<br>Post: 277 (57)	-	-	NA

**Study**	**Stagg, 2011[17]**			
**Overall Results**	Perceived self-efficacy of skills significantly increased post-intervention			
**Tool or Scale**	Testing of the training program			
**Effect measure**	Mean (SD): respondents rated agreement on 5-point Likert scale (1=strongly disagree)			
Outcome	**"I am adequately trained to manage a workplace bully"**			
Intervention		***Control***		***Between Groups Comparison***
Sample size	*Results [Mean (SD)]*	*Sample size*	*Results [Mean (SD)]*	
Pre: 15<br>Post: 15	Pre: NR<br>Post: NR<br>Pre v Post[Z-score]: -3.36, P<0.01	-	-	NA
Outcome	**"I feel confident in defending myself against bullies"**			
Intervention		***Control***		***Between Groups Comparison***
Sample size	*Results [Mean (SD)]*	*Sample size*	*Results [Mean (SD)]*	
Pre: 15<br>Post: 15	Pre: NR<br>Post: NR<br>Pre v Post[Z-score]: NR, P=NS	-	-	NA

Results of Incivil Behaviour and Outcomes of Workplace Bullying

**Study**	**Anderson, 2006[19]**
**Overall Results**	Significant reduction in the number of emotional-verbal workplace violence events following training
**Tool or Scale**	Workplace Violence Questionnaire and Demographics tool
**Effect measure**	N (%): number of events over the study period

Outcome **Total number of workplace violence events**

Intervention		*Control*		*Between Groups Comparison*
Sample size	*Results [N (%)]*	*Sample size*	*Results [N (%)]*	
Pre: 22<br>Post: Complete training- 10;<br>Incomplete training- 5	Pre: 135 (67.5)<br>Post: Complete training- 29 (44.6);<br>Incomplete training- 15 (23)<br>Complete training Pre v Post [M, t-test (df)]: 2.100, 2.272 (9), p=0.49	Pre: 21<br>Post: 13	Pre: 65 (32.5)<br>Post: 21 (32.4)	Pre Intervention v Control [t-test (df)]: -2.721 (41), p=0.01

Outcome **Emotional-verbal workplace violence events**

Intervention		*Control*		*Between Groups Comparison*
Sample size	*Results [N (%)]*	*Sample size*	*Results [N (%)]*	
Pre: 22<br>Post: Complete training- 10;<br>Incomplete training- 5	Pre: 106 (78.5)<br>Post: Complete training- 21 (72.5);<br>Incomplete training- 10 (67)	Pre: 21<br>Post: 13	Pre: 49 (75.5)<br>Post: 19 (90.4)	Pre Intervention v Control [t-test (df)]: -3.032 (41), p=0.004<br>Post Complete training v Control [M, F-test (df)]: -4.323, 5.508 (2), p=0.01

Outcome **Physical workplace violence events**

Intervention		*Control*		*Between Groups Comparison*
Sample size	*Results [N (%)]*	*Sample size*	*Results [N (%)]*	
Pre: 22<br>Post: Complete training- 10;<br>Incomplete training- 5	Pre: 5 (4)<br>Post: Complete training- 0 (0);<br>Incomplete training- 2 (13)	Pre: 21<br>Post: 13	Pre: 1 (1.5)<br>Post: 1 (4.8)	-

Outcome **Physical workplace violence events**

Intervention		*Control*		*Between Groups Comparison*
Sample size	*Results [N (%)]*	*Sample size*	*Results [N (%)]*	
Pre: 22<br>Post: Complete training- 10;<br>Incomplete training- 5	Pre: 23 (17.5)<br>Post: Complete training- 8 (27.5);<br>Incomplete training- 3 (20)	Pre: 21<br>Post: 13	Pre: 15 (23)<br>Post: 1 (4.8)	-

**Study**	**Barak, 1994[20]**				
**Overall Results**	No reported serious events of sexual harassment after training (may be due to small follow-up sample size); no other evident trends				
**Tool or Scale**	Sexual Experiences Questionnaire				
**Effect measure**	N (%): number of people reporting exposure to the behaviour				
Outcome	**Verbal sexual harassment**				
	Intervention		***Control***		***Between Groups Comparison***
Sample size	*Results [N (%)]*		*Sample size*	*Results [N (%)]*	
Pre: 25<br>Post: 6	Pre: 13 (6)<br>Post: 6 (100)		-	-	-
Outcome	**Sexually suggestive behaviours**				
	Intervention		***Control***		***Between Groups Comparison***
Sample size	*Results [N (%)]*		*Sample size*	*Results [N (%)]*	
Pre: 25<br>Post: 6	Pre: NR (33)<br>Post: 3 (50)		-	-	-
Outcome	**Attempts of sexual bribery**				
	Intervention		***Control***		***Between Groups Comparison***
Sample size	*Results [N (%)]*		*Sample size*	*Results [N (%)]*	
Pre: 25<br>Post: 6	Pre: 2 (9)<br>Post: (0)		-	-	-
Outcome	**Sexual blackmail**				
	Intervention		***Control***		***Between Groups Comparison***
Sample size	*Results [N (%)]*		*Sample size*	*Results [N (%)]*	
Pre: 25<br>Post: 6	Pre: 2 (8)<br>Post: 0 (0)		-	-	-
Outcome	**Sexual assault**				
	Intervention		***Control***		***Between Groups Comparison***

Sample size	Results [N (%)]	Sample size	Results [N (%)]	
Pre: 25<br>Post: 6	Pre: 2 (8)<br>Post: 0 (0)	-	-	-

**Study**	**Ceravolo, 2012[2]**			
**Overall Results**	Overall reduction in the amount of verbal bullying and negative emotional responses (no formal test of significance)			
**Tool or Scale**	Verbal Abuse Survey			
**Effect measure**	N (%): number of participants reporting event			
Outcome	**Verbally abused at work**			
	Intervention		***Control***	***Between Groups Comparison***
Sample size	*Results [N (%)]*	*Sample size*	*Results [N (%)]*	
Pre: 703<br>Post: 485	Pre: 634 (90)<br>Post: 370 (76)	-	-	-
Outcome	**Felt confused in response to verbal abuse**			
	Intervention		***Control***	***Between Groups Comparison***
Sample size	*Results [N (%)]*	*Sample size*	*Results [N (%)]*	
Pre: 703<br>Post: 485	Pre: 116 (17.3)<br>Post: 98 (21.8)	-	-	-
Outcome	**Felt embarrassed in response to verbal abuse**			
	Intervention		***Control***	***Between Groups Comparison***
Sample size	*Results [N (%)]*	*Sample size*	*Results [N (%)]*	
Pre: 703<br>Post: 485	Pre: 275 (41)<br>Post: 193 (43)	-	-	-
Outcome	**Felt fearful in response to verbal abuse**			
	Intervention		***Control***	***Between Groups Comparison***
Sample size	*Results [N (%)]*	*Sample size*	*Results [N (%)]*	
Pre: 703<br>Post: 485	Pre: 63 (9.4)<br>Post: 116 (13.8)	-	-	-
Outcome	**Felt harassed in response to verbal abuse**			
	Intervention		***Control***	***Between Groups***

Sample size	Results [N (%)]	Sample size	Results [N (%)]	Comparison
Pre: 703<br>Post: 485	Pre: 272 (40.6)<br>Post: 174 (38.8)	-	-	-
Outcome	**Felt hostility in response to verbal abuse**			
	Intervention		***Control***	***Between Groups Comparison***
Sample size	*Results [N (%)]*	*Sample size*	*Results [N (%)]*	
Pre: 703<br>Post: 485	Pre: 96 (14.3)<br>Post: 59 (13.1)	-	-	-
Outcome	**Felt powerless in response to verbal abuse**			
	Intervention		***Control***	***Between Groups Comparison***
Sample size	*Results [N (%)]*	*Sample size*	*Results [N (%)]*	
Pre: 703<br>Post: 485	Pre: 121 (26.9)<br>Post: 238 (35.5)	-	-	-
Outcome	**Turnover rate**			
	Intervention		***Control***	***Between Groups Comparison***
Sample size	*Results [N (%)]*	*Sample size*	*Results [N (%)]*	
NR	Pre: NR (8.9)<br>Post: NR (6)	-	-	NA
Outcome	**Vacancy rate**			
	Intervention		***Control***	***Between Groups Comparison***
Sample size	*Results [N (%)]*	*Sample size*	*Results [N (%)]*	
NR	Pre: NR (8.9)<br>Post: NR (3)	-	-	NA
**Study**	**Chipps, 2012[3]**			
**Overall Results**	Non-significant increases in both reported bullying frequency and intensity after awareness/education training			
**Tool or Scale**	Negative Acts Questionnaire-Revised (NAQ-R)			
**Effect measure**	Mean (SD): number of negative acts experienced weekly or daily			

Outcome	**Bullying frequency score**			
	Intervention		***Control***	***Between Groups Comparison***
Sample size	*Results [Mean (SD)]*	*Sample size*	*Results [Mean (SD)]*	
Pre: 16<br>Post: 16	Pre: <1 (NR)<br>Post: 1.6 (NR)<br>Pre v Post [χ2]: 0.56, p=0.13	-	-	-
Outcome	**Bullying intensity score (based on cumulative number of negative acts reported)**			
	Intervention		***Control***	***Between Groups Comparison***
Sample size	*Results [Mean (SD)]*	*Sample size*	*Results [Mean (SD)]*	
Pre: 16<br>Post: 16	Pre: 7.6 (NR)<br>Post: 8.3 (NR)<br>Pre v Post [χ2]: NR, p=0.66	-	-	-
**Tool or Scale**	Negative Acts Questionnaire-Revised (NAQ-R)			
**Effect measure**	N (%): proportion of respondents that answered positively			
Outcome	**Have been witness to any bullying in the workplace**			
	Intervention		***Control***	***Between Groups Comparison***
Sample size	*Results [N (%)]*	*Sample size*	*Results [N (%)]*	
Pre: 16<br>Post: 16	Pre: 12 (75)<br>Post: 15 (88)	-	-	-
Outcome	**Self-identified as a target of bullying**			
	Intervention		***Control***	***Between Groups Comparison***
Sample size	*Results [N (%)]*	*Sample size*	*Results [N (%)]*	
Pre: 16<br>Post: 16	Pre: 3 (13)<br>Post: 4 (25)	-	-	-
Outcome	**Self-identifying as having experienced bullying at least weekly**			
	Intervention		***Control***	***Between Groups Comparison***
Sample size	*Results [N (%)]*	*Sample size*	*Results [N (%)]*	
Pre: 16<br>Post: 16	Pre: 6 (37)<br>Post: 1 (6)	-	-	-

Study	**Dahlby, 2014[4]**			
**Overall Results**	Significant reduction in the degree of seriousness of lateral violence post intervention, no other significant results or evident trends			
**Tool or Scale**	Lateral and Vertical Violence in Nursing Survey			
**Effect measure**	N (%): proportion of respondents that agree with each option			
Outcome	**Degree of seriousness of lateral violence in my work area: Lateral violence toward peer**			
	Intervention	***Control***		***Between Groups Comparison***
Sample size	*Results [N (%)]*	*Sample size*	*Results [N (%)]*	
Pre: 29<br>Post: 25	Not Serious: Pre- 12 (41.38); Post- 8 (33.33)<br>Serious: Pre- 8 (27.59); Post- 1 (4.17)<br>Somewhat Serious: Pre- 6 (20.69); Post- 13 (54.17)<br>Very Serious: Pre- 3 (10.34); Post- 2 (8.33)<br>Pre v Post [χ2]: NR, p=0.02	-	-	-
Outcome	**Experience with lateral violence in my work area: I observe lateral violence behaviours**			
	Intervention	***Control***		***Between Groups Comparison***
Sample size	*Results [N (%)]*	*Sample size*	*Results [N (%)]*	
Pre: 29<br>Post: 25	Daily: Pre- 5 (17.24); Post- 2 (8)<br>Weekly: Pre- 16 (55.17); Post- 12 (48)<br>Monthly: Pre- 7 (24.14); Post- 10 (40)<br>Never: Pre- 1 (3.45); Post- 1 (4)<br>Pre v Post [χ2]: NR, p=0.29	-	-	-
Outcome	**Experience with lateral violence in my work area: Recipient of lateral violence behaviours**			
	Intervention	***Control***		***Between Groups Comparison***
Sample size	*Results [N (%)]*	*Sample size*	*Results [N (%)]*	
Pre: 29<br>Post: 25	Daily: Pre- 2 (6.9); Post- 0 (0)<br>Weekly: Pre- 12 (41.38); Post- 12 (48)<br>Monthly: Pre- 4 (13.79); Post- 3 (12)<br>Never: Pre- 11 (37.93); Post- 10 (40)<br>Pre v Post [χ2]: NR, p=0.45	-	-	-
Outcome	**Experience with lateral violence in my work area: Use lateral violence behaviours**			
	Intervention	***Control***		***Between Groups Comparison***
Sample size	*Results [N (%)]*	*Sample size*	*Results [N (%)]*	

Pre: 29<br>Post: 25	Daily: Pre- 0 (0); Post- 0 (0)<br>Weekly: Pre- 4 (13.79); Post- 3 (12)<br>Monthly: Pre- 7 (24.14); Post- 12 (48)<br>Never: Pre- 18 (62.07); Post- 10 (40)<br>Pre v Post [χ2]: NR, p=0.17	-	-	-

**Study**	**Dompierre, 2008[5]**
**Overall Results**	Significant reduction in physical violence in intervention arm before and after intervention, significantly more psychologically violent events reported in intervention arm compared to control
**Tool or Scale**	Participant questionnaire
**Effect measure**	N (%): proportion of respondents that answered positively
Outcome	**Had been a victim of psychological violence**

Intervention		***Control***		***Between Groups Comparison***
Sample size	*Results [N (%)]*	*Sample size*	*Results [N (%)]*	
Pre: 97<br>Post: 51	Pre: 41 (41.2)<br>Post: 19 (37.25)<br>Pre v Post [χ2 (df)]: 0.22 (1), p=NS	Pre: NA<br>Post: 39	Pre: NA (NA)<br>Post: 8 (20.51)	Pre Intervention v Control [χ2 (df)]: NA<br>Post Intervention v Control [χ2 (df)]: 2.95 (1), p=NS

Outcome	**Had been a victim of physical violence**

Intervention		***Control***		***Between Groups Comparison***
Sample size	*Results [N (%)]*	*Sample size*	*Results [N (%)]*	
Pre: 96<br>Post: 51	Pre: 9 (9.4)<br>Post: 0 (0)<br>Pre v Post [χ2 (df)]: 5.09 (1), p<0.05	Pre: NA<br>Post: 39	Pre: NA (NA)<br>Post: 2 (5.13)	Pre Intervention v Control [χ2 (df)]: NA<br>Post Intervention v Control [χ2 (df)]: 2.67 (1), p=NS

**Tool or Scale**	Participant questionnaire
**Effect measure**	Mean (SD): number of events over the study period
Outcome	**Physically violent events**

Intervention		***Control***		***Between Groups Comparison***
Sample size	*Results [Mean (SD)]*	*Sample size*	*Results [Mean (SD)]*	

Pre: 101<br>Post: 49	Pre: 0.15 (NR)<br>Post: 0 (NR)<br>Pre v Post [t-test (df)]: 2.78 (148), p<0.01	Pre: NA<br>Post: 39	Pre: NA (NR)<br>Post: 0.05 (NR)	Pre Intervention v Control [t-test (df)]: NA<br>Post Intervention v Control [t-test (df)]: -1.43 (86), p=NS

Outcome	**Psychologically violent events**			
	Intervention	***Control***		***Between Groups Comparison***
Sample size	*Results [Mean (SD)]*	*Sample size*	*Results [Mean (SD)]*	
Pre: 100<br>Post: 49	Pre: 0.72 (NR)<br>Post: 0.84 (NR)<br>Pre v Post [t-test (df)]: -0.41 (147), p=NS	Pre: NA<br>Post: 39	Pre: NA (NR)<br>Post: 0.26 (NR)	Pre Intervention v Control [t-test (df)]: NA<br>Post Intervention v Control [t-test (df)]: 2.17 (86), p<0.05

**Study**	**Embree, 2013[6]**			
**Overall Results**	Continuing reduction in employee turnover post-intervention			
**Tool or Scale**	Human Resources data			
**Effect measure**	N (%): Defined as the total number of fulltime and part-time staff voluntarily separating from an organization during a calendar year			
Outcome	**Employee Turnover**			
	Intervention	***Control***		***Between Groups Comparison***
Sample size	*Results [N (%)]*	*Sample size*	*Results [N (%)]*	
Pre: NR<br>Year 1: NR<br>Year 2: NR	Pre: NR (7.84)<br>Year 1: NR (1.42)<br>Year 2: NR (0.00)	-	-	NA

**Study**	**Hoel, 2006[9]**			
**Overall Results**	Little to no changes on any measure, no evident trends			
**Tool or Scale**	Participant survey			
**Effect measure**	Mean (SD): higher scores indicate increased levels of the specified behaviour			
Outcome	**Bullied within the last six months**			
	Intervention	***Control***		***Between Groups Comparison***
Sample size	*Results [Mean (SD)]*	*Sample size*	*Results [Mean (SD)]*	

Group 1: Pre- NR; Post- NR<br>Group 2: Pre- NR; Post- NR<br>Group 3: Pre- NR; Post- NR<br>Group 4: Pre- NR; Post- NR	Group 1: Pre- 1.33 (NR); Post- 1.31 (NR)<br>Group 2: Pre- 1.23 (NR); Post- 1.27 (NR)<br>Group 3: Pre- 1.32 (NR); Post- 1.31 (NR)<br>Group 4: Pre- 1.46 (NR); Post-1.43 (NR)	Pre: NR<br>Post: NR	Pre: 1.25 (NR)<br>Post: 1.23 (NR)	-
Outcome	**Considered quitting last six months**			
Intervention		***Control***		***Between Groups Comparison***
Sample size	*Results [Mean (SD)]*	*Sample size*	*Results [Mean (SD)]*	
Group 1: Pre- NR; Post- NR<br>Group 2: Pre- NR; Post- NR<br>Group 3: Pre- NR; Post- NR<br>Group 4: Pre- NR; Post- NR	Group 1: Pre- 2.46 (NR); Post- 2.56 (NR)<br>Group 2: Pre- 2.45 (NR); Post- 2.47 (NR)<br>Group3: Pre- 2.42 (NR); Post- 2.41 (NR)<br>Group 4: Pre- 2.49 (NR); Post- 2.49 (NR)	Pre: NR<br>Post: NR	Pre: 2.46 (NR)<br>Post: 2.52 (NR)	-
Outcome	**Witnessed bullying over the last six months**			
Intervention		***Control***		***Between Groups Comparison***
Sample size	*Results [Mean (SD)]*	*Sample size*	*Results [Mean (SD)]*	
Group 1: Pre- NR; Post- NR<br>Group 2: Pre- NR; Post- NR<br>Group 3: Pre- NR; Post- NR<br>Group 4: Pre- NR; Post- NR	Group 1: Pre- 1.66 (NR); Post- 1.69 (NR)<br>Group 2: Pre- 1.62 (NR); Post- 1.49 (NR)<br>Group 3: Pre- 1.69(NR); Post- 1.59 (NR)<br>Group 4: Pre- 1.73 (NR); Post- 1.67 (NR)	Pre: NR<br>Post: NR	Pre: 1.63<br>Post: 1.63	-
**Tool or Scale**	Negative Acts Questionnaire-Revised			
**Effect measure**	Mean (SD): higher scores indicate increased negative behaviour			
Outcome	**Negative Acts Questionnaire**			
Intervention		***Control***		***Between Groups Comparison***
Sample size	*Results [Mean (SD)]*	*Sample size*	*Results [Mean (SD)]*	
Group 1: Pre- NR; Post- NR<br>Group 2: Pre- NR; Post- NR<br>Group 3: Pre- NR; Post- NR<br>Group 4: Pre- NR; Post- NR	Group 1: Pre- 29.29 (NR); Post- 28.96 (NR)<br>Group 2: Pre- 28.13 (NR); Post- 29.23 (NR)<br>Group 3: Pre- 27.76 (NR); Post- 27.89 (NR)<br>Group 4: Pre- 30.25 (NR); Post- 29.30 (NR)	Pre: NR<br>Post: NR	Pre: 28.57<br>Post: 28.63	-
**Tool or Scale**	Bullying Risk Assessment Tool (BRAT)			
**Effect measure**	Mean (SD): time off work in the last 6 months			

Outcome	**Self-reported absenteeism**			
Intervention		***Control***		***Between Groups Comparison***
Sample size	*Results [Mean (SD)]*	*Sample size*	*Results [Mean (SD)]*	
Group 1: Pre- NR; Post- NR<br>Group 2: Pre- NR; Post- NR<br>Group 3: Pre- NR; Post- NR<br>Group 4: Pre- NR; Post- NR	Group 1: Pre- 4.00 (NR); Post- 4.01 (NR)<br>Group 2: Pre- 4.19 (NR); Post- 3.61 (NR)<br>Group 3: Pre- 4.56 (NR); Post- 3.25 (NR)<br>Group 4: Pre- 3.67 (NR); Post- 3.56 (NR)	Pre: NR<br>Post: NR	Pre: 3.54 (NR)<br>Post: 3.72 (NR)	-

**Study**	**Keashly, 2009[21]**
**Overall Results**	NR (no results reported for this outcome)

**Study**	**Lansbury, 2014[12]**
**Overall Results**	Significant increase in reports of exposure to verbal bullying in intervention group 5, no other evident trends
**Tool or Scale**	Responsible Bystander Intervention in Verbal Bullying (RBI-VB)
**Effect measure**	Mean (SD): higher scores indicate greater exposure to verbal bullying

Outcome	**Self-reported exposure to workplace verbal bullying**			
Intervention		***Control***		***Between Groups Comparison***
Sample size	*Results [Mean (SD)]*	*Sample size*	*Results [Mean (SD)]*	
Group 1: Pre- NR; Post- NR<br>Group 2: Pre- NR; Post- NR<br>Group 3: Pre- NR; Post- NR<br>Group 4: Pre- NR; Post- NR<br>Group 5: Pre- NR; Post- NR<br>Group 6: Pre- NR; Post- NR	Group 1: Pre- 0.16 (0.373); Post- 0.18 (0.383);<br>Pre v Post [t-test]: 0.195, p=0.846<br>Group 2: Pre- 0.09 (0.281); Post- 0.11 (0.31);<br>Pre v Post [t-test]: 0.563, p=0.574<br>Group 3: Pre- 0.11 (0.316); Post- 0.17 (0.378);<br>Pre v Post [t-test]: 0.965, p=0.337<br>Group 4: Pre- 0.12 (0.329); Post- 0.05 (0.213);<br>Pre v Post [t-test]: 1.604, p=0.111<br>Group 5: Pre- 0.02 (0.125); Post- 0.14 (0.354);<br>Pre v Post [t-test]: 2.64, p=0.03<br>Group 6: Pre- 0.07 (0.249); Post- 0.08 (0.266);<br>Pre v Post [t-test]: 0.317, p=0.751	-	-	-

Outcome	**Self-reports of witnessing verbal bullying**			
Intervention		***Control***		***Between Groups Comparison***
Sample size	*Results [Mean (SD)]*	*Sample size*	*Results [Mean (SD)]*	

Group 1: Pre- NR; Post- NR<br>Group 2: Pre- NR; Post- NR<br>Group 3: Pre- NR; Post- NR<br>Group 4: Pre- NR; Post- NR<br>Group 5: Pre- NR; Post- NR<br>Group 6: Pre- NR; Post- NR	Group 1: Pre- 0.27 (0.447); Post- 0.23 (0.424);<br>Pre v Post [t-test]: 0.607, p=0.545<br>Group 2: Pre- 0.12 (0.329); Post- 0.14 (0.343);<br>Pre v Post [t-test]: 0.308, p=0.758<br>Group 3: Pre- 0.17 (0.38); Post- 0.19 (0.393);<br>Pre v Post [t-test]: 0.206, p=0.837<br>Group 4: Pre- 0.19 (0.394); Post- 0.11 (0.315);<br>Pre v Post [t-test]: 1.322, p=0.189<br>Group 5: Pre- 0.08 (0.27); Post- 0.12 (0.328);<br>Pre v Post [t-test]: 0.700, p=0.486<br>Group 6: Pre- 0.14 (0.353); Post- 0.09 (0.289);<br>Pre v Post [t-test]: 1.445, p=0.150	-	-	-

**Study**	**Leiter, 2011[13]**
**Overall Results**	Overall reduction in reports of supervisor and coworker incivility, increase in reports of respect in the workplace (no formal comparison made); significant decrease in self-reported absenteeism post-intervention
**Tool or Scale**	Workplace Incivility Scale
**Effect measure**	Mean (SD): Participants rated the extent to which they experienced incivility from others (0=never to 6=daily)
Outcome	**Co-worker incivility**

Intervention		***Control***		***Between Groups Comparison***
Sample size	*Results [Mean (SD)]*	*Sample size*	*Results [Mean (SD)]*	
Time 1: 262<br>Time 2: 181	Time 1: 0.78 (0.84)<br>Time 2: 0.58 (0.74)	Time 1: 911<br>Time 2: 726	Time 1: 0.8 (0.85)<br>Time 2: 0.76 (0.87)	-

Outcome	**Supervisor incivility**

Intervention		***Control***		***Between Groups Comparison***
Sample size	*Results [Mean (SD)]*	*Sample size*	*Results [Mean (SD)]*	
Time 1: 262<br>Time 2: 181	Time 1: 0.74 (0.99)<br>Time 2: 0.49 (0.8)	Time 1: 911<br>Time 2: 726	Time 1: 0.57 (0.85)<br>Time 2: 0.57 (0.85)	-

**Tool or Scale**	Effort-Reward Imbalance Questionnaire (Esteem Reward section)
**Effect measure**	Mean (SD): Participants rated agreement with statements (1=strongly disagree)
Outcome	**Respect (e.g., I receive the respect I deserve from my supervisors/colleagues)**

Intervention		***Control***		***Between Groups Comparison***
Sample size	*Results [Mean (SD)]*	*Sample size*	*Results [Mean (SD)]*	

Time 1: 262<br>Time 2: 181	Time 1: 3.21 (0.79)<br>Time 2: 3.62 (0.69)	Time 1: 911<br>Time 2: 726	Time 1: 3.42 (0.76)<br>Time 2: 3.51 (0.75)	-
**Tool or Scale**	Researcher developed questionnaire			
**Effect measure**	Mean (SD): number of self-reported absences per month			
Outcome	**Self-reported absences: "In the past month, on how many occasions have you missed work due to illness or disability?"**			
Intervention		***Control***		***Between Groups Comparison***
Sample size	*Results [Mean (SD)]*	*Sample size*	*Results [Mean (SD)]*	
Time 1: 262<br>Time 2: 181	Time 1: 0.88 (2.31)<br>Time 2: 0.54 (1.07)	Time 1: 911<br>Time 2: 726	Time 1: 0.86 (2.04)<br>Time 2: 0.83 (2.00)	Time x CREW Intervention [t-test (df)]:<br>-4.32 (1554), p<0.05
**Study**	**Leon-Perez, 2012[22]**			
**Overall Results**	NR (no results reported for this outcome)			
**Study**	**Meloni, 2011[15]**			
**Overall Results**	General increase in proportion of respondents that feel workplace is free from bullying and harassment			
**Tool or Scale**	Employee satisfaction survey			
**Effect measure**	N (%): proportion of respondents who agreed or strongly agreed with the statement			
Outcome	**"My workplace is free from bullying and harassment from my manager"**			
Intervention		***Control***		***Between Groups Comparison***
Sample size	*Results [N (%)]*	*Sample size*	*Results [N (%)]*	
Pre: 421<br>Time 1: 660<br>Time 2: 710	Pre: 295 (70)<br>Time 1: 449 (68)<br>Time 2: 525 (74)	-	-	-
**Study**	**Pate, 2010[23]**			
**Overall Results**	Significant reduction in perception of bullying as a problem in the organization after intervention			
**Tool or Scale**	Employee attitude survey			

**Effect measure**	Mean (SD): higher scores indicate stronger agreement with the statement
Outcome	**"I feel bullying is a problem within the organisation"**

Intervention		***Control***		***Between Groups Comparison***
Sample size	*Results [Mean (SD)]*	*Sample size*	*Results [Mean (SD)]*	
Pre: 126<br>Post: 120	Pre: 3.51<br>Post: 2.66<br>Pre v Post [t-test]: 6.10, p=0.00	-	-	-

**Tool or Scale**	Employee attitude survey
**Effect measure**	N (%): proportion of respondents who selected each option
Outcome	**Level of trust in senior management**

Intervention		***Control***		***Between Groups Comparison***
Sample size	*Results [Mean (SD)]*	*Sample size*	*Results*	
Pre: 126<br>Post: 120	Very Good: Pre- 5 (4); Post- 4 (3)<br>Good: Pre- 26 (21); Post- 26 (22)<br>Neutral: Pre- 30 (24); Post- 24 (20)<br>Adequate: Pre- 30 (24); Post- 26 (22)<br>Poor: Pre- 32 (25); 38 (32)	-	-	-

References

1. Bingham SG, Scherer LL. The unexpected effects of a sexual harassment educational program. J Appl Behav Sci. 2001;37(2):125-53.
2. Ceravolo DJ, Schwartz DG, Foltz-Ramos KM, Castner J. Strengthening communication to overcome lateral violence. J Nurs Manag. 2012;20(5):599-606. doi: http://dx.doi.org/10.1111/j.1365-2834.2012.01402.x.
3. Chipps EM, McRury M. The development of an educational intervention to address workplace bullying: a pilot study. J Nurses Staff Dev. 2012;28(3):94-8. doi: http://dx.doi.org/10.1097/NND.0b013e31825514bb.
4. Dahlby MA, Herrick LM. Evaluating an educational intervention on lateral violence. J Contin Educ Nurs. 2014;45(8):344-50; quiz 51-2. doi: http://dx.doi.org/10.3928/00220124-20140724-15.
5. Dompierre J, Laliberte D, Girard S, Gignac S. A qualitative and quantitative evaluation of an experiment for preventing violence in the workplace. Eur Rev Appl Psychol. 2008;58(4):275-83. doi: http://dx.doi.org/10.1016/j.erap.2008.09.010.
6. Embree JL, Bruner DA, White A. Raising the Level of Awareness of Nurse-to-Nurse Lateral Violence in a Critical Access Hospital. Nurs Res Pract. 2013;2013:207306. doi: http://dx.doi.org/10.1155/2013/207306.
7. Frisbie SH. Sexual harassment: A comparison of online versus traditional training methods. Dissertation Abstracts International: Section B: The Sciences and Engineering. 2002;62(10-B):4837. PubMed PMID: Dissertation Abstract: 2002-95008-310.
8. Goldberg CB. The impact of training and conflict avoidance on responses to sexual harassment. Psychol Women Q. 2007;31(1):62-72. doi: 10.1111/j.1471-6402.2007.00331.x.
9. Hoel H, Giga SI. Destructive interpersonal conflict in the workplace: The effectiveness of management interventions. Destructive Interpersonal Conflict in the Workplace: The Effectiveness of Management Interventions. 2006.
10. Hultman CS, Connolly A, Halvorson EG, Rowland P, Meyers MO, Mayer DC, et al. Get on your boots: preparing fourth-year medical students for a career in surgery, using a focused curriculum to teach the competency of professionalism. J Surg Res. 2012;177(2):217-23. doi: http://dx.doi.org/10.1016/j.jss.2012.06.019.
11. Kennedy M. Workplace bullying: The enculturated group behavior of nurses Southern Nazarene University.
12. Lansbury L. The development, measurement and implementation of a bystander intervention strategy: A field study on workplace verbal bullying in a large UK organisation: University of Portsmouth; 2014.
13. Leiter MP, Laschinger HKS, Day A, Oore DG. The impact of civility interventions on employee social behavior, distress, and attitudes. J Appl Psychol. 2011;96(6):1258-74. doi: 10.1037/a0024442.
14. Mallette C, Duff M, McPhee C, Pollex H, Wood A. Workbooks to virtual worlds: a pilot study comparing educational tools to foster a culture of safety and respect in Ontario. Nurs Leadersh (Tor Ont). 2011;24(4):44-64.
15. Meloni M, Austin M. Implementation and outcomes of a zero tolerance of bullying and harassment program. Aust Health Rev. 2011;35(1):92-4. doi: http://dx.doi.org/10.1071/AH10896.
16. Osatuke K, Moore SC, Ward C, Dyrenforth SR, Belton L. Civility, respect, engagement in the workforce (CREW): Nationwide organization development intervention at veterans health administration. J Appl Behav Sci. 2009;45(3):384-410. doi: 10.1177/0021886309335067.
17. Stagg SJ, Sheridan D, Jones RA, Speroni KG. Evaluation of a workplace bullying cognitive rehearsal program in a hospital setting. J Contin Educ Nurs. 2011;42(9):395-401; quiz 2-3. doi: http://dx.doi.org/10.3928/00220124-20110823-45.

18. Sanderson L. Improving civility in the mental health nursing workplace through assertiveness training with role-play. Dissertation Abstracts International: Section B: The Sciences and Engineering. 2014;74(11-B(E)):No Pagination Specified. PubMed PMID: Dissertation Abstract: 2014-99100-313.
19. Anderson C. Training efforts to reduce reports of workplace violence in a community health care facility. J Prof Nurs. 2006;22(5):289-95.
20. Barak A. A cognitive-behavioral educational workshop to combat sexual harassment in the workplace. J Couns Dev. 1994;72(6):595-602. doi: http://dx.doi.org/10.1002/j.1556-6676.1994.tb01688.x.
21. Keashly L, Neuman JH. Building a constructive communication climate: The Workplace Stress and Aggression Project. Destructive organizational communication: Processes, consequences, and constructive ways of organizing. New York, NY: Routledge/Taylor & Francis Group; US; 2009. p. 339-62.
22. Leon-Perez JM, Arenas A, Griggs TB. Effectiveness of conflict management training to prevent workplace bullying. Workplace bullying: Symptoms and solutions. New York, NY: Routledge/Taylor & Francis Group; US; 2012. p. 230-43.
23. Pate J, Beaumont P. Bullying and harassment: A case of success? Employee Relations. 2010;32(2):171-83. doi: http://dx.doi.org/10.1108/01425451011010113.
