# Supplementary material for: Prevention and management of unprofessional behaviour among adults in the workplace: A scoping review
Source: PLoS One. 2018 Jul 26;13(7):e0201187. doi: 10.1371/journal.pone.0201187 (PMC6062077; doi:10.1371/journal.pone.0201187)
Supplement: S6 Table — (PDF) [file pone.0201187.s006.pdf]

S6 Table. Measurement Tools and Scales

**Study**	**Anderson, 2006[1]**
**Name of tool**	*Workplace Violence Questionnaire and Demographics tool*
**Description**	"Only the WPV checklist and demographic information sheet were used in this study to assess work area violence...From a checklist of 29 WPV examples, workers were directed to select all events experienced over a 6-month period before and after training."
**Type**	Existing tool or scale
**Validated?**	Yes
**Outcome Category**	Results of Incivil Behaviour and Outcomes of Workplace Bullying
**Study**	**Barak, 1994[2]**
**Name of tool**	*Post-Workshop Evaluation*
**Description**	"...participants were asked to anonymously fill out a four-item questionnaire related to their satisfaction with the workshop...All items were rated on 7-point scales (1=low, 7=high)."
**Type**	Researcher developed questionnaire
**Validated?**	No
**Outcome Category**	NA
**Name of tool**	*Sexual Experiences Questionnaire*
**Description**	"Sexual Experiences Questionnaire (SEQ), adjusted to suit the local organizational structure. It contained 33 items related to various kinds of sexually harassing episodes, representing five levels of sexual harassment severity"
**Type**	Existing tool or scale
**Validated?**	No - revised, validated version published in 1995
**Outcome Category**	Results of Incivil Behaviour and Outcomes of Workplace Bullying
**Study**	**Bingham, 2001[3]**
**Name of tool**	*Questionnaire on knowledge and attitudes to sexual harassment*

**Description**	"We developed questionnaire items to assess how participation in the program affected employees' knowledge about sexual harassment, perceptions of potential sexual harassment, willingness to report sexual harassment, attributions of blame for sexual harassment, and attitudes toward sexual behavior at work."
**Type**	Researcher Developed Questionnaire
**Validated?**	No
**Outcome Category**	Knowledge of or Attitudes to Workplace Bullying/Incivility
**Study**	**Ceravolo, 2012[4]**
**Name of tool**	*Verbal Abuse Survey*
**Description**	Survey items were adapted from the Verbal Abuse Survey (Cox et al. 2007)…In the adapted instrument, nine item responses on a five-point Likert-type scale from Strongly Agree (=5) to Strongly Disagree (=1) are used to address perceptions of respect and lateral abuse within the nursing workgroup. Yes (1)/No (0) answers are provided for 10 items that addressed the presence of verbal abuse and feelings in response. Finally, participants are asked to rank their self-esteem and control over practice as Low (1), Medium (2) or High (3)."
**Type**	Existing tool or scale
**Validated?**	No formal validation - used in other surveys/research projects
**Outcome Category**	Knowledge of or Attitudes to Workplace Bullying/Incivility; Behaviours Related to Workplace Bullying/Incivility; Skills to Cope With Workplace Bullying/Incivility;<br>Results of Incivil Behaviour and Outcomes of Workplace Bullying
**Study**	**Chipps, 2012[5]**
**Name of tool**	*Participant survey*
**Description**	"The participants were given a 41-item survey, which included the Negative Acts Questionnaire-Revised (NAQ-R); demographic questions; and questions related to job satisfaction, errors, and near errors in clinical practice and patient satisfaction."
**Type**	Researcher developed questionnaire
**Validated?**	No
**Outcome Category**	Knowledge of or Attitudes to Workplace Bullying/Incivility; Results of Incivil Behaviour and Outcomes of Workplace Bullying
**Name of tool**	*Negative Acts Questionnaire-Revised (NAQ-R)*
**Description**	"The NAQ-R is a standardized tool developed to measure perceived exposure to workplace bullying as well as perceived victimization at work...The NAQ-R is a 22-item instrument that asks respondents how often they have experienced 22 behaviorally defined negative acts in the past 6 months (never, occasionally, monthly, weekly, and daily)."

**Type**	Existing tool or scale
**Validated?**	Yes
**Outcome Category**	Knowledge of or Attitudes to Workplace Bullying/Incivility; Results of Incivil Behaviour and Outcomes of Workplace Bullying
**Study**	**Dahlby, 2014[6]**
**Name of tool**	*Lateral and Vertical Violence in Nursing Survey*
**Description**	"The Lateral and Vertical Violence in Nursing Survey (Stanley et al., 2007) was used...For this project, the survey was modified to exclude questions pertaining to vertical violence in the work area."
**Type**	Existing tool or scale
**Validated?**	No
**Outcome Category**	Knowledge of or Attitudes to Workplace Bullying/Incivility; Results of Incivil Behaviour and Outcomes of Workplace Bullying
**Study**	**Dompierre, 2008[7]**
**Name of tool**	*Participant questionnaire*
**Description**	"...a questionnaire consisting of four parts was developed for the quantitative analysis…The first part measures a global index of the perception of the workplace, calculated from 20 questions answered on a four-point Likert type scale. The first twelve items dealt with the perception of the human and organizational setting...Four other statements examined the perception of the way in which the workplace dealt with violent events. The higher the score, the more the perception is positive. The second part of the questionnaire identified the episodes of physical and psychological violence that occurred in the context of or are linked to work..."
**Type**	Researcher developed questionnaire
**Validated?**	No
**Outcome Category**	Knowledge of or Attitudes to Workplace Bullying/Incivility; Results of Incivil Behaviour and Outcomes of Workplace Bullying
**Study**	**Embree, 2013[8]**
**Name of tool**	*Nurse Workplace Behaviour Scale (NWS)*
**Description**	"The NWS was arranged in a Likert-style format so that rating for agreement ranged from never to consistently, on a 1 to 5 scale.The questions asked how often a respondent engaged in specific activities, feelings, or beliefs which were examples of oppressed group behavior in nursing practice...possible ranges for the NWS Total Scale are 12 to 60..."
**Type**	Existing tool or scale

**Validated?**	Yes
**Outcome Category**	Knowledge of or Attitudes to Workplace Bullying/Incivility; Behaviours Related to Workplace Bullying/Incivility; Results of Incivil Behaviour and Outcomes of Workplace Bullying
**Name of tool**	*Silencing the Self-Work Scale (STSS-W)*
**Description**	"The STSS-W measures the ability to express personal needs or feelings directly or put others first. The STSS-W range is 25 (low) to 125 (high). Scoring higher on the STSSW may mean that the organization reinforces these behaviors"
**Type**	Existing tool or scale
**Validated?**	Yes
**Outcome Category**	Knowledge of or Attitudes to Workplace Bullying/Incivility; Behaviours Related to Workplace Bullying/Incivility; Results of Incivil Behaviour and Outcomes of Workplace Bullying
**Study**	**Frisbie, 2002[9]**
**Name of tool**	*Sexual Harassment Knowledge Scale*
**Description**	"Sexual harassment knowledge was assessed with a 20-item questionnaire. The number of items correctly answered was summed for each participant to ascertain sexual harassment knowledge. Scores could range from zero to twenty with higher scores indicating more knowledge acquisition"
**Type**	Researcher developed questionnaire
**Validated?**	No
**Outcome Category**	Knowledge of or Attitudes to Workplace Bullying/Incivility
**Name of tool**	*Sexual Harassment Knowledge Scale*
**Description**	"The SHAS is a 19-item scale on which participants indicate their degree of agreement with statements reflecting attitudes toward sexual harassment. Participants rated questions on a 6-point Likert-type scale ranging from strongly disagree to strongly agree."
**Type**	Existing tool or scale
**Validated?**	Yes
**Outcome Category**	Knowledge of or Attitudes to Workplace Bullying/Incivility
**Name of tool**	*Harassment Sensitivity Inventory*

**Description**	" The HSI consists of 18 situations (9 sexual and 9 nonsexual) and participants were asked to respond on a 6-point scale the degree they believe mildly to severely harassing behaviors are interfering, intimidating, hostile, and offensive. The mean of the item responses was used as the perception score in the current study"
**Type**	Existing tool or scale
**Validated?**	Yes
**Outcome Category**	Knowledge of or Attitudes to Workplace Bullying/Incivility
**Study**	**Goldberg, 2007[10]**
**Name of tool**	*Sexual Experiences Questionnaire*
**Description**	"The items reflected the gender harassment and unwanted sexual attention sub-factors of the Sexual Experiences Questionnaire, based on the work of Fitzgerald and her colleagues (cf. Fitzgerald, Gelfand, & Drasgow, 1995). The three gender-harassment items depicted a supervisor showing sexually suggestive cartoons, telling dirty (offensive) jokes, and making crude remarks about his/her sexual exploits. The three sexual-attention items depicted a supervisor repeatedly giving suggestive looks to a subordinate, asking a subordinate on a date, and sending romantic letters to a subordinate."
**Type**	Existing tool or scale
**Validated?**	No - reported reliability
**Outcome Category**	Knowledge of or Attitudes to Workplace Bullying/Incivility; Behaviours Related to Workplace Bullying/Incivility
**Study**	**Hoel, 2006[11]**
**Name of tool**	*Bullying Risk Assessment Tool (BRAT)*
**Description**	"...a new 29-items instrument, the Bullying Risk Assessment Tool (BRAT) emerged."
**Type**	Researcher developed questionnaire
**Validated?**	Partially - no independent study
**Outcome Category**	Knowledge of or Attitudes to Workplace Bullying/Incivility; Behaviours Related to Workplace Bullying/Incivility
**Name of tool**	*Negative Acts Questionnaire-Revised*
**Description**	"The 22-item NAQ-R (Einarsen & Hoel, 2001) was used to measure experiences of negative behaviour and bullying."

**Type**	Existing Tool or Scale
**Validated?**	Yes
**Outcome Category**	Results of Incivil Behaviour and Outcomes of Workplace Bullying
**Name of tool**	*Measure of psychological contract fulfillment*
**Description**	"To assess the state of employee psychological contracts we used Robinson’s (1996) 7-item measure of fulfilled employer obligations and 2-item measure of met employment expectations."
**Type**	Existing Tool or Scale
**Validated?**	No
**Outcome Category**	Knowledge of or Attitudes to Workplace Bullying/Incivility
**Name of tool**	*General Health Questionnaire*
**Description**	"Finally, mental health was measured using the 12-item General Health Questionnaire (Goldberg & Williams, 1988)."
**Type**	Existing Tool or Scale
**Validated?**	Yes
**Outcome Category**	Knowledge of or Attitudes to Workplace Bullying/Incivility
**Study**	**Hultman, 2012[12]**
**Name of tool**	*Pre-course survey on medical professionalism*
**Description**	"The purpose of this survey was to assess attitudes and knowledge about professionalism in medicine and surgery"
**Type**	Researcher developed questionnaire
**Validated?**	No
**Outcome Category**	Knowledge of or Attitudes to Workplace Bullying/Incivility

**Name of tool**	*Post-course evaluation*
**Description**	"The impact of the course on knowledge, skills,and attitudes was determined using self-reported, retrospective assessment measures."
**Type**	Researcher developed questionnaire
**Validated?**	No
**Outcome Category**	Knowledge of or Attitudes to Workplace Bullying/Incivility
**Study**	**Keashly, 2009[13]**
**Name of tool**	*Workplace Aggression Research Questionnaire*
**Description**	"The main section of the WAR-Q lists 60 different examples of workplace aggression that were derived from existing research on types of negative workplace communication and behavior... Respondents indicated the extent to which they experienced each type of behavior and the person most responsible for subjecting them to that behavior."
**Type**	Existing tool or scale
**Validated?**	No
**Outcome Category**	Results of Incivil Behaviour and Outcomes of Workplace Bullying
**Name of tool**	*Organizational Assessment Survery*
**Description**	"This instrument captures 17 different dimensions...In addition to these dimensions that are composite variables, the questionnaire captures overall assessments of supervisor or team leader performance, and satisfaction with the organization, pay, physical working conditions, and their job overall, as well as intentions to leave the organization."
**Type**	Existing tool or scale
**Validated?**	No
**Outcome Category**	Results of Incivil Behaviour and Outcomes of Workplace Bullying
**Study**	**Kennedy, 2010[14]**
**Name of tool**	*Demographic inventory and workplace bullying survey*
**Description**	"The pre-test consisted of 25 statements...The tests were scored based on a [5-point]Lickert scale. Five points were given for statements answered with strongly agree (SA), four points statements answered agree (A), [etc.]..."

**Type**	Researcher defined scale
**Validated?**	No
**Outcome Category**	Knowledge of or Attitudes to Workplace Bullying/Incivility
**Study**	**Lansbury, 2014[15]**
**Name of tool**	*Responsible Bystander Intervention in Verbal Bullying*
**Description**	"The Responsible Bystander Intervention in Verbal Bullying (RBI-VB) metric consisted of 15 statements; 3 sub-scales of 5 statements each...A single response was required for each of 15 RBI-VB statements; the possible score for each participant was therefore from 15 to 75...The greater the score the stronger the bystander responsibility for intervention in verbal bullying was."
**Type**	Researcher defined scale
**Validated?**	No
**Outcome Category**	Knowledge of or Attitudes to Workplace Bullying/Incivility; Results of Incivil Behaviour and Outcomes of Workplace Bullying
**Study**	**Leiter, 2011[16]**
**Name of tool**	*CREW Civility Scale*
**Description**	"The CREW Civility Scale (Meterko, Osatuke, Mohr, Warren, & Dyrenforth, 2007) consists of eight items designed to measure the perceptions of workplace civility within a work group and across an organization...The items were rated on a 5-point Likert scale ranging from 1 (strongly disagree) to 5 (strongly agree)."
**Type**	Existing tool or scale
**Validated?**	Partially - no independent study
**Outcome Category**	Knowledge of or Attitudes to Workplace Bullying/Incivility
**Name of tool**	*Workplace Incivility Scale*
**Description**	"The 10-item Workplace Incivility Scale (Cortina et al., 2001) assesses the frequency of health care workers' experiences of workplace incivility, including disrespectful, rude, or condescending behaviors in the previous month. Using a 7-point Likert scale ranging from 0 (never) to 6 (daily), participants rated the extent to which they experienced each of five behaviors...from their supervisor and from their coworkers."
**Type**	Existing tool or scale
**Validated?**	No

**Outcome Category**	Results of Incivil Behaviour and Outcomes of Workplace Bullying
**Name of tool**	*Interpersonal Trust at Work Scale (Trust in Management Subscale)*
**Description**	"Trust in management was measured by six items from Cook and Wall's (1980) Trust in Management subscale of the Interpersonal Trust at Work Scale. This scale measures two aspects of trust: (a) faith in manager intentions and (b) confidence in manager competence. Items are averaged to obtain scores ranging from 1 (strongly disagree) to 5 (strongly agree)."
**Type**	Existing tool or scale
**Validated?**	No
**Outcome Category**	Knowledge of or Attitudes to Workplace Bullying/Incivility
**Name of tool**	*Turnover Intentions Measure*
**Description**	"Three items were modified from the Turnover Intentions measure developed by Kelloway, Gottlieb, and Barham (1999) and used to assess the intention to quit...Each item was rated on a 5-point Likert scale ranging from 1 (strongly disagree) to 5 (strongly agree)."
**Type**	Existing tool or scale
**Validated?**	No
**Outcome Category**	Knowledge of or Attitudes to Workplace Bullying/Incivility
**Name of tool**	*Effort-Reward Imbalance Questionnaire (Esteem Reward section)*
**Description**	"Respect was measured using two items from the Esteem Reward section of the Effort-Reward Imbalance Questionnaire found in Siegrist et al. (2004)...A third item was created to capture organizational respect...Responses were rated on a 5-point scale ranging from 1 (strongly disagree) to 5 (strongly agree)."
**Type**	Existing tool or scale
**Validated?**	Yes
**Outcome Category**	Knowledge of or Attitudes to Workplace Bullying/Incivility or Attitudes
**Name of tool**	*Maslach Burnout Inventory-General Survery (MBI-GS: Professional Efficacy scale, Emotional Exhaustion and Cynicism subscales)*
**Description**	"The Emotional Exhaustion and Cynicism subscales of the Maslach Burnout Inventory-General Survey (MBI-GS; Maslach, Jackson, & Leiter, 1996; Schaufeli, Leiter, Maslach, & Jackson, 1996) were used to measure burnout. Participants used a 7-point Likert scale ranging from 0 (never) to 6 (every day) to rate the extent to which they experience exhaustion and cynicism at work..."

**Type**	Existing tool or scale
**Validated?**	Yes
**Outcome Category**	Knowledge of or Attitudes to Workplace Bullying/Incivility or Attitudes
**Name of tool**	*Affective commitement Scale*
**Description**	"Two items from the Affective Commitment Scale (Allen & Meyer, 1990) were used to assess organizational commitment. Using a 5-point Likert scale ranging from 1 (strongly disagree) to 5 (strongly agree), participants rated the extent to which they feel committed to their organization..."
**Type**	Existing tool or scale
**Validated?**	No
**Outcome Category**	Knowledge of or Attitudes to Workplace Bullying/Incivility
**Name of tool**	*Instigated Incivility*
**Description**	"Using a 7-point Likert scale ranging from 1 (never) to 7 (daily), participants rated their own behavior on each of the five items (e.g., "Ignored or excluded others from professional camaraderie")."
**Type**	Researcher defined scale
**Validated?**	No
**Outcome Category**	Behaviours Related to Workplace Bullying/Incivility
**Name of tool**	*Absenteeism*
**Description**	Self-reported absences were measured with a single item: "In the past month, on how many occasions have you missed work due to illness or disability?"
**Type**	Researcher defined scale
**Validated?**	No
**Outcome Category**	Behaviours Related to Workplace Bullying/Incivility
**Study**	**Leon-Perez, 2012[17]**

**Name of tool**	*Copenhagen Psychsocial Questionnaire*
**Description**	NR
**Type**	Existing tool or scale
**Validated?**	Yes
**Outcome Category**	Results of Incivil Behaviour and Outcomes of Workplace Bullying
**Name of tool**	*Interpersonal conflict at work (Medina et al., 2005)*
**Description**	NR
**Type**	Existing tool or scale
**Validated?**	Unclear - tool is composed of a number of pre-existing scales
**Outcome Category**	Results of Incivil Behaviour and Outcomes of Workplace Bullying
**Name of tool**	*Negative Acts Questionnaire-Revised*
**Description**	NR
**Type**	Existing tool or scale
**Validated?**	Yes
**Outcome Category**	Results of Incivil Behaviour and Outcomes of Workplace Bullying
**Name of tool**	*General Health Questionnaire*
**Description**	NR
**Type**	Existing tool or scale
**Validated?**	Yes

**Outcome Category**	Results of Incivil Behaviour and Outcomes of Workplace Bullying
**Study**	**Mallette, 2011[18]**
**Name of tool**	*Confidence questionnaire based on Bandura 2006*
**Description**	"In this study, a 100-point scale was used to measure professional confidence related to three items before before and after the educational intervention: (a) the ability to recognize horizontal violence when it occurs, (b) the ability to respond to a situation involving horizontal violence and (c) the ability to effectively modify the response to horizontal violence as the situation changes. The strength of efficacy beliefs was measured on a scale ranging in 10-unit intervals from 0 (cannot do) to 100 (highly certain can do)."
**Type**	Researcher defined scale
**Validated?**	No
**Outcome Category**	Knowledge of or Attitudes to Workplace Bullying/Incivility
**Name of tool**	*Questionnaire on knowledge of horizontal violence*
**Description**	"The pre and post knowledge test were developed by subject matter experts from within the organization…[it] consisted of 17 multiple-choice questions. The items measured the participant's knowledge of horizontal violence based on the learning objectives..."
**Type**	Researcher developed questionnaire
**Validated?**	Yes -content validity
**Outcome Category**	Knowledge of or Attitudes to Workplace Bullying/Incivility
**Name of tool**	*National League for Nursing (learner satisfaction)*
**Description**	"The learner's satisfaction with the assigned teaching methodology was evaluated using a sevn-point Likert scale ranging from one (strongly disagree) to seven (stongly agree)."
**Type**	Existing tool or scale
**Validated?**	Yes
**Outcome Category**	Knowledge of or Attitudes to Workplace Bullying/Incivility
**Name of tool**	*Global Rating Scale*

**Description**	"The Global Rating Scale for this study was a modified seven-point Likert scale examing the particpant's overall performance interacting with a standardized co-worker…Two evaluators observed each interaction and rated the participant's performance…"
**Type**	Researcher defined scale
**Validated?**	Yes
**Outcome Category**	Knowledge of or Attitudes to Workplace Bullying/Incivility
**Study**	**Meloni, 2011[19]**
**Name of tool**	*Employee satisfaction survey*
**Description**	NR
**Type**	Other
**Validated?**	No
**Outcome Category**	Knowledge of or Attitudes to Workplace Bullying/Incivility; Results of Incivil Behaviour and Outcomes of Workplace Bullying
**Study**	**Osatuke, 2009[20]**
**Name of tool**	*8-item civility scale (Meterko et al., 2008)*
**Description**	"Civility levels at the participating sites were measured by an 8-item civility scale (Meterko, Osatuke, Mohr, Warren, & Dyrenforth, 2007, 2008). The scale measures aspects of workplace civility through employee ratings of personal interest and respect from coworkers, cooperation or teamwork in the workgroup, fair conflict resolution, and valuing of individual differences by coworkers and supervisor."
**Type**	Existing tool or scale
**Validated?**	Partially - no independent study
**Outcome Category**	Knowledge of or Attitudes to Workplace Bullying/Incivility
**Study**	**Pate, 2010[21]**
**Name of tool**	*Employee attitude survey*
**Description**	NR

**Type**	Other
**Validated?**	No
**Outcome Category**	Results of Incivil Behaviour and Outcomes of Workplace Bullying
**Study**	**Sanderson, 2014[22]**
**Name of tool**	*All Employee Survey - Civility Scale*
**Description**	"The Civility Scale is an eight-item subscale embedded within the 71-item [All Employee Survey]...Each of the Civility Scale item statements were scored with a Likert-type scale: 1 (strongly disagree); 2 (disagree); 3 (neither agree nor disagree); 4 (agree); 5 (strongly agree); or 6 (do not know)."
**Type**	Existing tool or scale
**Validated?**	Yes
**Outcome Category**	Behaviours Related to Workplace Bullying/Incivility
**Study**	**Stagg, 2011[23]**
**Name of tool**	*Workplace Bullying Inventory*
**Description**	"...an adapted Workplace Bullying Inventory (WBI) was used...The 16-question WBI gathered information about bullying behaviors experienced during the previous year...The survey questions used a five-point Likert-type scale with responses ranging from never to daily. Two questions of interest to the researchers were added to the WBI. These questions asked whether the respondent had been pressured into doing something and asked the respondent to identify the major source of bullying."
**Type**	Existing tool or scale
**Validated?**	Yes
**Outcome Category**	Results of Incivil Behaviour and Outcomes of Workplace Bullying
**Name of tool**	*Testing of the training program*
**Description**	"The pre- and posttests were identical and included 25 questions (Appendix A; www.slackjournals.com/jcen). Twenty-one of the questions pertained to the information covered in the training. The other four questions asked about the staff nurse's attitude toward bullying experiences, adequacy of training in managing a bully, and confidence in defending oneself against a bully."
**Type**	Researcher Developed Questionnaire
**Validated?**	No

**Outcome Category**	Knowledge of or Attitudes to Workplace Bullying/Incivility; Behaviours Related to Workplace Bullying/Incivility; Skills to Cope With Workplace Bullying/Incivility

References

1. Anderson C. Training efforts to reduce reports of workplace violence in a community health care facility. J Prof Nurs. 2006;22(5):289-95.

2. Barak A. A cognitive-behavioral educational workshop to combat sexual harassment in the workplace. J Couns Dev. 1994;72(6):595-602. doi: http://dx.doi.org/10.1002/j.1556-6676.1994.tb01688.x.

3. Bingham SG, Scherer LL. The unexpected effects of a sexual harassment educational program. J Appl Behav Sci. 2001;37(2):125-53.

4. Ceravolo DJ, Schwartz DG, Foltz-Ramos KM, Castner J. Strengthening communication to overcome lateral violence. J Nurs Manag. 2012;20(5):599-606. doi: http://dx.doi.org/10.1111/j.1365-2834.2012.01402.x.

5. Chipps EM, McRury M. The development of an educational intervention to address workplace bullying: a pilot study. J Nurses Staff Dev. 2012;28(3):94-8. doi: http://dx.doi.org/10.1097/NND.0b013e31825514bb.

6. Dahlby MA, Herrick LM. Evaluating an educational intervention on lateral violence. J Contin Educ Nurs. 2014;45(8):344-50; quiz 51-2. doi: http://dx.doi.org/10.3928/00220124-20140724-15.

7. Dompierre J, Laliberte D, Girard S, Gignac S. A qualitative and quantitative evaluation of an experiment for preventing violence in the workplace. Eur Rev Appl Psychol. 2008;58(4):275-83. doi: http://dx.doi.org/10.1016/j.erap.2008.09.010.

8. Embree JL, Bruner DA, White A. Raising the Level of Awareness of Nurse-to-Nurse Lateral Violence in a Critical Access Hospital. Nurs Res Pract. 2013;2013:207306. doi: http://dx.doi.org/10.1155/2013/207306.

9. Frisbie SH. Sexual harassment: A comparison of online versus traditional training methods. Dissertation Abstracts International: Section B: The Sciences and Engineering. 2002;62(10-B):4837. PubMed PMID: Dissertation Abstract: 2002-95008-310.

10. Goldberg CB. The impact of training and conflict avoidance on responses to sexual harassment. Psychol Women Q. 2007;31(1):62-72. doi: 10.1111/j.1471-6402.2007.00331.x.

11. Hoel H, Giga SI. Destructive interpersonal conflict in the workplace: The effectiveness of management interventions. Destructive Interpersonal Conflict in the Workplace: The Effectiveness of Management Interventions. 2006.

12. Hultman CS, Connolly A, Halvorson EG, Rowland P, Meyers MO, Mayer DC, et al. Get on your boots: preparing fourth-year medical students for a career in surgery, using a focused curriculum to teach the competency of professionalism. J Surg Res. 2012;177(2):217-23. doi: http://dx.doi.org/10.1016/j.jss.2012.06.019.

13. Keashly L, Neuman JH. Building a constructive communication climate: The Workplace Stress and Aggression Project. Destructive organizational communication: Processes, consequences, and constructive ways of organizing. New York, NY: Routledge/Taylor & Francis Group; US; 2009. p. 339-62.

14. Kennedy M. Workplace bullying: The enculturated group behavior of nurses Southern Nazarene University.

15. Lansbury L. The development, measurement and implementation of a bystander intervention strategy: A field study on workplace verbal bullying in a large UK organisation: University of Portsmouth; 2014.

16. Leiter MP, Laschinger HKS, Day A, Oore DG. The impact of civility interventions on employee social behavior, distress, and attitudes. J Appl Psychol. 2011;96(6):1258-74. doi: 10.1037/a0024442.

17. Leon-Perez JM, Arenas A, Griggs TB. Effectiveness of conflict management training to prevent workplace bullying. Workplace bullying: Symptoms and solutions. New York, NY: Routledge/Taylor & Francis Group; US; 2012. p. 230-43.

18. Mallette C, Duff M, McPhee C, Pollex H, Wood A. Workbooks to virtual worlds: a pilot study comparing educational tools to foster a culture of safety and respect in Ontario. Nurs Leadersh (Tor Ont). 2011;24(4):44-64.

19. Meloni M, Austin M. Implementation and outcomes of a zero tolerance of bullying and harassment program. Aust Health Rev. 2011;35(1):92-4. doi: http://dx.doi.org/10.1071/AH10896.

20. Osatuke K, Moore SC, Ward C, Dyrenforth SR, Belton L. Civility, respect, engagement in the workforce (CREW): Nationwide organization development intervention at veterans health administration. J Appl Behav Sci. 2009;45(3):384-410. doi: 10.1177/0021886309335067.

21. Pate J, Beaumont P. Bullying and harassment: A case of success? Employee Relations. 2010;32(2):171-83. doi: http://dx.doi.org/10.1108/01425451011010113.

22. Sanderson L. Improving civility in the mental health nursing workplace through assertiveness training with role-play. Dissertation Abstracts International: Section B: The Sciences and Engineering. 2014;74(11-B(E)):No Pagination Specified. PubMed PMID: Dissertation Abstract: 2014-99100-313.

23. Stagg SJ, Sheridan D, Jones RA, Speroni KG. Evaluation of a workplace bullying cognitive rehearsal program in a hospital setting. J Contin Educ Nurs. 2011;42(9):395-401; quiz 2-3. doi: http://dx.doi.org/10.3928/00220124-20110823-45.
