# Supplementary material for: Prevention and management of unprofessional behaviour among adults in the workplace: A scoping review
Source: PLoS One. 2018 Jul 26;13(7):e0201187. doi: 10.1371/journal.pone.0201187 (PMC6062077; doi:10.1371/journal.pone.0201187)
Supplement: S1 Text — (PDF) [file pone.0201187.s008.pdf]

S1 Text. MEDLINE Search Strategy

Database: Ovid MEDLINE(R) In-Process & Other Non-Indexed Citations and Ovid MEDLINE(R) <1946 to Present>

Search Strategy:

----

1 Workplace Violence
2 Workplace
3 Health Facility Environment
4 exp *professional role
5 (professional role or professionalism or job site$ or work location$ or work place$ or workplace$ or work-place$ or work site$ or work site$ or work-site$ or health facilit$ or healthcare facilit$ or health-care facilit$).tw.
6 occupational health
7 or/2-6
8 exp Aggression
9 social marginalization
10 *social isolation
11 exp Violence
12 (bullying or bully or bullies or cyberbull$ or cyber-bull$ or mobbing or social exclusion or harass$ or violence or abus$ or covert attitude$ or hostilit$ or obstructionism or aggression or aggressive behavior$ or aggressive behaviour$ or exclusion or victimi#ation or intimidate$ or physical threat$).tw.
13 Dominance-Subordination
14 hostility
15 or/8-14
16 7 and 15
17 1 or 16
18 randomized controlled trial.pt.
19 controlled clinical trial.pt.
20 (randomized or placebo).mp.
21 experiment$.tw.
22 (time adj series).tw.
23 (pre test or pretest or (posttest or post test)).tw.
24 random allocation
25 impact.tw.
26 intervention$.tw.
27 chang$.tw.
28 prevention program$.tw.
29 evaluation studies
30 evaluat$.tw.
31 (effect or effective).tw.
32 comparative studies
33 comparative study.pt.
34 or/18-32
35 17 and 34

Filter: used EPOC filter (unpublished) – this includes Best balance of sensitivity and specificity for therapy trials from: http://hiru.mcmaster.ca/hiru/HIRU_Hedges_MEDLINE_Strategies.asp
