# Supplementary material for: Prevention and management of unprofessional behaviour among adults in the workplace: A scoping review
Source: PLoS One. 2018 Jul 26;13(7):e0201187. doi: 10.1371/journal.pone.0201187 (PMC6062077; doi:10.1371/journal.pone.0201187)
Supplement: S2 Text — (PDF) [file pone.0201187.s009.pdf]

S2 Text. List of sites searched for grey literature

Bullying related

- Bullying Research Network (BRNET): http://cehs15.unl.edu/cms/index.php?s=2&p=124
- Canadian Centre for Occupational Health and Safety: https://www.ccohs.ca/oshanswers/psychosocial/bullying.html
- Canada Safety Council: https://canadasafetycouncil.org
- CDC (Occupational Violence): http://www.cdc.gov/niosh/topics/violence/
- ebrary's Searchable Information Center on Cyberbullying: http://site.ebrary.com/lib/cyberbullying/home.action
- Job safety: http://jobsafety.seton.ca/latest-news/
- Ontario Prevention clearing house: http://www.ohpe.ca/node/4765
- Ontario Ministry of Labour: http://www.labour.gov.on.ca/english/hs/sawo/pubs/fs_workplaceviolence.php
- PREVNET: http://www.prevnet.ca
  - Check resources link: http://www.prevnet.ca/resources
  - Check websites link: http://www.prevnet.ca/resources/websites
- Safetylit: http://www.safetylit.org
- Workplace Bullying Institute: http://www.workplacebullying.org
- Workplace Mental Health Promotion: http://wmhp.cmhaontario.ca/workplace-mental-health-core-concepts-issues/issues-in-the-workplace-that-affect-employee-mental-health/harassment-violence-bullying-and-mobbing
- Work Safe BC: http://www.worksafebc.com

General Grey

- Government of Canada: http://publications.gc.ca/site/eng/search/eCollection.html
- GreyNet International: http://www.greylit.org
- SIGLE (System for Information on Grey Literature in Europe): http://www.opengrey.eu
- National Technical Information Service (NTIS): http://www.ntis.gov

Guidelines and evidence-based resources

- Campbell Collaboration: http://www.campbellcollaboration.org
- Canadian best practices portal: http://cbpp-pcpe.phac-aspc.gc.ca/~cbpp/dev/
- Health Evidence: http://www.healthevidence.org
- National Guidelines Clearinghouse: http://www.guideline.gov
- NICE: https://www.nice.org.uk
- SIGN: http://www.sign.ac.uk

International

- Agency for Healthcare Research and Quality: http://www.ahrq.gov/research/index.html
- LILACS - Latin-American and Caribbean Center on Health Sciences Information: http://lilacs.bvsalud.org/en/
- WHO (WHOLIS): http://dosei.who.int/uhtbin/cgisirsi/Tue+Apr++5+17:45:43+MEST+2016/0/49

Search engines

- TRIP database: http://www.tripdatabase.com/
- Google: https://www.google.ca/advanced_search
- Google Scholar: https://scholar.google.com/intl/en/scholar/about.html

Thesis

- Center for Research Libraries Foreign Dissertation: https://www.crl.edu/collections/topics/dissertations
- DART-Europe E-theses Portal: http://www.dart-europe.eu/basic-search.php
- Electronic Theses Online Service (ETHOS) | British Library: http://ethos.bl.uk/Home.do;jsessionid=D96E9CF245B0FE0199DDDB94FF4BD2A7
- *Open access dissertations: https://oatd.org
- Thesis Canada Portal: http://www.bac-lac.gc.ca/eng/services/theses/Pages/theses-canada.asp
