# Supplementary material for: Prevention and management of unprofessional behaviour among adults in the workplace: A scoping review
Source: PLoS One. 2018 Jul 26;13(7):e0201187. doi: 10.1371/journal.pone.0201187 (PMC6062077; doi:10.1371/journal.pone.0201187)

S1 Fig Word cloud of terms used in definitions of Workplace bullying/Incivility

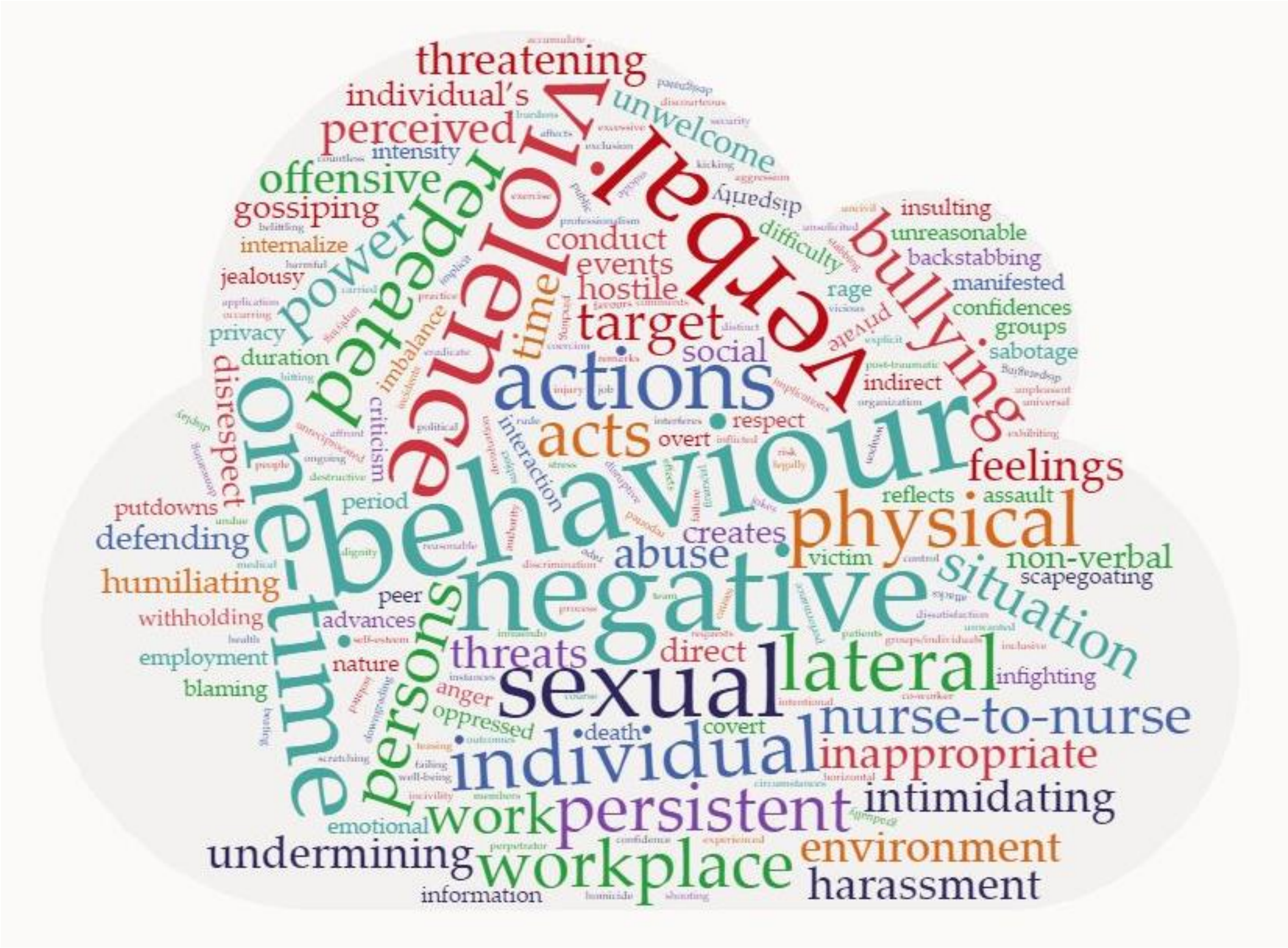

Supplement: S1 Fig — (PDF) [file pone.0201187.s007.pdf]
